# Fluorinated Hexosome Carriers for Enhanced Solubility of Drugs

**DOI:** 10.1021/jacsau.5c00198

**Published:** 2025-04-28

**Authors:** Tiffany Guitton-Spassky, Boris Schade, Christian Zoister, Eleonora Veronese, Marta Rosati, Francesca Baldelli Bombelli, Gabriella Cavallo, Andreas F. Thünemann, Hassan Ghermezcheshme, Hesam Makki, Roland R. Netz, Kai Ludwig, Pierangelo Metrangolo, Abhishek Kumar Singh, Rainer Haag

**Affiliations:** † Institut für Chemie und Biochemie, Organische Chemie, 9166 Freie Universität Berlin, Takustraße 3, 14195 Berlin, Germany; ‡ Institut für Chemie und Biochemie, Forschungszentrum für Elektronenmikroskopie, 9166 Freie Universität Berlin, Fabeckstraße 36a, 14195 Berlin, Germany; § Laboratory of Supramolecular and Bio-Nanomaterials (SBNLab), Department of Chemistry, Materials and Chemical Engineering “Giulio Natta”, Politecnico di Milano, via Luigi Mancinelli 7, 20131 Milan, Italy; ∥ German Federal Institute for Materials Research and Testing (BAM), Unter den Eichen 87, 12205 Berlin, Germany; ⊥ Department of Polymer and Color Engineering, Amirkabir University of Technology, 424 Hafez Avenue, Tehran 15875-4413, Iran; # Department of Chemistry and Materials Innovation Factory, University of Liverpool, L69 7ZD Liverpool, U.K.; ∇ Department of Physics, 9166 Freie Universität Berlin, Arnimallee 14, 14195 Berlin, Germany

**Keywords:** branched, fluorinated, supramolecular, self-assembly, amphiphile, hexosome, encapsulation

## Abstract

Designing nanomaterials for drug encapsulation is a crucial, yet challenging, aspect for pharmaceutical development. An important step is synthesizing amphiphiles that form stable supramolecular systems for efficient drug loading. In the case of fluorinated drugs, these have superior properties and also a tendency toward reduced water solubility. For the first time, we report here fluorinated hexosome carriers made from nonionic dendritic amphiphiles, capable of encapsulating the fluorinated drug Leflunomide with high efficiency (62 ± 3%) and increasing its solubility by 12-fold. We synthesized amphiphiles with varying tail groups (fluorinated/alkylated), and their supramolecular self-assembly was investigated using cryogenic transmission electron microscopy and small-angle X-ray scattering. Furthermore, Leflunomide and its equivalent nonfluorinated counterpart were encapsulated within fluorinated and nonfluorinated assemblies. Self-assembly and encapsulation mechanisms were well supported by coarse-grained molecular simulations, yielding a fundamental understanding of the new systems.

## Graphical abstract

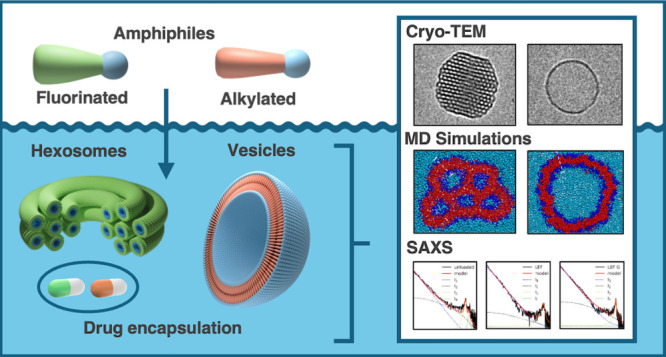

## Introduction

The efficiency of pharmaceuticals in medical therapy depends on how well they reach their desired target in the body. For small-molecule drugs, efficiency often relies on their solubility in bodily fluids and, by extension, their pharmacokinetics.^1,2^ This is particularly important for fluorinated small-molecule drugs, which constitute approximately 20% of pharmaceuticals.^3^ One or more fluorine atoms may be included in drug structures to impart advantageous properties, such as improved metabolic stability, altered physicochemical properties, or increased binding affinity,^4^ although this often leads to a lower water solubility, in particular for drugs containing −CF_3_ groups.^5–7^ Among different strategies to improve drug solubility, encapsulation within liposomes or polymeric nanoparticles represents an interesting possibility.^8^

In recent years, researchers have investigated novel supramolecular carriers with more complex 3D architectures. Among the most notable candidates are cubosomes and hexosomes, which are nonlamellar lyotropic liquid crystalline nanoparticles. These carriers can encapsulate both hydrophilic and hydrophobic compounds with high efficiency, release them over longer periods of time, and are chemically and mechanically stable.^9,10^ Cubosomes consist of a bicontinuous amphiphile layer and two nonintersecting water channels with different cubic symmetries. On the other hand, hexosomes consist of hexagonally stacked water channels surrounded by an amphiphile layer. Nonlamellar systems are relatively newer than lamellar ones, and amphiphile structures have largely been limited to polymers and lipids.^9,11^ A wider range of available amphiphiles would expand the possibilities for modulating system properties, while also further clarifying the behavior, advantages, and limitations of these interesting nonlamellar systems.

Fluorinated hydrophobic tails impart advantageous properties, such as increased membrane stability upon heating and better retention of encapsulated drugs, making them relevant for drug encapsulation applications.^12^ The fluorinated amphiphile molecules are more chemically and thermally stable than their alkylated analogues, and their self-assemblies have a higher stability and form better-defined supramolecular architectures.^13–23^ This is partially due to their larger size, conformational rigidity, and the formation of a separate fluorous phase,^24^ i.e., the fluorophobic effect.^25^ Making use of this phase separation, fluorinated carriers, mostly micelles and vesicles, have been used to encapsulate fluorinated small-molecule compounds with high encapsulation efficiency and sustained release, in order to improve their performance.^26–31^

Short perfluorinated chains are expected to reduce the bioaccumulation due to their high mobility.^32^ Moreover, fluorous phase separation can be reinforced by dendritic structures.^33^ Recently, both these structural elements were implemented in a branched moiety containing 27 fluorine atoms on perfluoro-*tert*-butyl groups, linked to the dendritic core by ether linkages.^34–36^ Aside from the high fluorine content, the size of the perfluorinated residue is increased compared with a CH_3_ group. According to the concept of the critical packing parameter,^37^ self-assemblies like reversed micelles, cubosomes, or hexosomes should be formed if the hydrophobic tail is larger than the hydrophilic headgroup.^9^ This concept can be applied to develop novel fluorinated carriers with complex architectures and improved properties, where both the advantages of fluorinated and nonlamellar carriers are combined. There exists only one description of fluorinated polymers forming fluorinated cubosomes.^38^ Fluorinated liquid crystalline cubic phases have been previously reported, but they were not nanoparticulate.^39^ Therefore, despite some advances, reports on fluorinated nonlamellar lyotropic liquid crystalline nanoparticles like hexosomes and cubosomes remain scarce.

In this work, we synthesized branched perfluorinated and alkylated amphiphiles, evaluated their self-assembly in water, and tested the resulting assembled systems for suitability in drug encapsulation applications. Our goal was to investigate the role and potential use of fluorine inclusion within amphiphilic structures to transport fluorinated drugs within hexosomes. Four dendritic amphiphiles were prepared, with different branched tail groups (fluorinated or alkylated), linkers (amide or triazole), and—as a hydrophilic head—a generation 1 oligo-glycerol dendron (G1) (Figure 1). To obtain the alkylated equivalent of the branched fluorinated tail group, we developed a new synthetic route so that we could investigate the effect of fluorine on the assembly. The self-assembly behavior was characterized using dynamic light scattering (DLS), cryogenic transmission electron microscopy (cryo-TEM), and small-angle X-ray scattering (SAXS), and then further confirmed using coarse-grained simulations. We established that both the fluorination and the different linkers affected the self-assembly, and importantly, we report here for the first time the formation of fluorinated hexosomes. A fluorinated drug, Leflunomide (LEF), and its nonfluorinated equivalent, Leflunomide Impurity G (LEF G), were encapsulated in both fluorinated and nonfluorinated self-assemblies, and the encapsulation efficiency (EE%) was determined. Using SAXS and simulations, we investigated the role of fluorine within these new nanocarriers and its effect on the molecular level. Lastly, the cytotoxicity was verified, which was significantly lower for the fluorinated amphiphiles compared with the alkylated counterparts (Figure S19). These results confirm the potential of branched fluorinated amphiphile self-assemblies as nanocarriers for fluorinated compounds and uncover for the first time a new type of 3D nanostructure: fluorinated hexosomes.

**Figure 1:**
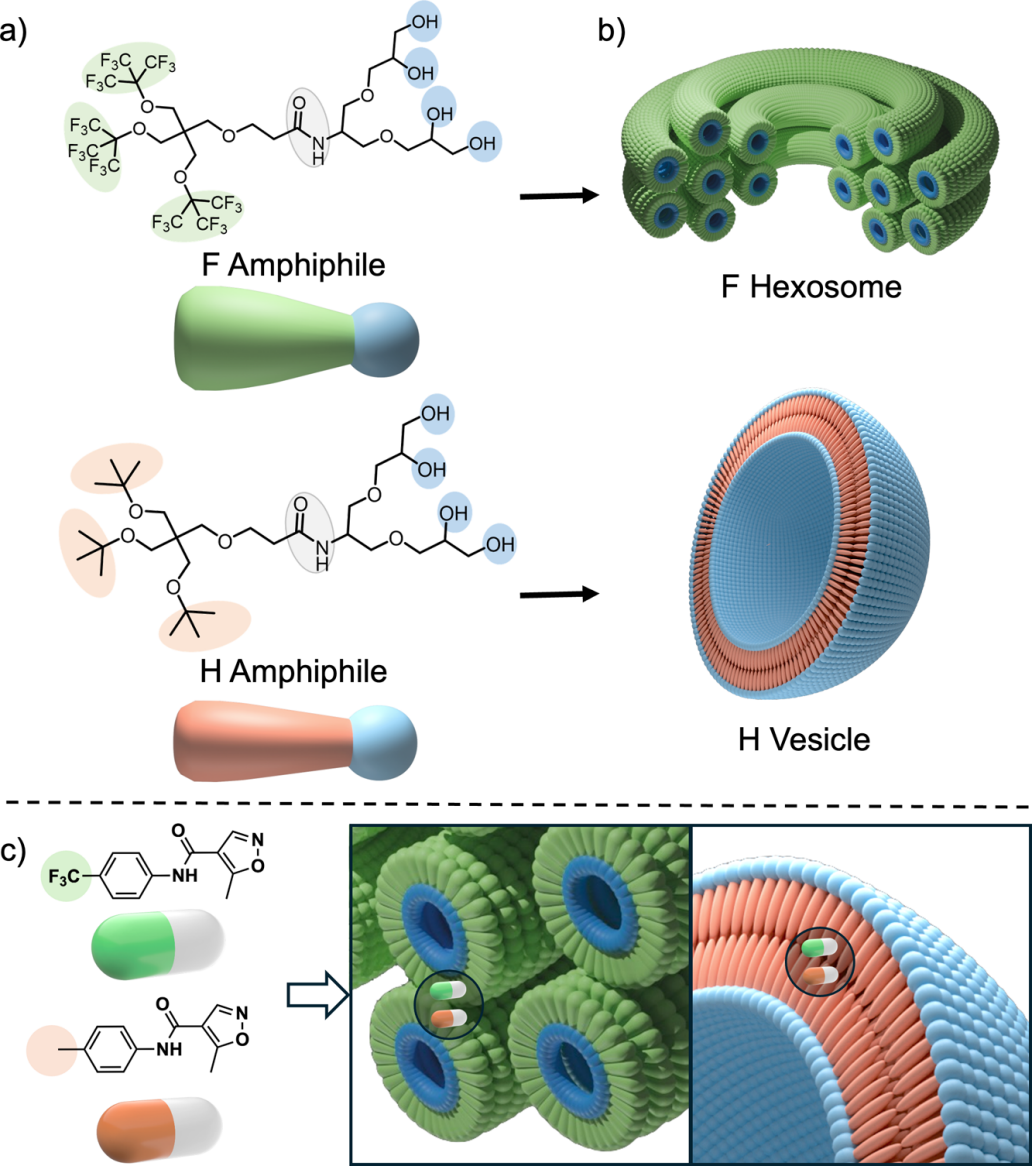
(a) Molecular structures of fluorinated (green) and alkylated (orange) branched amphiphiles; (b) self-assembly of fluorinated amphiphiles into fluorinated hexosomes with the internal structure consisting of hexagonally packed water channels surrounded by an amphiphile monolayer; self-assembly of alkylated amphiphiles into bilayer vesicles; (c) fluorinated drug (LEF, green) and its nonfluorinated equivalent (LEF G, orange) encapsulated within the hexosomes and vesicles.

## Results and Discussion

### Synthesis of Fluorinated and Alkylated Amphiphiles

For drug encapsulation using new amphiphiles, their design and synthesis are of crucial importance. By carefully selecting and modifying molecular components, researchers can tailor amphiphile properties, such as hydrophilicity, hydrophobicity, and supramolecular self-assembly. This enables optimization of drug loading, as well as carrier biocompatibility and stability. Here we have designed and synthesized four amphiphilic molecules that differ in structure, with varying hydrophobic tail groups and linkers that connect the hydrophobic tail to the hydrophilic head.

The synthesis of the fluorinated amphiphiles, BFAG1 and BFTG1 (Scheme 1) was done following published procedures.^36^ The synthesis of the G1 dendron derivatives has been reported previously,^40^ as has the synthesis of the branched fluorinated moiety^41,42^ which is coupled to G1. The first step involves an oxa-Michael addition, which consists of a 1,4-addition of an oxygen nucleophile to a Michael acceptor. The activation of the nucleophile relies on the deprotonation of pentaerythritol by NaOH. A Mitsunobu approach is used with diisopropyl azodicarboxylate and triphenylphosphine as coupling reagents in order to obtain the branched fluorinated groups on all three of the free hydroxyl groups. After this, the molecule is either deprotected to a carboxylic acid using trifluoroacetic acid (TFA) or reduced and then converted to an azide. Either resulting molecule can then undergo coupling reactions with the acetal-protected G1 head (pG1)–clicking either to pG1-alkyne or amide to pG1-NH_2_. Following a final deprotection step using an acid, the final compounds are obtained in good yields (Scheme 1).

**Scheme 1:**
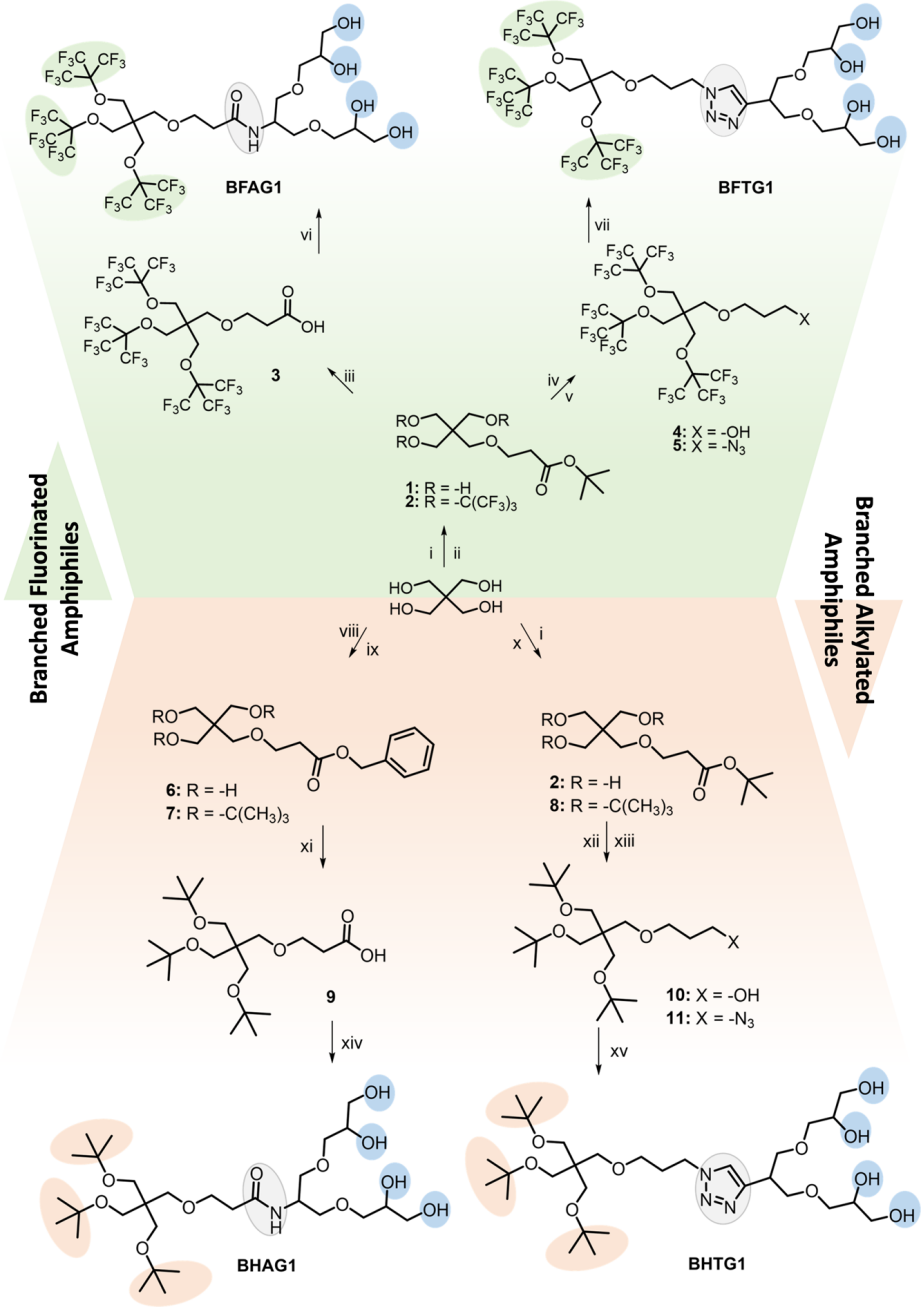
Synthesis of Branched Amphiphiles^a^

a (i) *tert*-Butyl acrylate, NaOH, DMSO, r. t., 12 h, 41%; (ii) Perfluoro-*tert*-butanol, DIAD, DMF, 60 °C, 24 h, 65%; (iii) DCM: TFA (1:1), 91%; iv. LiAlH_4_, THF, r. t., 6 h, 85%; (v) (a) MsCl, TEA, DCM, 0–5 °C, 2 h, 95%; (b) NaN_3_, DMF, 80 °C, 12 h, 87%; (vi) pG1-NH_2_, EDC·HCl, HOBt, DMF, 50 °C, 12 h, Dowex, MeOH, 50 °C, 12 h, 60%; (vii) pG1-yne, Cu­(I)­Ac, DMF, 50 °C, 12 h, Dowex, MeOH, 62%; (viii) NaOH, benzyl acrylate, DMSO, 80 °C, 24 h, 5%; (ix) Ag_2_O, *tert*-butyl iodide, r. t., 3 days, 84%; (x) Ag_2_O, *tert*-butyl bromide, r. t., 3 days, 78%; (xi) H_2_, Pd/C, THF/H_2_O, r. t., 2 days, 98%; (xii) LiAlH_4_, THF, 0 °C, 12 h, 83%; (xiii) DPPA, DBU, DMF, 80 °C, 4 h, 82%; (xiv) NHS, DIC, G1-NH_2_, DIPEA, THF/DMF, 30 °C, 2 days, 14%; (xv) G1-alkyne, Na ascorbate, Cu­(II)­SO_4_·5H_2_O, DMF/H_2_O, 50 °C, 12 h, 88%.

Synthesis of the alkylated molecules, BHAG1 and BHTG1, had to be adapted from the fluorinated procedure, where the Mitsunobu approach is used. Here, the −CH_3_ groups are not electron-withdrawing like the −CF_3_, so Ag_2_O has to be used in combination with the *tert*-butyl bromide or iodide instead of the *tert*-butyl alcohol to form the branched moiety, following the Purdie-Irvine alkylation.^43^ In the case of the triazole-linked molecule BHTG1, the azidation was also reduced to one step for the alkylated synthesis. Otherwise, for BHTG1, the same reaction conditions as those for the fluorinated derivatives could be used to obtain the final amphiphile. Due to the acid sensitivity of the tail group, the azide had to be coupled directly to deprotected G1-alkyne; however, due to the selectivity of the azide-yne click coupling reaction, the yield was comparable to what was obtained for the fluorinated synthesis.

Synthesis of the alkylated amide derivative is made more complicated by the high acid sensitivity of the −OC­(CH_3_)_3_ group. Where normally the *tert*-butyl protecting group would be cleaved using an acid to a carboxylic acid, as in the fluorinated synthesis, here the ether bonds are also broken.^44^ Different conditions and reagents were tried, but a selective cleavage could not be achieved. Instead, a different protecting group was used, namely a benzyl protecting group, which can be selectively deprotected using reduction with H_2_ gas and a Pd/C catalyst. Changing the protecting group of the carboxylic acid decreased the yield of the first step (Scheme 1, viii). The yield of the last step was also lowered, as the amide coupling had to be carried out with already deprotected G1-NH_2_, which lowered the selectivity due to the presence of free hydroxyl groups. Again, these challenges stem from the acid-sensitive branched alkylated tail groups, which are cleaved in the presence of an acid. For these reasons, the synthesis of BHAG1 had an overall yield considerably lower than that of other amphiphiles.

### Characterization of Amphiphile Self-Assembly

#### Determination of Critical Aggregation Concentrations

We determined our four target molecules’ critical aggregation concentration (CAC) in water to get an initial sense of the molecules’ stability.^45^ The CAC results are summarized in Table 1 and Figure S3. Our molecules’ CACs are similar to those of amphiphiles made from G2 hydrophilic heads, where BFAG2 has a CAC of 2.44 × 10^–4^ M and BFTG2 of 0.83 × 10^–4^ M.^36^ Overall, such CACs as were obtained are as expected for nonpolymeric amphiphiles (10^–3^–10^–4^ M).^46^

**Table 1:** CACs of Amphiphiles and Summary of DLS Results in Pure Water with a Concentration of 5 mg mL^–1^

amphiphile	CAC [M]	Z-average (nm)	PDI
BFAG1	2.1 × 10^–4^	154 ± 4	0.17 ± 0.03
BFTG1	1.6 × 10^–4^	157 ± 2	0.16 ± 0.02
BHAG1	2.9 × 10^–3^	215 ± 2	0.44 ± 0.01
BHTG1	1.6 × 10^–3^	212 ± 4	0.54 ± 0.03

#### Formulation of Hexosomes

Preparation methods for amphiphile formulations are important to consider, as they may influence the characteristics of the self-assemblies formed, such as size, polydispersity, and shape. In order to obtain hexosomes, different methods are available, usually involving dispersing the melted amphiphile in water using either a high-energy input (top-down method) or a combination of a hydrotrope like ethanol (EtOH) and a low-energy input (bottom-up method).^9^ Different preparation methods were tested, which are summarized in Table S1; overviews of their characterization by SAXS and cryo-TEM are included in Figures S4 and S5. Our goal was to clarify the self-assembly and form a single type of nonlamellar lyotropic liquid-crystalline nanoparticles, i.e., pure cubosomes or pure hexosomes with a single phase.

The formulation of self-assemblies as seen in Table S1 varied according to the amount of EtOH in the final solutions, the dispersion method of the amphiphile/EtOH mixture in water, and the presence of the stabilizer Pluronic F127 (abbreviated to F127), which is used to stabilize nonlamellar lyotropic liquid crystalline nanoparticles.^9^ Starkly different results were obtained for BFTG1 and BFAG1, despite the only change in structure being the amide or triazole linker. In the latter case, for BFTG1, we could not obtain regular ordering with a single phase or structure. In the case of BFAG1, most methods gave a similar outcome with respect to the peaks found: the main scattering peaks were attributed to the cubic Pn3m phase, but only two peaks were present, potentially due to the presence of other phases, a lack of long-range ordering in the structure, or defects. Better ordering was obtained by dialysis starting from 80% EtOH in water. During the dialysis, the solution went from clear to cloudy, potentially due to increasing light scattering on micron-sized domains. The removal of EtOH was followed by a form of energy input: either long equilibration times of at least a week, heating, or sonication. After this treatment, pure hexosomes were obtained.

As compared to other methods, the one described above that leads to the formation of pure hexosomes is a bottom-up approach. The EtOH used functioned as a hydrotrope, and the water fraction was gradually increased using dialysis.^9^ Indeed, as POM and DSC showed (Supporting Information, Section 3), the melting point of the fluorinated amphiphiles was very high (157 °C, higher than the boiling point of water), so it was difficult to obtain a melt that could be dispersed in water. In the literature, fluorinated polymer cubosomes were formed by controlled addition of water to the polymer dissolved in a solvent,^47^ which is more closely aligned with the method we used for our systems.

#### Average Size and Polydispersity of Self-Assemblies in Solution

DLS was used to evaluate the amphiphile self-assembly in solution (Table 1, Figure S6 and Table S2). This method cannot shed light on the internal structure of the hexosomes, only on the approximate hydrodynamic size of the aggregates. Since the fluorinated and alkylated amphiphiles do not form the same types of self-assemblies, comparing their DLS results is not informative, as any differences are more likely due to variations in shape rather than in actual size. However, we observed a notable increase in the polydispersity index (PDI) from BHAG1 to BHTG1.

DLS was also used to test the stability of the self-assemblies formed from fluorinated amphiphiles (Figure S7), as nonlamellar liquid crystalline nanoparticles are considered thermodynamically stable but not kinetically stable.^48^ They were found to remain stable over a period of 10 days and not to aggregate or fall out of solution, as can be seen by the PDI and Z-average remaining overall constant. Importantly, this does not reveal the internal structure of the self-assemblies but only their average size and dispersity. The precise architecture (cubosome, hexosome, vesicle, or other) is not known from DLS experiments and has to be investigated using other methods, like cryo-TEM or SAXS.

#### Determination and Characterization of Morphologies Formed in Solution as a Function of the Molecular Structure

Cryo-TEM is a key method for studying amphiphile self-assemblies as it provides structural details for each individual assembly, making it especially useful in cases where different morphologies are coexisting. Here, this method enabled us to investigate the self-assembly behavior of the new compounds in their unstressed, native environment (Figure 2).

**Figure 2:**
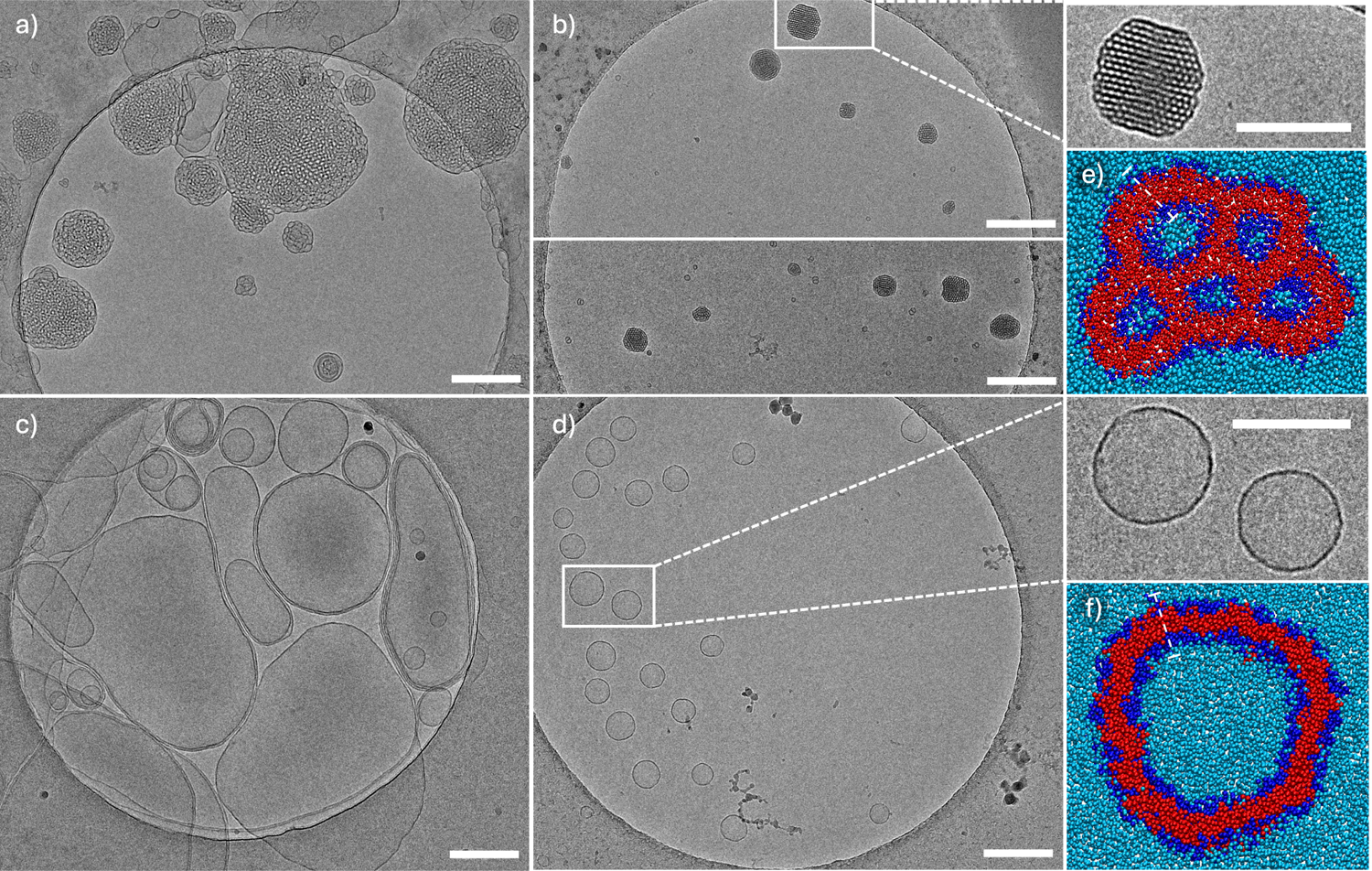
Cryo-TEM micrographs of (a) BFTG1, (b) BFAG1, (c) BHTG1, and (d) BHAG1, Scale bars are 200 nm and concentrations are 5 mg mL^–1^. The insets in (b,d) show the enlarged sections of the respective marked boxes in the cryo-TEM images, scale bars correspond to 100 nm. (e) BFAG1 structure and (f) BHAG1 structure from coarse-grained simulations at concentrations of 20 mM, dark blue indicates hydrophilic segments, red indicates hydrophobic segments, light blue indicates water and white dotted line is the bilayer thickness, which approximates to 3 nm.

Figure 2a shows large, almost globular, self-assemblies with repeating subdivisions for a 5 mg mL^–1^ solution of BFTG1 in water. On closer inspection, hexagonal and cubic patterns can be recognized. Between these self-assemblies, there are also some larger, irregularly shaped, vesicle-like assemblies. Under the same conditions, BFAG1 forms smaller assemblies that have the appearance of solid particles rather than vesicles (Figure 2b). They display a very regular hexagonal segmentation, as can be seen in the crop on the right and in the fast Fourier transform in Figure 3a.

**Figure 3:**
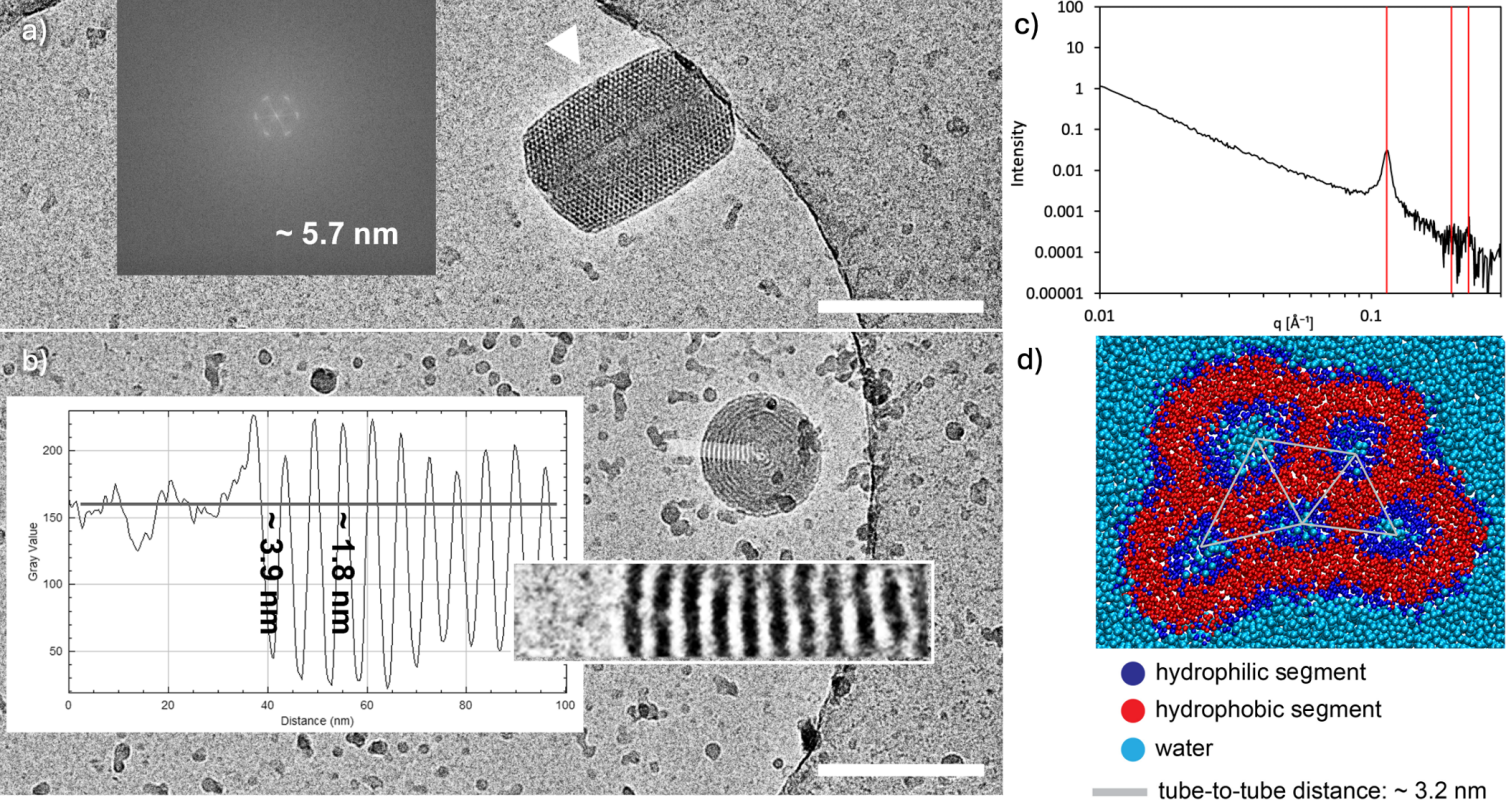
(a) Determination of tube-to-tube distance using fast Fourier transform; (b) BFAG1 self-assemblies and determination of bilayer and water tube thickness; (c) SAXS profiles of BFAG1 hexosomes with locations of expected hexagonal scattering peaks in red; scale bars are 200 nm and concentration 5 mg mL^–1^ for (a)–(c); (d) coarse-grained simulations of BFAG1 (20 mM) with tube-to-tube distance.

As shown in Figure 2c, BHTG1 is found to form multilamellar vesicles of varying sizes and shapes. Some are spherical, but most are large and distorted. There are many multivesicular vesicles; the sizes vary between 100 nm and more than 400 nm. As seen in Figure 2d, BHAG1 also forms vesicles but of markedly different appearance, as they are unilamellar and have a regular circular shape. Indeed, BHAG1 was found to have a PDI lower than that of BHTG1 (see Table 1).

For both fluorinated and alkylated amphiphiles, the linker has an effect on the self-assembly. Triazole and amide bonds are H-bond donors, as well as acceptors. Both contain electrophilic carbon. However, triazole bonds also have a higher dipolar moment, create a larger distance between the different substituents, and take part in π-interactions. Triazole groups are planar like amide groups, but are more rigid.^49^ Further investigation would be necessary to determine which linker property is responsible for the differences in self-assembly between amphiphiles containing different linkers. Here we show that the linker plays a determining role in the self-assembly.

Fluorinated hexosomes are not only a novelty in nonpolymeric supramolecular self-assemblies but also in fluorinated nonlamellar phases. We have therefore investigated the hexosomes’ internal structure in more detail. The central unit of hexosomes is cylindrical water channels, or tubes, which are surrounded by monolayers of the amphiphile, thus forming cylinders. These cylinders have a hydrophobic surface and stack hexagonally to minimize contact with the surrounding water. The complete tube construct is finally surrounded by a lipid-like monolayer. This arrangement is confirmed by hexagonal views such as that shown in Figure 3a, in which the water channels are arranged parallel to the electron beam.

The dimensions of this architecture can be determined directly from the micrographs. On the one hand, the Fast-Fourier-Transform (FFT) of a hexagonal view (Figure 3a) gives the [111] reflexes (inset in Figure 3a) and thus the reciprocal distance (5.7 nm) between neighboring rows of water channels. On the other hand, gray scale plots of small circular self-assemblies (apparently top-views of circular arrangements of the cylinders in perfect alignment with the electron beam) also indicate a distance of 5.7 nm between neighboring rows of tubes (Figure 3b). The value is comparable to other hexosomes from nonpolymeric amphiphiles (4.8–6.2 nm).^50,51^

Measures along the virtual background gray scale value (gray horizontal line in the plot, inset Figure 3b) indicate subdivisions of 1.8 nm (light sections) representing the diameter of the water channels and 3.9 nm (dark sections) representing the amphiphile length. These two-dimensional values are only approximate values, as in three dimensions, the distance between the tubes is slightly (factor $2/\sqrt{3}\approx 1.15$ ) longer than between rows of water channels, and so is the actual diameter and distance. Nevertheless, the measures are perfectly in line with the molecular dimensions of BFAG1, whose length approximates 2 nm in fully stretched conformation.

In contrast to cryo-TEM, SAXS is a bulk technique that provides information about the entire sample, unlike cryo-TEM, which focuses on the local details of individual images. This allows SAXS to capture statistically significant information about the structures present.^52^ For nonlamellar lyotropic liquid crystalline nanoparticles, the scattering peaks typically reveal both the phase and dimensions of the internal structure.

In order to confirm observations made by cryo-TEM, SAXS was used to evaluate ordering within BFAG1 self-assemblies (Figure 3c). The first peak at *q* = 0.1140 Å^–1^ was very sharp and attributed to the first hexagonal scattering peak with an associated Miller index (100). Using Bragg’s law (eq S1), the *d*-spacing was determined to be 5.511 nm, which agrees well with the value found using cryo-TEM. The second and third peaks, with Miller indices (110) and (200) respectively, show the expected pattern for hexosomes (peak spacings of $1:\sqrt{3}:\sqrt{4}$ ). These last two peaks are very small, but as cryo-TEM confirms the presence of hexosomes, these peaks can be attributed to hexagonal scattering. The peak weakness could be linked to the polydispersity of the sample or the presence of defects in the water channel network (i.e., tubes merging with each other in certain places instead of forming straight tubes with hexagonal packing). Other hexosomes in the literature show similar patterns and intensities in their SAXS results.^53^

#### MD Simulations of Solution Self-Assembly Behavior

To gain deeper insights into the self-assembly behavior of amphiphiles, we employed Martini-based coarse-grained MD simulations. We chose to focus on BFAG1 and BHAG1, as these amphiphiles formed better-defined self-assemblies compared to their triazole-containing counterparts. These simulations were conducted at room temperature, starting from randomly inserted amphiphiles in water to closely mirror the experimental conditions. To observe the formation of larger clusters and to clarify the development of water channels within the particles in a feasible computation time, we increased the amphiphile concentration compared to that in experiments. Concentration effects were evaluated at 20, 40, 80, and 160 mM, with no significant changes observed in the overall nanoparticle morphology. For the fluorinated amphiphile, 20 mM corresponds to 21.7 mg mL^–1^, and 160 mM to 174 mg mL^–1^, while for the alkylated amphiphile, 20 mM equates to 12.0 mg mL^–1^.

Figure 2e shows a simulation snapshot of a reversed phase, where the cross-sectional view of the self-assembly reveals water channels surrounded by amphiphilic layers arranged in a hexagonal pattern. This observation is consistent with cryo-TEM images of BFAG1, confirming the presence of an amphiphile bilayer. The step-by-step evolution of hexosome formation is included in Figure S16. This process starts from randomly distributed amphiphiles in water. As depicted, small spherical aggregates initially form a vesicular structure. Upon collision, these vesicles merge, leading to the formation of multitubular aggregates. Over time, the bilayer structures reorganize, ultimately forming a well-defined hexosome structure. Similarly to BFAG1, Figure 2f demonstrates that BHAG1 self-assembles into monolamellar vesicles in the simulations, aligning with the cryo-TEM results.

The simulations indicated a bilayer thickness of approximately 3 nm, which is larger than the 1.8 nm observed in cryo-TEM (Figure 3b), possibly attributed to the coarse-grained nature of the models and the different thickness evaluation methods used in experiments and simulations. Additionally, the average tube-to-tube distance calculated on the snapshot shown in Figure 3d is 3.2 nm, compared to 5.7 nm in cryo-TEM and 5.5 nm in SAXS. We suspected that such a difference is due to the small size of the modeled aggregates, which results in a small sampling for average tube-to-tube distance calculations. To test this, we calculated the average distance for larger self-assemblies obtained from high-concentration simulations and observed that this average approached approximately 5 nm upon increasing the number of tubes, closely matching the experimentally measured value. Overall, the simulations provided valuable insights into the internal morphologies of the self-assemblies, allowing us to examine their internal structure in greater detail, and they were in good agreement with the experimental findings.

### Encapsulation of Fluorinated and Alkylated Drugs

We studied the encapsulation within BFAG1 hexosomes of two different small molecules: Leflunomide (LEF) and Leflunomide Impurity G (LEF G). Both have the same structure, except that the −CF_3_ group in LEF is replaced by a −CH_3_ group in LEF G–, we can thus isolate the role of fluorine. LEF is used as an antirheumatoid agent and shows good antitumor potential, but also suffers from low aqueous solubility.^54^

#### Encapsulation Efficiency and Effect of Fluorine

The encapsulation efficiency was determined for the two different drugs within BFAG1 hexosomes and BHAG1 vesicles (Figure 4). The EE% was determined by HPLC for each of the four drug-carrier pairs (Figure S10, Equation S3). For both carriers, the EE% of LEF was generally higher than that of LEF G, with the disparity being greater in the case of BFAG1. In the case of LEF G, it was encapsulated better in the fluorinated carrier. For LEF, this increase in EE% is much more pronounced, and the encapsulation of the fluorinated drug within the fluorinated carrier was the highest of those of the tested drug-carrier pairs. The EE% was also confirmed using ^19^F NMR using an internal TFA reference of known concentration. NMR showed good agreement with HPLC: 61 ± 5% for LEF in BFAG1 hexosomes and 21 ± 2% for LEF in BHAG1 vesicles.

**Figure 4:**
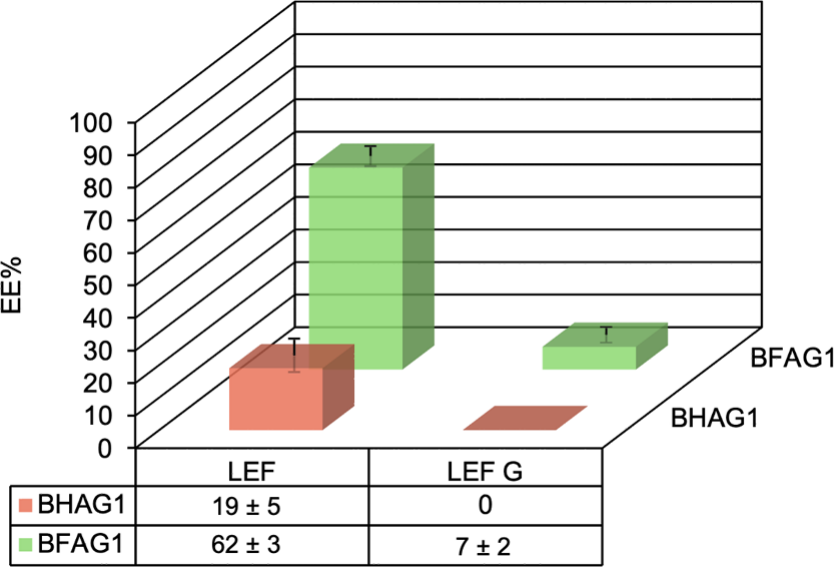
Encapsulation efficiencies for the two drugs and two carriers were determined by HPLC.

The trends we observed using both NMR and HPLC agree with previous studies.^55^ The increased EE% of LEF over LEF G within nonfluorinated self-assemblies (19 ± 5% difference) may be attributed to the higher hydrophobicity of LEF. However, the EE% increase from LEF G to LEF in fluorinated self-assemblies (55 ± 5% difference) is more than twice as large. This finding confirms that the incorporation of fluorine in the molecular structures of both the carrier and the cargo can be used to encapsulate drugs more efficiently, here increasing the solubility of LEF in water by 12-fold.^56^ However, optimization will be needed to reach a higher EE%, as seen in the literature for other hexosome systems.

#### Effect of Drug Encapsulation on the Hexosome Internal Structure

In order to establish the stability of the hexosome carriers upon drug loading, their internal structure was characterized before and after encapsulation. This also reveals the extent of interaction between the drug and the carrier. SAXS measurements were therefore performed for samples without drug loading, loaded with LEF, and loaded with LEF G (Figure 5a–c, black solid lines). The overall shapes of the curves are similar between samples, with a steep increase at low *q*-values and one or two scattering peaks at higher *q*. The data was fitted using the Guinier–Porod model for lower *q*-values. The peaks at high *q*-values are interpreted as a result of a periodic structure inside the particles and are modeled by Lorentzian profiles. Finally, we have a low constant background scattering included in the fitting.

**Figure 5:**
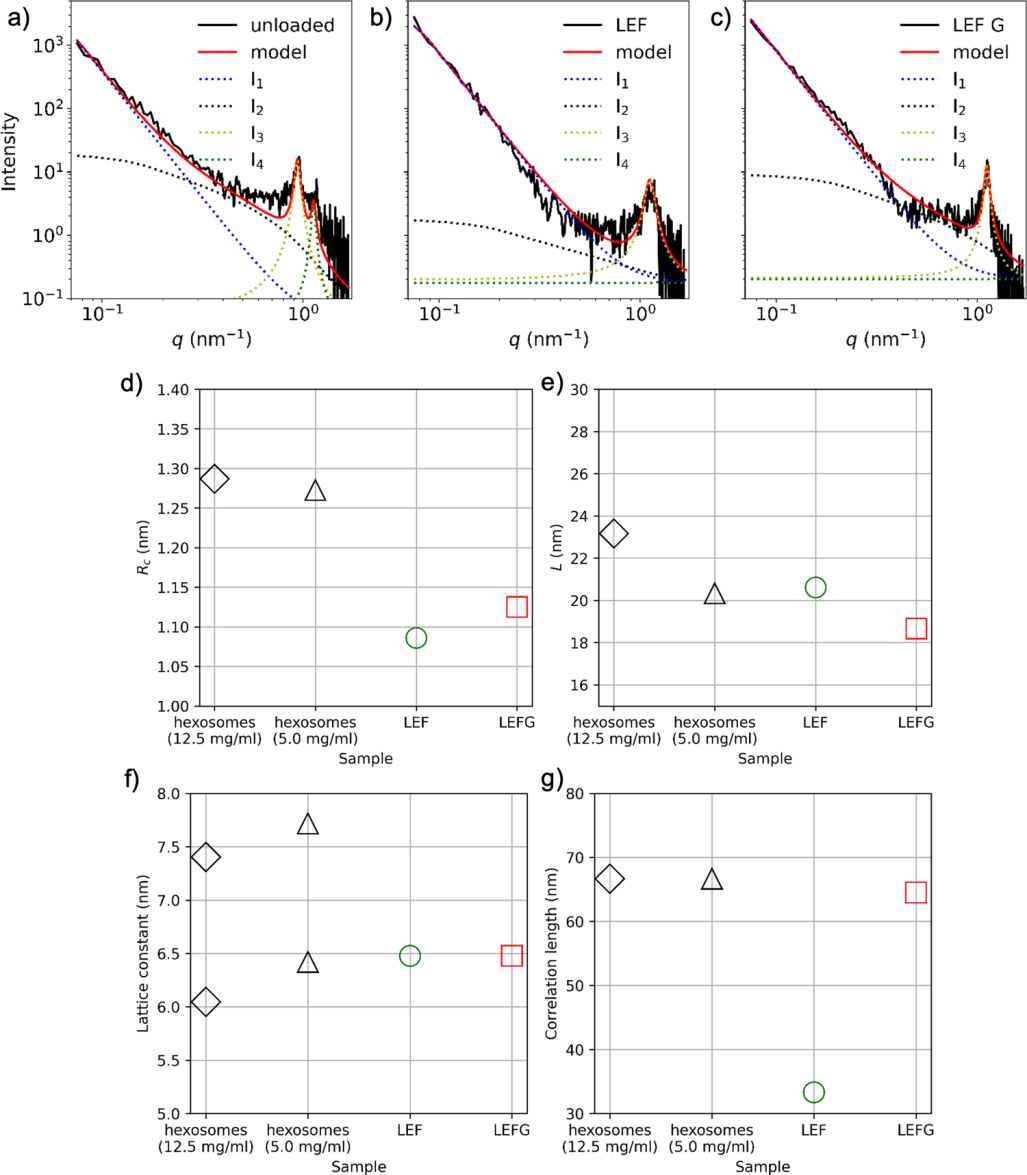
SAXS data from (a) unloaded, (b) LEF, and (c) LEF G samples and model curves (black and red solid lines, respectively). The *I*_1_(*q*), *I*_2_(*q*), *I*_3_(*q*), and *I*_4_(*q*) individual contributions to the overall scattering intensity are provided (blue, black, yellow, and green dotted curves). (d) Radii and (e) length of the cylinder-like internal structures of the hexosomes. (f) Lattice constants and (g) correlation lengths for unloaded samples, LEF and LEF G.

Examples of fitting the data from unloaded, LEF, and LEF G samples with the model are illustrated in Figure 5 (shown as red solid lines). The individual contributions of *I*_1_(*q*) to *I*_4_(*q*) to the overall intensity are also plotted for inspection (dotted lines). Upon examination, the model curves adequately capture the data. The *I*_1_(*q*) provides overall particle radius of gyration values of 20.0 nm (unloaded), 26.6 nm (LEF), and 30.3 nm (LEF G). The diameters of the scatter-equivalent spheres are 51.6 nm (unloaded), 68.6 nm (LEF), and 78.2 nm (LEF G). This indicates that particle size increases due to the incorporation of the active ingredients. The Porod exponent of *d* = 4 for the *I*_1_(*q*) contribution of all samples indicates a narrow interface between the particles and their surroundings.

As derived from *I*_2_(*q*) (Figure 5d,e), the cylinder-like internal structures within the hexosomes have radii of *R*_c_ = 1.27 nm (unloaded), 1.09 nm (LEF), and 1.13 nm (LEF G). Their lengths are *L* = 20.3 (unloaded), 20.6 (LEF), and 18.7 nm (LEF G). When loaded, these cylindrical structures may become slightly thinner, but the cylinder length values remain. Lastly, the unloaded structure appears insensitive to an increase in concentration.

The peak positions and widths obtained from *I*_3_(*q*) provide information about the nanoparticles’ internal lattice type and size (Figure 5f,g). We obtain two lattice constants of *a*_1_ = 6.42 nm and *a*_2_ = 7.72 nm for the unloaded samples and one lattice constant of *a*_1_ = 6.48 nm for LEF and LEF G. The lattice correlation lengths represent the size and perfection of the lattice and were found to be 67 nm (unloaded), 33 nm (LEF), and 65 nm (LEF G). A plausible explanation for the LEF’s low correlation length is that the lattice’s order is significantly disturbed compared to the unloaded and LEF G samples.

From the presence of two lattice constants for the unloaded sample, we can conclude that the structure may not have a hexagonal lattice. Phase-separated nanoparticulate structures other than hexosomes are described in the literature for similar systems, including their thermotropic phase transitions, e.g., from cubosomes to hexosomes.^57^ We therefore performed SAXS measurements as a function of temperature from 21 to 90 °C. The resulting data and the curve fits employing the same model as before are shown in Figure S12. A cubic Pn3m structure is present at room temperature and undergoes a transition to a hexagonal phase at 50 °C (Figure S13). This has been monitored and reported in the past.^58^ Therefore, cubosomes were formed in the unloaded sample, while sonication or equilibration for at least 8 days is needed to form hexosomes after dialysis. Interestingly, an equilibration time of a day was not sufficient here to obtain hexosomes.

#### Insight into Interactions between the Drug and Carrier Using MD Simulations

Coarse-grained MD simulations were conducted to evaluate the interactions between the drug and the carrier. The distribution of LEF and LEF G varied significantly in both fluorinated and nonfluorinated self-assemblies (Figure 6). LEF G was primarily located at the interface between the amphiphile layer and the water phase, whereas LEF was more situated within the amphiphile bilayer, as displayed in Figure 6 and indicated more quantitatively by the radial distribution function of the drug and the hydrophilic portion of the carrier (Figure S18). This distribution pattern may explain the higher encapsulation efficiency (EE%) of LEF compared to LEF G, as LEF, being more deeply embedded within the self-assemblies, leaks out more slowly. Figure S17 further supports this notion by showing that LEF G is more soluble in water and thus is more likely to remain in the external water phase.

**Figure 6:**
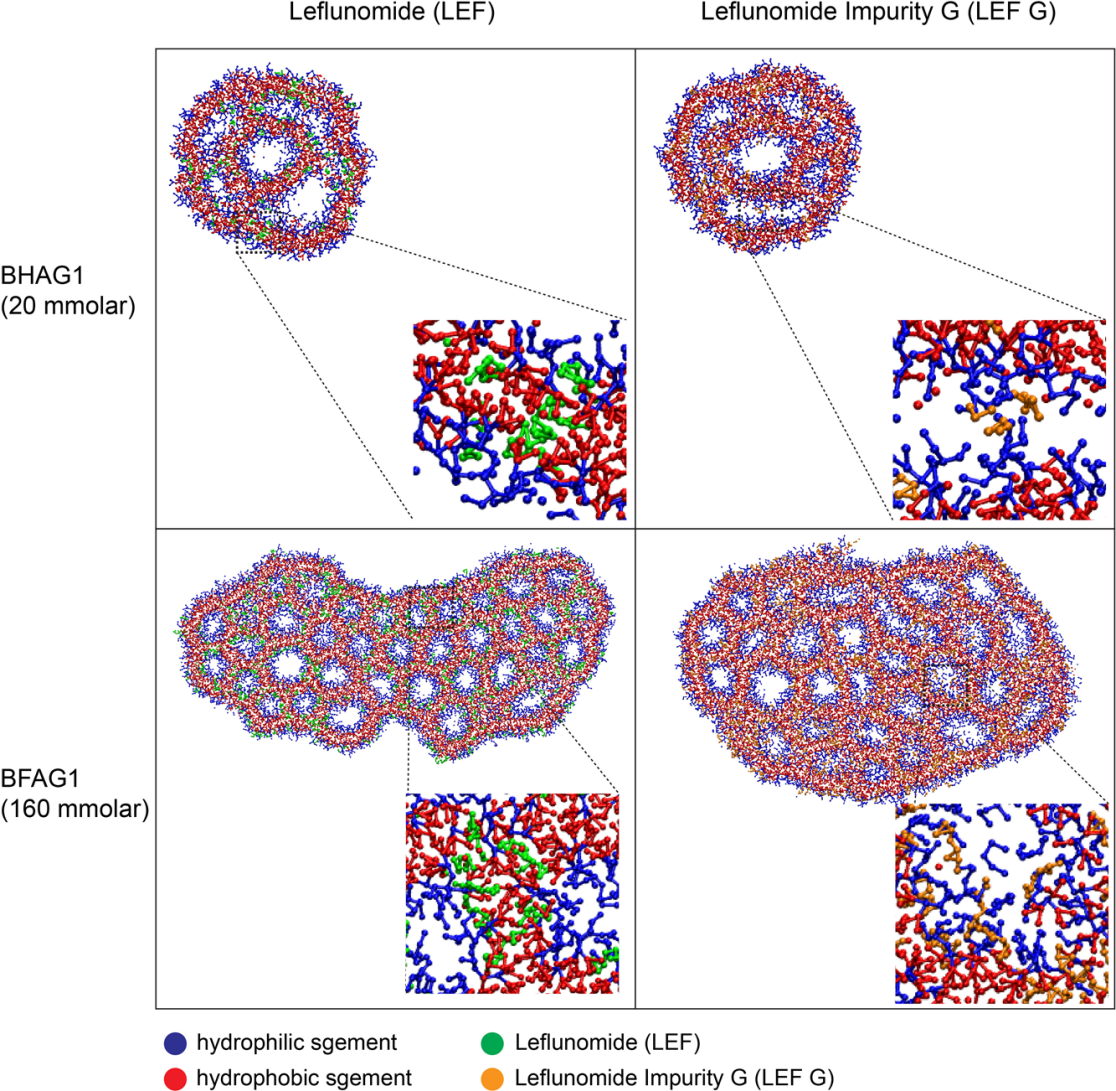
Coarse-grained simulation of the self-assemblies (respective amphiphile concentration on figure) encapsulating the two drugs (10 wt %), showing their distribution, with the fluorinated drug residing inside of the bilayers while the nonfluorinated drug remains at the interface between amphiphile and water phase.

While the simulated encapsulation process differs markedly from experimental conditions, precluding a direct quantitative comparison, we can still gain insights by simulating the encapsulation efficiency of LEF and LEF G. For example, more than 99.8% of LEF is retained within the nanocarrier throughout the 7.5 μs simulation, regardless of the drug and amphiphile concentration, whereas this retention dropped to around 90% for LEF G after 3 μs and then remained constant, for the simulation at 20 mmolar amphiphile concentration. We observed that this value further decreases upon lowering the amphiphile concentration (and accordingly the drug concentration). Moreover, despite clear structural differences between the drugs and amphiphiles, no significant difference was observed between the two types of carriers with respect to their interactions with the drugs.

## Conclusions

In summary, we successfully synthesized four novel double-branched amphiphiles, which allowed us to systematically assess the impact of structural variations on self-assembly, leading to the discovery of fluorinated hexosomes. A new synthetic route was developed to achieve the equivalent alkylated and fluorinated branched molecular structures, thus offering further flexibility to tailor amphiphilic properties by selecting between these moieties. Using SAXS and cryo-TEM enabled the precise determination of self-assembly structures on the nanometer scale despite the intrinsic softness and dynamic behavior of aqueous supramolecular systems. This approach allowed us to characterize hexosomes, vesicles, and mixed phases. Given the potential of hexosomes as nanocarriers, we conducted drug encapsulation studies with fluorinated and nonfluorinated drugs (LEF and LEF G, respectively) as a proof of concept. Both drugs were encapsulated within fluorinated and nonfluorinated carriers (hexosomes and vesicles, respectively), and the encapsulation efficiency was the highest for the fluorinated drug within fluorinated self-assemblies, demonstrating the role of fluorine in improving encapsulation efficiency. The solubility of the poorly water-soluble drug LEF was thus increased 12-fold. Encapsulation mechanisms were further elucidated using SAXS and simulations, which showed that LEF disrupts the lattice ordering of hexosomes and integrates into the hydrophobic layer of the fluorinated self-assemblies. In contrast, LEF G had no effect on the lattice ordering of these assemblies and was found at the interface between the bilayer and water in the simulations. Our studies have shown that fluorinated nonlamellar liquid crystalline nanoparticle systems can be formed and offer significant potential for the encapsulation of fluorinated drugs to increase their solubility in water.

## Experimental Section

### General Materials and Methods

Millipore water was used for the preparation of samples for their self-assembly characterization and encapsulation. Dialysis systems Spectra/Por Float-A-Lyzer (100–500 Da) were used for dialysis. F127 was purchased from Sigma-Aldrich, LEF was from TCI, and LEF G was from Veeprho Pharmaceuticals. Synthetic procedures are reported in Section 2 of the Supporting Information, and corresponding NMR spectra can be found in Section 13.

### DLS

DLS measurements were carried out using the Malvern Zetasizer Ultra (Malvern Instruments Limited, U.K.) equipped with a 10 mW He–Ne laser operating at a wavelength of 632.8 nm. The scattered light was detected using the backscattering setting at an angle of 173 ° (NIBS, noninvasive backscatter).

### CAC

To determine the CAC, the light scattering intensity was measured at various concentrations by serial dilution in water at 25 °C. All measurements were done in triplicate and carried out in 10 mm square quartz cuvettes (Hellma Analytics). The light scattering intensity (in kcps) was plotted against the concentration (in M) to determine the CAC, where both axes were scaled logarithmically. The intensity of scattered light is proportional to the scattering particles, so when aggregates begin to form from monomers, a sharp increase proportional to concentration is observed. This point is the CAC. The derived count rate was approximated as a horizontal line before the CAC since scattering is mainly from water and can be approximated to be constant. For other DLS measurements, they were carried out in single-use Brand UV-cuvettes at a constant temperature of 25 °C.

### Preparation Method of Amphiphile Formulations

In the case of alkylated amphiphiles, these were directly dissolved in water (5 mg mL^–1^), sonicated for 10 min at room temperature, and left to equilibrate overnight. In the case of fluorinated amphiphiles, these were dissolved in EtOH/H_2_O (4:1, 5 mg mL^–1^) with F127 (0.5 mg mL^–1^, 10 wt %). EtOH was gradually replaced by water by dialysis over 24 h, during which the water outside of the dialysis tube was replaced three times at regular intervals. To obtain hexosomes from BFAG1, the resulting solution was either sonicated, heated, or left to equilibrate at room temperature for at least 8 days.

### Cryo-TEM

The samples for cryogenic transmission electron microscopy (cryo-TEM) were prepared in a vitrobot Mark IV (ThermoFisher Scientific Inc., Waltham, MA, USA). The chamber’s climate was set to 22 °C and 100% humidity. After placing a droplet (4 μL) of the sample solution on a hydrophilized (60 s Plasma treatment at 8 W using a BALTEC MED 020 device) perforated Quantifoil grid (Quantifoil Micro Tools GmbH, Großlöbichau, Germany), excess fluid is automatically blotted off to create an ultrathin layer (typical thickness of 100 nm) of the solution spanning the holes of the film. The grids are immediately propelled into liquid ethane at its freezing point (−184 °C). This ultrafast cooling of the aqueous solution (vitrification) is necessary to produce artifact-free cryo-samples by avoiding crystallization of the solvent or rearrangement of the assemblies. The vitrified grids were assembled into so-called autogrids, which can be stored under liquid nitrogen before use. Autrogrids were then transferred into a Talos Arctica transmission electron microscope (ThermoFisher Scientific Inc., Waltham, MA, USA) using the microscope’s autoloader transfer routine. The microscope is illuminated by an X-FEG cathode operated at 200 kV acceleration voltage. Micrographs were recorded on a Falcon 3CE direct electron detector at full size (4K) at a magnification of 28.000× using the microscope's low-dose protocol. The defocus was chosen to be 4 μm in all cases to create a sufficient phase contrast.

### SAXS

The SAXS profiles of the self-assembly samples were studied with a Xeuss 3.0 UHR SAXS/WAXS system (Xenocs SAS, Grenoble, France) equipped with a GeniX 3D Cu K alpha radiation source (λ ≈ 1.54 Å) and with an in-vacuum-motorized three-axis Q-Xoom Eiger2 R 1 M detector (Dectris Ltd., Switzerland) for 2D SAXS/WAXS data collection. The explored *q* range was 0.0015 Å^–1^ < *q* < 2.419 Å^–1^, where *q* is the absolute value of the scattering vector and is defined as *q* = 4π/λsin2θ/2, and 2θ is the scattering angle. Measurements were performed under vacuum (<0.1 mbar) and at room temperature. Each sample was placed in a sealed quartz capillary of *d* = 1.5 mm. The distance of the detector was 300 mm, and the spectrum accumulation time was 1 h at a High-resolution collimation mode. Measurements were also taken using a sample detector distance of 900 mm for 1 h. Measurements were done twice for each detector distance, the data were averaged, and the solvent background (also measured twice and averaged) was subtracted.

SAXS measurements of encapsulation samples were performed in a flow-through capillary with a Kratky-type instrument (SAXSess, Anton Paar, Austria) at a temperature of 20 ± 1 °C for all samples (amphiphile concentration of 5.0 mg mL^–1^) and the unloaded sample with a concentration of 12.5 mg mL^–1^ in a temperature range of 20 ± 1 to 90 ± 1 °C. The SAXSess has a low sample-to-detector distance of 0.309 m, which is appropriate for investigating samples with low scattering intensity. The available *q*-range of *q*_min_ = 0.074 nm^–1^ to *q*_max_ = 1.70 nm^–1^ corresponds to structure sizes between π/*q*_max_ = 1.8 nm and π/*q*_min_ = 42 nm. Each sample was measured as produced for 600 s (60 measurements of 10 s each) at every temperature. The scattering vector *q* is defined in terms of the scattering angle 2θ and the wavelength λ of the radiation (λ = 0.154 nm): thus, *q* = 4π/λsinθ. Deconvolution (slit length desmearing) of the SAXS curves was performed with SAXS-Quant software (Anton Paar, Austria).

We assume that the scattering intensity is represented by *I*(*q*) in eq 1.

$$I(q)=I_{1}(q)+I_{2}(q)+I_{3}(q)+I_{4}(q)$$ 1

where *I*_1_(*q*) results from the overall structure of the particle, *I*_2_(*q*) is a contribution from the inner cylinder-like structures of the hexosomes, *I*_3_(*q*) is a peak pattern, and *I*_4_(*q*) is a background intensity. We employ the Guinier–Porod model for the first two contributions as it has been proven useful for interpreting the scattering of a wide range of particle structures.^59^ The one-level Guinier–Porod term *I*_1_(*q*) is expressed in eq 2.

$$I_{1}(q)=\{\begin{array}{ll} G\exp(-\frac{q^{2}R_{g}^{2}}{3}) & \mathrm{for}\;q\leq q_{1},\\ \frac{D}{q^{d}} & \mathrm{for}\;q>q_{1} \end{array}$$ 2

This term is used to model the steep increase in intensity at the lowest *q*-values. The Guinier form applies for *q* ≤ *q*_1_, and the Porod form applies for *q* > *q*_1_. The *R*_g_ is the overall radius of gyration of the particle, *d* is the Porod exponent, and *G* and *D* are the Guinier and Porod scale factors, respectively. The Guinier and Porod terms and their slopes must be continuous at a value of *q*_1_, a requirement leading to eqs 3 and 4.

$$q_{1}=\frac{1}{R_{g}}{\left(\frac{3d}{2}\right)}^{1/2}$$ 3

$$D=G\exp\left(-\frac{d}{2}\right){\left(\frac{3d}{2}\right)}^{d/2}\frac{1}{R_{g}^{d}}$$ 4

The scattering from curved cylindrical structures inside the hexosomes is modeled by a two-level Guinier-Porod term of cylinders according to eq 5, 6, and 7.

$$I_{2}(q)=\{\begin{array}{ll} G_{2}\exp(-\frac{q^{2}R_{g_{2}}^{2}}{3}) & \mathrm{for}\;q\leq q_{2},\\ \frac{G_{1}}{q}\exp(-\frac{q^{2}R_{g_{1}}^{2}}{2}) & \mathrm{for}\;q_{2}\leq q\leq q_{1},\\ \frac{D}{q^{d}} & \mathrm{for}\;q\geq q_{1} \end{array}$$ 5

$$q_{2}={\left[\frac{2}{3}R_{g_{2}}^{2}-R_{g_{1}}^{2}\right]}^{-1/2}$$ 6

$$G_{2}=\exp\left[-q_{2}\left(\frac{R_{g_{1}}^{2}}{2}-\frac{R_{g_{2}}^{2}}{3}\right)q_{2}^{-1}\right]$$ 7

Utilizing the radii of gyration *R*_g2_ and *R*_g1_, we determine the cross-section radius of the cylinder as $R_{c}=\sqrt{2}R_{g1}$ , and the cylinder length as $L=\sqrt{12R_{g_{2}}^{2}-6R_{c}^{2}}$ . The diameters of the scatter-equivalent spheres are calculated as $D_{\mathrm{sphere}}=2\sqrt{5/3}R_{g}$ .

The peaks at high *q*-values are interpreted as a result from a periodic structure inside of the particles and are modeled by Lorentzian profiles following eq 8.

$$I_{3}(q)=\sum_{i}\frac{k_{i}\sigma_{i}}{\pi (\sigma_{i}^{2}+(q-q_{\max,i}{)}^{2})}$$ 8

where *q*_max,*i*_ is the peak maximum position and σ_*i*_ is the peak width. Finally, we have a low constant background scattering of *I*_4_(*q*) = *I*_4_.

Magana et al.^60^ provided an example of SAXS from the internal hexagonal lattice of hexosomes, where the lattice constant is calculated as $a_{\mathrm{hex}}=4\pi /(q_{\max}\sqrt{3})$ . The correlation lengths *l*_*c*_ = 2π/σ_*max*_ represent the size and perfection of the lattice.

### Encapsulation and Free Drug Removal

Hexosomes and vesicles were prepared as previously described, and LEF or LEF G were added to the carrier solution with a final drug concentration of 0.5 mg mL^–1^. The samples were then sonicated (30 min, room temperature) as sonication disrupts the membrane bilayers and so allows free drug to diffuse into the self-assemblies. Free LEF/LEF G was removed by dialysis in water over 36 h, during which the water outside the dialysis tube was changed 3 times at regular intervals. The full removal of LEF/LEF G was verified with a control experiment involving the dialysis of only the free drugs under the same conditions (Figure S9). The results were similar for LEF and LEF G, with all free drug removed after 26 h and 3 water changes.

### Drug Quantification

For the control experiments, the amount of drug left inside the dialysis tube after dialysis was determined by UV/vis following previously reported procedures by measuring the absorbance intensity at 258 nm (3 replicates for each sample) using a Spark plate reader from Tecan.^61^ A calibration curve for each drug was used to derive the concentration of LEF or LEF G.

For the encapsulation experiments, the EE% was determined by HPLC by taking 40 μL of sample (drug encapsulated inside of carrier) and adding 160 μL of EtOH to dissolve the self-assemblies and so to release the drug. Then this sample was analyzed by HPLC. The Nexera series HPLC from Shimadzu was used, with a mobile phase of 50/50 MeCN/water, at 22 °C, with a flow rate of 1 mL min^–1^, a Gemini C18 Phase 250 × 4.6 mm column from Phenomenex, and a detection wavelength of 260 nm. The calibration curves were done using various concentrations of either LEF or LEF G in 80% EtOH/H_2_O. Each measurement involved 3 replicates.

To confirm results from HPLC, ^19^F quantitative NMR was done using an internal reference of trifluoroacetic acid in acetonitrile-d_3_ (0.011 M). The encapsulation procedure was repeated, encapsulated drug and carrier were dried, and the water was removed by rotary evaporation and replaced by the same volume of acetonitrile-d_3_ and NMR was measured using the Avance 700 (700 MHz) from Bruker.

### In Vitro Release

Release of LEF from BFAG1 hexosomes was investigated by using the dialysis method (Figure S11). Encapsulated LEF and carrier (equivalent to 300 μg of LEF) were put in dialysis tubes, which were then immersed in PBS (45 mL, pH 7.4 to ensure sink conditions).^62^ The samples were incubated at 37 °C on a shaking plate. At different intervals, aliquots were withdrawn from the LEF and carrier sample and replaced with PBS. The amount of LEF was determined by HPLC, and all measurements were carried out in triplicate.

### Coarse-Grained Molecular Dynamics Simulation

To investigate the self-assembly process of amphiphilic structures via molecular dynamics (MD) simulations, we utilized GROMACS^63^ and performed coarse-grained (CG) simulations using the Martini 3 force field.^64^ Atomistic simulations were conducted using the CHARMM36 force field^65,66^ for mapping bonded parameters to the CG scale. Details of the bead types and nonbonded and bonded interactions are provided in the Supporting Information (SI, Section 11).

To facilitate the formation of sufficiently large amphiphilic self-assemblies within a reasonable computation time, we performed MD simulations at slightly larger concentrations compared to the experiment, i.e., from 20 mM (1600 amphiphiles) to 160 mM (12800 amphiphiles). After creating the simulation box containing amphiphiles and water, energy minimization and a short NVT simulation (100 ps) with a 15 fs time step using a V-rescale thermostat^67^ were performed. The production runs were conducted under the NPT ensemble at 300 K and 1 bar using V-rescale thermostat and Parrinello–Rahman barostat,^68^ with a 15 fs time step for 7.50 μs. For the systems containing drugs, the drug molecules were added to the simulation box at 10 wt % of the amphiphilic molecules, and the same steps were followed. To investigate the shape and interior morphology of the self-assemblies, we conducted visual analyses on the self-assembly models, calculated bilayer thickness, water tube-to-tube distance, and radial distribution function of drug and amphiphile molecules.

## Supplementary Material







## Notes

Additional experimental details, materials, methods, data and NMR spectra (PDF)
Movie of simulated BFAG1 self-assembly (BFAG1) (MPG)
Movie of simulated BFAG1 self-assembly (BHAG1)­(MPG)
