# Supplementary material for: Fluorinated Hexosome Carriers for Enhanced Solubility of Drugs
Source: JACS Au. 2025 Apr 28;5(5):2223–36. doi: 10.1021/jacsau.5c00198 (PMC12117447; doi:10.1021/jacsau.5c00198)
Supplement: Supplementary file 1 [file au5c00198_si_001.pdf]

Supporting Information

Fluorinated hexosome carriers for enhanced solubility of drugs

Tiffany Guitton-Spassky,[†] Boris Schade,[‡] Christian Zoister,[†] Eleonora Veronese,[¶] Marta Rosati,[¶] Francesca Baldelli Bombelli,[¶] Gabriella Cavallo,[¶] Andreas F. Thünemann,[§] Hassan Ghermezcheshme,[|] Hesam Makki,[⊥] Roland R. Netz,[#] Kai Ludwig,[‡] Pierangelo Metrangolo,[*,¶] Abhishek Kumar Singh,[*,†] and Rainer Haag[*,†]

†*Institut für Chemie und Biochemie, Organische Chemie, Freie Universität Berlin, Takustraße 3, 14195 Berlin, Germany*

‡*Institut für Chemie und Biochemie, Forschungszentrum für Elektronenmikroskopie, Freie Universität Berlin, Fabeckstraße 36a, 14195 Berlin, Germany*

¶*Laboratory of Supramolecular and Bio-Nanomaterials (SBNLab), Department of Chemistry, Materials and Chemical Engineering "Giulio Natta", Politecnico di Milano, via Luigi Mancinelli 7, 20131 Milan, Italy*

§*German Federal Institute for Materials Research and Testing (BAM), Unter den Eichen 87, 12205 Berlin, Germany*

|*Department of Polymer and Color Engineering, Amirkabir University of Technology, 424 Hafez Avenue, Tehran 15875-4413, Iran*

⊥*Department of Chemistry and Materials Innovation Factory, University of Liverpool, L69 7ZD Liverpool, U.K.*

#*Department of Physics, Freie Universität Berlin, Arnimallee 14, 14195 Berlin, Germany*

E-mail: pierangelo.metrangolo@polimi.it; abhikmc@zedat.fu-berlin.de; haag@chemie.fu-berlin.de

Table of contents:

1 General Synthetic and Analytical Methods **3**

2 Synthetic Procedure and Analytical Data **3**

2.1 BFAG1 3
2.2 BFTG1 3
2.3 Compound 6 4
2.4 Compound 7 4
2.5 Compound 8 4
2.6 Compound 9 5
2.7 Compound 10 5
2.8 Compound 11 5
2.9 BHAG1 5
2.10 BHTG1 6

3 Bulk Thermal Analysis **7**

3.1 POM 7
3.2 DSC 8

4 CAC Determination **9**

5 Preparation Methods of BFAG1 and Effect on Aggregation **10**

6 DLS **13**

6.1 DLS Intensity Profiles 13
6.2 Stability of BFAG1 and BFTG1 Aggregates 13

7 Additional Cryo-TEM Micrographs **14**

8 Determination of d-Spacing **15**

9 Encapsulation of LEF/LEF G **15**

9.1 Removal of Free LEF/LEF G by Dialysis 15
9.2 HPLC Standard Curves 16
9.3 EE% Determination 16
9.4 *In-vitro* Release 16

10 Thermotropic Behavior of Hexosomes **17**

11 MD Simulations **20**

11.1 Coarse Graining 20
11.2 Validation 23
11.3 Hexosome Formation Process 24
11.4 Encapsulation of Drugs 25
11.5 RDF Analysis 26

12 Cytotoxicity **26**

13 Copies of NMR Spectra **28**

13.1 BFAG1 28
13.2 BFTG1 30
13.3 Compound 6 31
13.4 Compound 7 32
13.5 Compound 8 33
13.6 Compound 9 34
13.7 Compound 10 35
13.8 Compound 11 35
13.9 BHAG1 36
13.10 BHTG1 37

14 References **38**

1 General Synthetic and Analytical Methods

All commercially available compounds were purchased from Sigma-Aldrich, TCI and abcr Chemicals. To monitor the reaction progress, pre-coated thin-layer chromatography (TLC) plate (Merck silica gel 60F254) was used with visualization of the spots on TLC using potassium permanganate solution. For column chromatography, a CombiFlash Rf+ Teledyne Isco system was used with RediSep Silver Normal Phase Silica columns. NMR spectra were measured with the spectrometers ECX 400 (400 MHz) and ECP500 (500 MHz) from JEOL and Avance 500 (500 MHz) and Avance 700 (700 MHz) from Bruker. Mass spectra were measured on a 6210 ESI- TOF and 6230 ESI-TOF from Agilent. Abbreviations used are the following: m, multiplet; s, singlet, d, doublet; t, triplet; q, quartet; quint, quintet; sext, sextet; sept, septet.

2 Synthetic Procedure and Analytical Data

Compound numbers follow Scheme 1 from the main manuscript. **Compounds 1 – 5** were synthesized following already published procedures.[1]

2.1 BFAG1

In a pre-dried flask were dissolved **compound 3** (1.85 g, 2.15 mmol, 1.0 eq.) and pG1-NH_2 (0.760 g, 2.37 mmol, 1.1 eq.) in dry DMF (40 mL) under argon. Then followed the addition of EDC·HCl (0.494 g, 2.58 mmol, 1.2 eq.) and HOBt (0.291 g, 2.15 mmol, 1.0 eq). The reaction mixture was heated at 50 °C overnight and reaction progress was followed using TLC (MeOH/DCM). After completion of the reaction, DMF was removed by rotary evaporation. The residue was extracted with DCM (3 × 50 mL) and washed with water. The combined organic layers were concentrated under reduced pressure and the crude product was purified by column chromatography (EtOAc/CyH). The desired protected product (2.03 g, 1.75 mmol) was obtained. The deprotection was carried out with Dowex 50W X8 (H) (1.15 g, 50 wt%) in MeOH (25 mL) at 50 °C overnight. The progress was checked by TLC (MeOH/DCM). After completion of the reaction, the mixture was filtrated and concentrated under reduced pressure. The crude product was purified by column chromatography (MeOH/DCM) to obtain a white viscous solid, **BFAG1** (1.40 g, 1.29 mmol, 60%). ^{1}H NMR (600 MHz, CD_3OD): δ 4.14 (s, 6H), 3.76 (sept, J = 5.4 Hz, 2H), 3.69 (t, J = 6.6 Hz, 2H), 3.59 – 3.43 (m, 15H), 2.48 (t, J = 6.6 Hz, 2H) ppm. ^{13}C NMR (151 MHz, CD_3OD): δ 172.9, 124.5, 122.6, 120.6, 118.7, 81.0, 80.9, 80.6, 73.8, 73.5, 72.2, 72.1, 70.9, 70.8, 68.8, 67.3, 64.3, 64.3, 37.01 ppm. ^{19}F NMR (565 MHz, CD_3OD): δ -71.4 ppm. HRMS (ESI-TOF) m/z: $[M+Na]^+$ calcd. for $C_{29}H_{32}F_{27}NO_{11}Na$ 1106.1442; found 1106.1471.

2.2 BFTG1

In a reaction flask were dissolved **compound 5** (1.20 g, 1.42 mmol, 1.0 eq.) and pG1-alkyne (0.560 g, 1.56 mmol, 1.1 eq.) in dry DMF (20 mL). Then to the solution was added Cu(I)Ac (0.020 g, 0.142 mmol, 0.1 eq.). The reaction has heated up to 50 ºC and the progress was monitored by TLC (MeOH/DCM). After completion of the reaction, the DMF was removed by reduced pressure. The residue was extracted with DCM (3 × 50 mL) and washed with water. Further, the crude product was purified with column chromatography (EtOAc/CyH). The deprotection was carried out with Dowex 50W X8 (H) (50 wt%) in MeOH (25 mL) at 50 °C overnight. The progress was checked by TLC (MeOH/DCM). After completion of the reaction, the mixture was filtrated and concentrated under reduced pressure. The crude

product was purified by column chromatography (MeOH/DCM). The final product **BFTG1** (1.00 g, 0.870 mmol, 62 %) was obtained as a white viscous solid. ^{1}H NMR (600 MHz, CD_3OD): δ 8.00 (s, 1H), 4.79 (s, 2H), 4.47 (t, J = 7.2, 2H), 4.16 (s, 6H), 3.82 (quint, J = 5.0 Hz, 1H), 3.74 (sext, J = 5.0 Hz, 2H), 3.64 – 3.44 (m, 16H), 2.17 (quint, J = 6.6 Hz, 2H) ppm. ^{13}C NMR (151 MHz, CD_3OD): δ 146.5, 125.0, 124.5, 122.6, 120.6, 118.7, 81.3, 81.1, 80.9, 80.7, 78.8, 74.0, 72.4, 72.2, 69.2, 67.0, 64.4, 64.2, 47.5, 31.4 ppm. ^{19}F NMR (565 MHz, CD_3OD): δ -71.4 ppm. HRMS (ESI-TOF) m/z: $[M+Na]^+$ calcd. for $C_{32}H_{36}F_{27}N_3O_{11}Na$: 1174.1816; found 1174.1959.

2.3 Compound 6

In a reaction flask was dissolved pentaerythritol (15.0 g, 0.110 mol, 1.0 eq.) in DMSO (50 mL) and heated up to 80 ºC, then NaOH (0.88 g, 0.022 mol, 0.2 eq.) dissolved in water (2 mL) was added and stirred for 20 min. The addition of benzyl acrylate (21.5 g, 0.132 mol, 1.2 eq.) followed dropwise and the reaction was stirred 24 h at 80 ºC. The mixture was cooled to room temperature, extracted with EtOAc (3 × 100 mL) and washed with water (3 × 100 mL). The combined organic fraction was concentrated on a rotary evaporator. The crude product was purified by column chromatography (6% MeOH/DCM) to obtain a colorless oil, **compound 6** (1.64 g, 5.50 mmol, 5%). ^{1}H NMR (500 MHz, CD_3OD): δ 7.38 – 7.29 (m, 5H), 5.14 (s, 2H), 3.70 (t, J = 6.1 Hz, 2H), 3.55 (s, 6H), 3.44 (s, 2H), 2.63 (t, J = 6.1 Hz, 2H) ppm. ^{13}C NMR (126 MHz, CD_3OD): δ 173.5, 137.5, 129.6, 129.2, 71.5, 68.0, 67.3, 63.1, 46.8, 40.4, 35.9 ppm. HRMS (ESI-TOF) m/z: $[M+Na]^+$ calcd. for $C_{15}H_{22}O_6Na$: 321.1314; found 321.1320.

2.4 Compound 7

In dry DCM (20 mL) was dissolved **compound 6** (1.09 g, 3.65 mmol, 1.0 eq.) under argon, followed by the addition of Ag_2O (3.39 g, 14.6 mmol, 4 eq.) and *tert*-butyl iodide (2.69 g, 14.6 mmol, 4.0 eq.) dropwise. The solution was stirred overnight at room temperature and for the next two days, Ag_2O (1.70 g, 7.30 mmol, 2.0 eq.) and *tert*-butyl iodide (1.25 g, 7.30 mmol, 2.0 eq.) were added each day. After 3 days of reaction time, the mixture was filtered on Celite and solvent and remaining starting material were removed by rotary evaporation, thus yielding a colorless oil, **compound 7** (1.43 g, 3.07 mmol, 84%). ^{1}H NMR (500 MHz, CD_2Cl_2): δ 7.39 – 7.31 (m, 5H), 5.11 (s, 2H), 3.68 (t, J = 6.5 Hz, 2H), 3.35 (s, 2H), 3.26 (s, 1H), 3.22 (s, 5H), 2.59 (t, J = 6.5 Hz, 2H), 1.13 (s, 27H) ppm. ^{13}C NMR (126 MHz, CD_2Cl_2): δ 171.9, 136.8, 128.9, 128.4, 72.2, 70.3, 67.2, 66.4, 60.2, 44.2, 35.7, 31.8, 27.7 ppm. HRMS (ESI-TOF) m/z: $[M+Na]^+$ calcd. for $C_{27}H_{46}O_6Na$: 489.3192; found 489.3223.

2.5 Compound 8

In dry DCM (20 mL) was dissolved **compound 2** (2.0 g, 7.6 mmol, 1.0 eq.) under argon, followed by the addition of Ag_2O (5.8 g, 25 mmol, 3.3 eq.) and *tert*-butyl bromide (2.8 mL, 25 mmol, 3.3 eq.) dropwise. The solution was stirred overnight at room temperature and for the next two days, Ag_2O (2.90 g, 12.5 mmol, 1.7 eq.) and *tert*-butyl bromide (1.50 mL, 12.5 mmol, 1.7 eq.) were added each day. After 3 days of reaction time, the mixture was filtered on Celite and solvent and remaining starting material were removed by rotary evaporation. The crude product was purified by column chromatography (EtOAc/Hex), thus yielding a colorless oil, **compound 8** (2.56 g, 5.9 mmol, 78%). ^{1}H NMR (400 MHz, $(CD_3)_2CO$): δ 4.21 (t, J = 6.3 Hz, 2H), 3.96 (s, 2H), 3.87 (s, 6H), 3.01 (t, J = 6.3 Hz, 2H), 2.05 (s, 9H), 1.74 (s, 27H) ppm.

2.6 Compound 9

In a Schlenk flask was dissolved **compound 7** (1.00 g, 2.14 mmol) in THF and H_2O (9 : 1) under argon, then Pd/C catalyst (100 mg, 10 wt%) was added. The reaction flask was evacuated and put under H_2 gas using a balloon, which was refilled as needed over the course of the reaction, and the mixture was stirred at room temperature for 2 days. The solution was then filtered on Celite, and solvents were removed under reduced pressure to yield a colorless oil, which crystallized overnight to a white solid, **compound 9** (0.79 g, 2.10 mmol, 98%). ^1H NMR (500 MHz, CD_3OD): δ 3.64 (t, J = 6.4 Hz, 2H), 3.36 (s, 2H), 3.27 (s, 6H), 2.50 (t, J = 6.4 Hz, 2H), 1.14 (s, 27H) ppm. ^{13}C NMR (126 MHz, CD_3OD): δ 73.1, 71.0, 68.2, 61.0, 44.9, 36.2, 28.0 ppm. HRMS (ESI-TOF) m/z: $[M+Na]^+$ calcd. for $C_{20}H_{40}O_6Na$: 399.2723; found 399.2766.

2.7 Compound 10

To a THF suspension (30 mL) of $LiAlH_4$ (4.3 g, 10 mmol, 3.3 eq.), **compound 8** (1.3 g, 3.0 mmol, 1.0 eq.) was added at 0°C. The reaction was stirred overnight under nitrogen flow. Then NaOH 0.1 M (4 mL) was added, and the reaction mixture was filtered, and solvent was removed under reduced pressure. The crude product was extracted with EtOAc (3 × 20 mL), washed with brine, dried over Na_2SO_4, and concentrated under reduced pressure to obtain a colorless oil, **compound 10** (0.91 g, 2.5 mmol, 83%). ^1H NMR (400 MHz, CD_3OD): δ 3.65 (t, J = 6.4 Hz, 2H), 3.49 (t, J = 6.2 Hz, 2H), 3.34 s, 2H), 3.27 (s, 6H), 1.77 (quint, J = 6.4 Hz, 2H), 1.15 (s, 27H) ppm.

2.8 Compound 11

In a dry round bottom flask **compound 10** (1.1 g, 3.0 mmol, 1.0 eq.) was dissolved in dry DMF (20 mL) and diphenyl-phophoryl-azide (1.12 mL, 5.20 mmol, 1.7 eq.) was added dropwise to the stirring solution at 0°C followed by 1,8-diazabicyclo(5.4.0)undec-7-ene (0.78 mL, 5.2 mmol, 1.7 eq.). The reaction was left to heat back to room temperature and transferred in a pre-heated oil bath at 80°C then was stirred for 4 h and finally quenched by adding cold water (20 mL). The reaction mixture was concentrated under reduced pressure and extracted with EtOAc (3 × 10 mL). The organic layers were washed with brine, dried over Na_2SO_4, and concentrated under reduced pressure. The crude was then purified by flash column chromatography (3→5% EtOAc/Hex) yielding a pale-yellow oil, **compound 11** (950 mg, 2.45 mmol, 82%). TLC: 5% EtOAc/Hex, Rf = 0.2. ^1H NMR (600 MHz, $(CD_3)_2CO$): δ 3.48 – 3.43 (m, 4H), 3.33 (s, 2H), 3.27 (s, 6H), 1.81 (quint, J = 6.4 Hz, 2H), 1.14 (s, 27H) ppm. ^{13}C NMR (151 MHz, $(CD_3)_2CO$): δ 72.4, 70.2, 68.4, 60.4, 49.2, 44.6, 27.9 ppm. HRMS (ESI-TOF) m/z: $[M+Na]^+$ calcd. for $C_{20}H_{41}N_3O_4Na$: 410.2995; found 410.3036. FTIR (neat) cm^{-1}: 2873, 2096, 1361, 1198, 1078.

2.9 BHAG1

In dry THF (20 mL) were dissolved **compound 9** (909 mg, 2.41 mmol, 1.0 eq.), *N*-hydroxysuccinimide (306 mg, 2.66 mmol, 1.1 eq.) and *N,N*'-diisopropylcarbodiimide (335 mg, 2.66 mmol, 1.1 eq.) under argon and the resulting reaction mixture was stirred overnight at room temperature. The next day, G1-NH_2 (525 mg, 2.19 mmol, 0.9 eq.) was dissolved in THF

and DMF (1 : 1, 10 mL) and added along with *N,N*-diisopropylethylamine (312 mg, 2.41 mmol, 1.0 eq.) and the reaction was stirred for 2 days at 30 °C. The reaction mixture was concentrated under reduced pressure and extracted with DCM (3 × 20 mL). The organic layers were washed with brine, dried over Na_2SO_4, and concentrated under reduced pressure. The crude product was purified by RP-HPLC (85% MeOH/H_2O) to yield a white viscous solid, **BHAG1** (204 mg, 0.341 mmol, 14%). ^1H NMR (700 MHz, CD_3OD): δ 3.77 – 3.75 (m, 2H), 3.64 (t, J = 6.5 Hz, 2H), 3.60 – 3.44 (m, 12H), 3.37 (s, 2H), 3.35 (s, 1H), 3.28 (s, 6H), 2.45 (t, J = 6.5 Hz, 2H), 1.15 (s, 27H) ppm. ^{13}C NMR (176 MHz, CD_3OD): δ 173.9, 73.8, 73.6, 73.1, 72.2, 72.2, 71.2, 71.0, 71.0, 70.9, 70.9, 68.8, 64.4, 64.3, 61.2, 50.2, 45.0, 37.9, 28.0 ppm. HRMS (ESI-TOF) m/z: $[M+Na]^+$ calcd. for $C_{29}H_{59}NO_{11}Na$: 620.3986; found 620.3933.

2.10 BHTG1

In a reaction flask were dissolved G1-alkyne (200 mg, 0.718 mmol, 1.0 eq.) and **compound 11** (206 mg, 0.790 mmol, 1.1 eq.) in dry DMF. In two other flasks were dissolved sodium ascorbate (114 mg, 0.574 mmol, 0.8 eq.) and Cu(II)$SO_4.5H_2O$ (72 mg, 0.288 mmol, 0.4 eq.) in water separately (4 mL each). The latter solutions were added dropwise to the DMF mixture prepared before and they were stirred together at 50 °C for 12h. The solvent was removed by reduced pressure and the residue was extracted with DCM (3 × 20 mL) and washed with water (1 × 20 mL). The crude product was purified by RP-HPLC (75% MeOH/H_2O) to obtain a hard white solid, **BHTG1** (419 mg, 0.629 mmol, 88%). ^1H NMR (700 MHz, CD_3OD): δ 8.00 (s, 1H), 4.79 (s, 2H), 4.51 (t, J = 7.1 Hz, 2H), 3.83 – 3.80 (m, 1H), 3.77 – 3.73 (m, 2H), 3.63 – 3.46 (m, 12H), 3.40 (t, J = 5.9 Hz, 2H), 3.35 (s, 2H), 3.29 (s, 6H), 2.14 (quint, J = 5.9 Hz, 2H), 1.16 (s, 27H) ppm. ^{13}C NMR (176 MHz, CD_3OD): δ 146.3, 125.3, 78.8, 74.0, 73.1, 72.4, 72.2, 70.9, 68.5, 64.4, 64.2, 67.0, 45.0, 31.5, 28.0. HRMS (ESI-TOF) m/z: $[M+Na]^+$ calcd. for $C_{32}H_{63}N_3O_{11}Na$: 688.4360; found 688.4364.

3 Bulk Thermal Analysis

3.1 POM

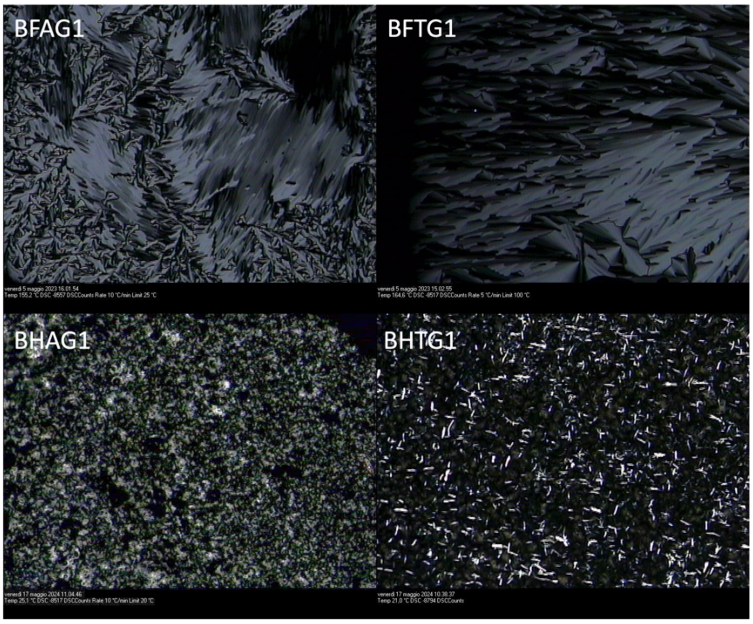

Figure S1: POM micrographs of the 4 amphiphiles upon cooling after reaching clearing point.

3.2 DSC

a)

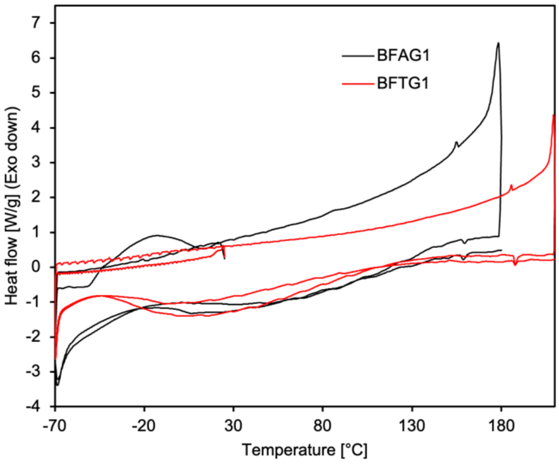

b)

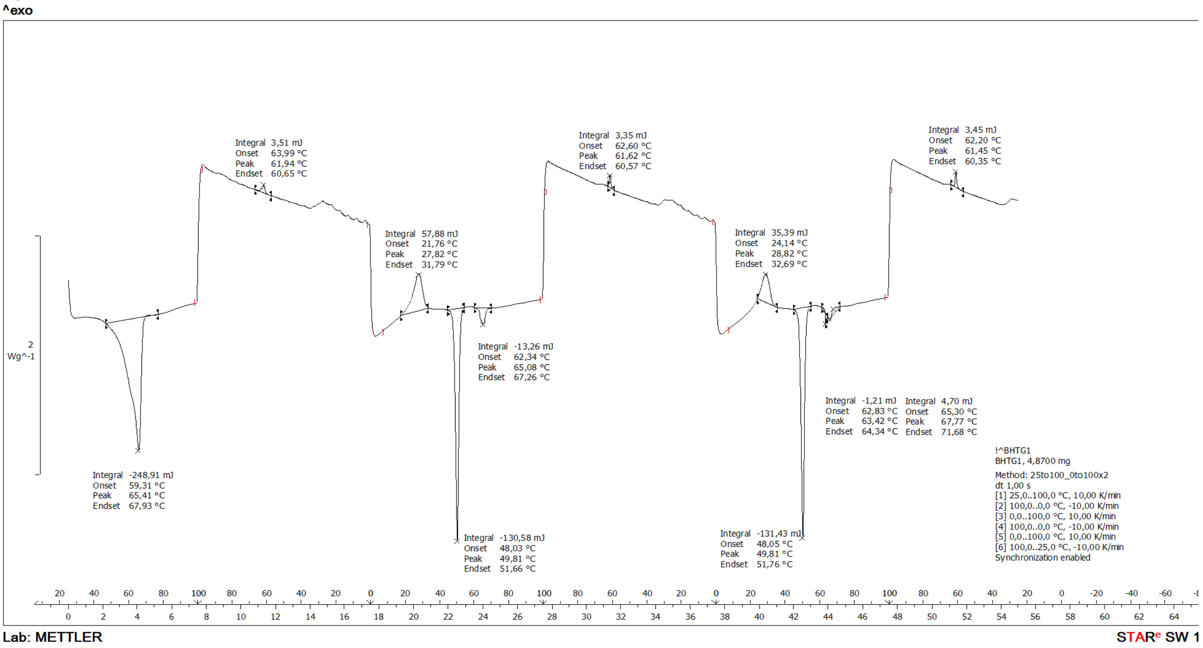

Figure S2 :DSC scan of a) fluorinated and b) BHTG1 amphiphiles.

The fluorinated amphiphiles were viscous liquids at room temperature and showed birefringent features under the POM. Upon heating they reached their clearing point at 187 °C (triazole BFTG1) or 157 °C (amide BFAG1), which were confirmed by DSC. In the case of the triazole alkylated amphiphile BHTG1, it was a solid at room temperature and melted upon heating to form a thermotropic liquid crystal at 66 °C (confirmed using DSC), with a clearing point of 71 °C following soon thereafter (not seen in DSC). The amide alkylated amphiphile BHAG1 was already a liquid crystal at room temperature, and had a clearing point of 42 °C.

4 CAC Determination

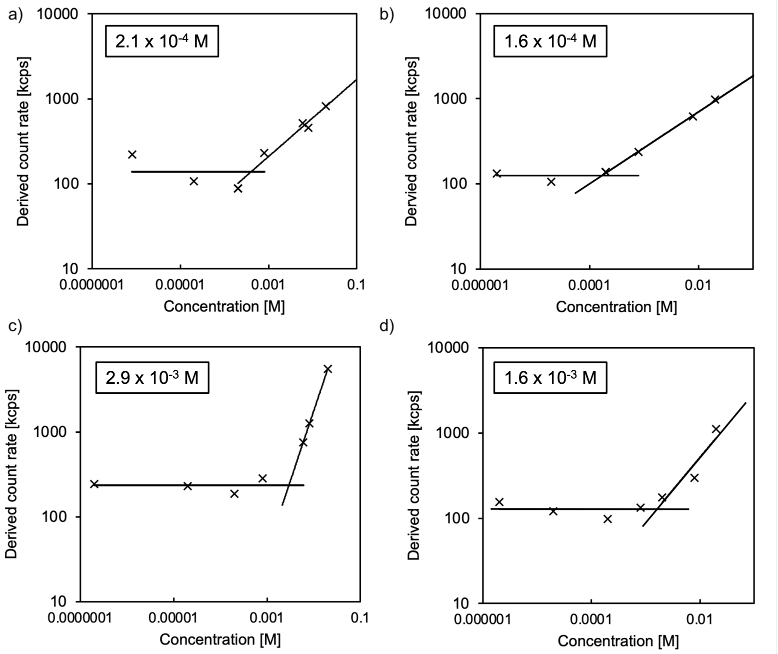

Figure S3: CAC determination of a) BFAG1, b) BFTG1, c) BHAG1, d) BHTG1.

5 Preparation Methods of BFAG1 and Effect on Aggregation

Table S1: Summary of preparation methods and characterization results for BFAG1 and BFTG1.

Compound	10 wt.% F127 (0.5 mg ml^{-1})	Preparation method	Final solution composition	Analysis	Result
BFAG1	No	Dissolved in EtOH (50 mg/ml final conc.), vortexed for 5 min then H_2O added (5 mg/ml final conc.), vortexed for 10 min (**EtOH injection vortex 10 min**)	10% EtOH in H_2O	SAXS and cryo-TEM	Figure S4a, b and c
		Microfluidics mixing between amphiphile solution in EtOH (50 mg/ml) and H_2O (conc. after mixing of 5 mg/ml) (**MF**) with total flow rate (TFR) of 0.4 ml/min	10% EtOH in H_2O	SAXS and cryo-TEM	Figure S5a and b
		MF TFR = 0.6 ml/min	10% EtOH in H_2O	SAXS	Figure S5a
	Yes	MF TFR = 0.1 ml/min	10% EtOH in H_2O	SAXS	Figure S5a
	No	Dissolved in EtOH (50 mg/ml final conc.), vortexed for 1 min then H_2O added (5 mg/ml final conc.), vortexed for 1 min (**EtOH injection vortex 1 min**)	10% EtOH in H_2O	SAXS	No peaks were observed
		EtOH injection vortex 1 min – EtOH removed by rotary evaporation	100% H_2O	SAXS	Figure S5c
	Yes	EtOH injection vortex 1 min	10% EtOH in H_2O	SAXS	Figure S5c
	No	EtOH injection vortex 1 min – heat 2 h at 70 °C	10% EtOH in H_2O	SAXS	Figure S5c
		Dissolve in 10% EtOH in H_2O solution and stir over weekend	10% EtOH in H_2O	SAXS	Figure S5d
		Dissolve in H_2O using sonication (40 min)	100% H_2O	SAXS	Figure S5d
	Yes	Dissolve in H_2O using sonication (20 min)	100% H_2O	SAXS	Figure S5d
	No	EtOH injection vortex 10 min – extrusion through membrane filter 0.45 µm	10% EtOH in H_2O	SAXS	Figure S5e
		EtOH injection vortex 10 min	5% EtOH in H_2O	SAXS	Figure S5e
		EtOH injection vortex 10 min – sonicate 20 min	10% EtOH in H_2O	SAXS	Figure S5e
		80% EtOH in H_2O solution (final concentration 5 mg/ml) and dialyze 24 h to remove EtOH (**dialysis**) – sonication 30 min	100% H_2O	Cryo-TEM	Figure 2b
	Yes	Dialysis – equilibrate over 8 days	100% H_2O	SAXS	Figure 3c
		Dialysis – equilibration 1 day	100% H_2O	SAXS	Figure 5a
		Dialysis – sonication 30 min	100% H_2O	SAXS	Figure 5b and c

BFTG1	No	EtOH injection vortex 10 min	10% EtOH in H_2O	SAXS and cryo-TEM	Figure S4a and d
		EtOH injection vortex 1 min	10% EtOH in H_2O	SAXS	Figure S5f
		EtOH injection vortex 1 min – heat 2 h at 70 °C	10% EtOH in H_2O	SAXS	Figure S5f
		Dissolve in 10% EtOH in H_2O solution and stir over weekend	10% EtOH in H_2O	SAXS	No peaks were observed
	Yes	Dialysis – sonication 30 min	100% H_2O	Cryo-TEM	Figure 2a

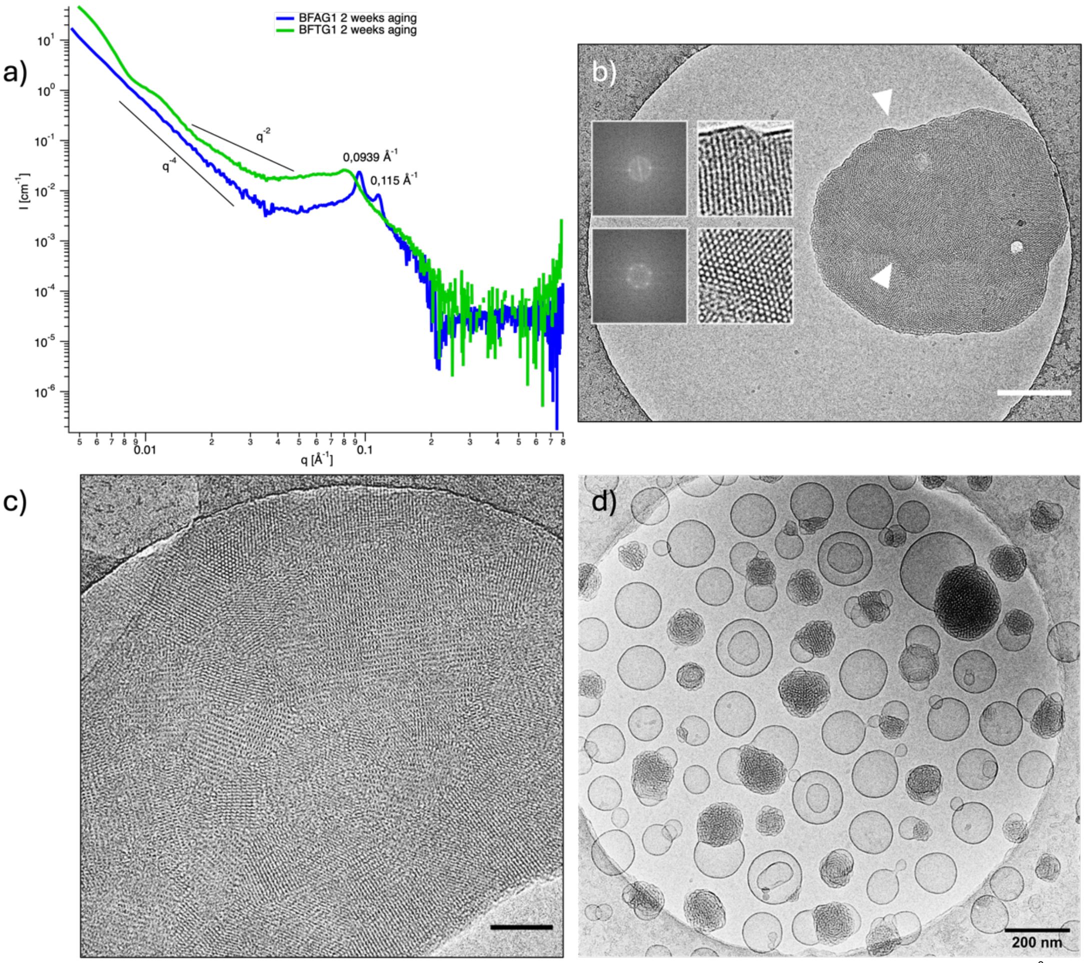

Figure S4: a) SAXS profiles of BFAG1 (1st 2 peaks of Pn3m cubosomes with lattice parameter 96.5 Å) and BFTG1 (mixed bicontinuous phase and vesicles) prepared by EtOH injection followed by 10 min vortexing; b-c) cryo-TEM of BFAG1 sample prepared in the same way; d) cryo-TEM of BFTG1 prepared in the same way.

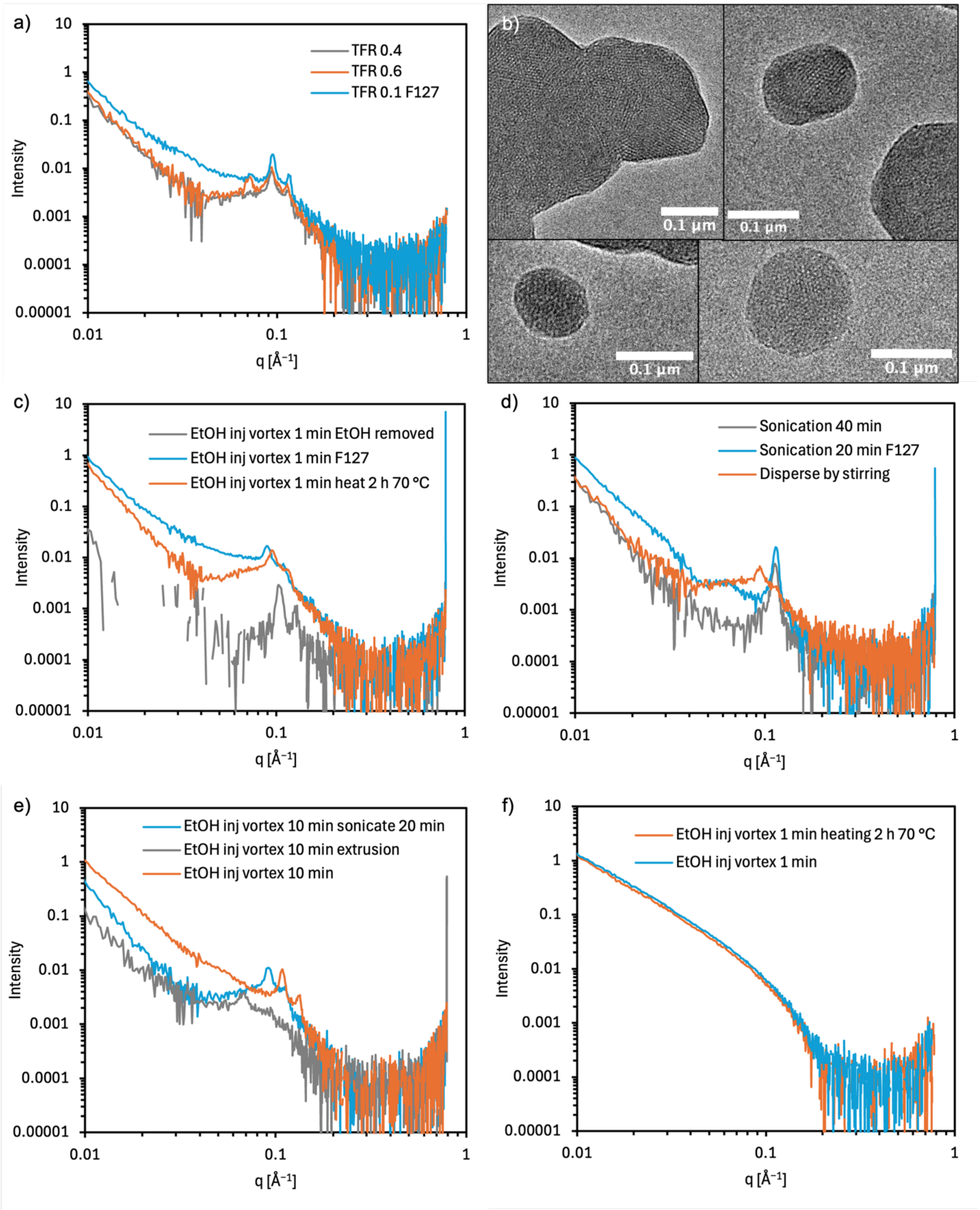

Figure S5: SAXS profiles and cryo-TEM of BFAG1 and BFTG1 prepared using different methods outlined in Table S1.

6 DLS

6.1 DLS Intensity Profiles

a) b)

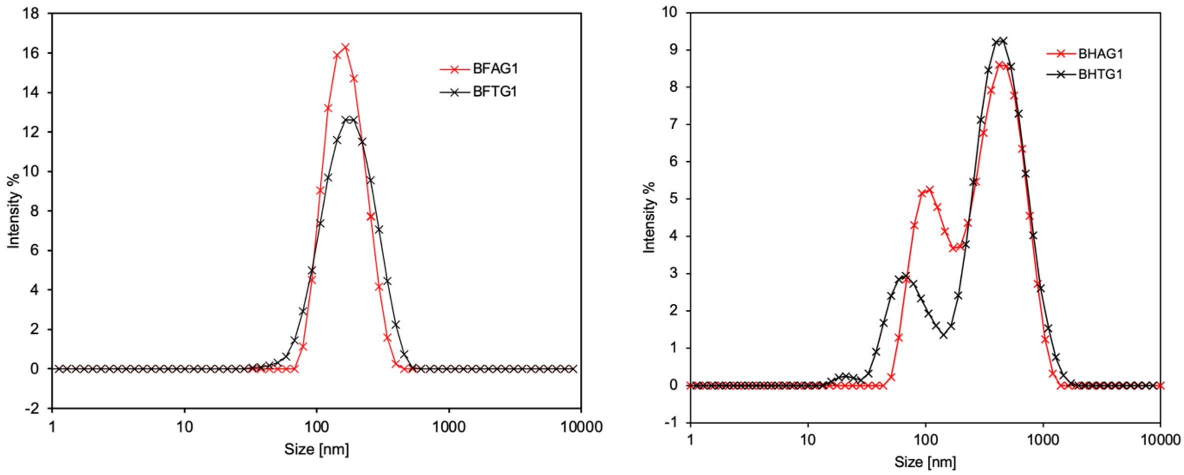

Figure S6: DLS profiles of aggregates formed from a) fluorinated amphiphiles and b) alkylated amphiphles.

Table S2: Summary of peaks from DLS.

Amphiphile	Main intensity peak size [nm] (%)	Second intensity peak size [nm] (%)
BFAG1	171.7 (100%)	
BFTG1	187.4 (100%)	
BHAG1	466.7 (70%)	108.0 (30%)
BHTG1	479.3 (73%)	77.1 (22%)

6.2 Stability of BFAG1 and BFTG1 Aggregates

a) b)

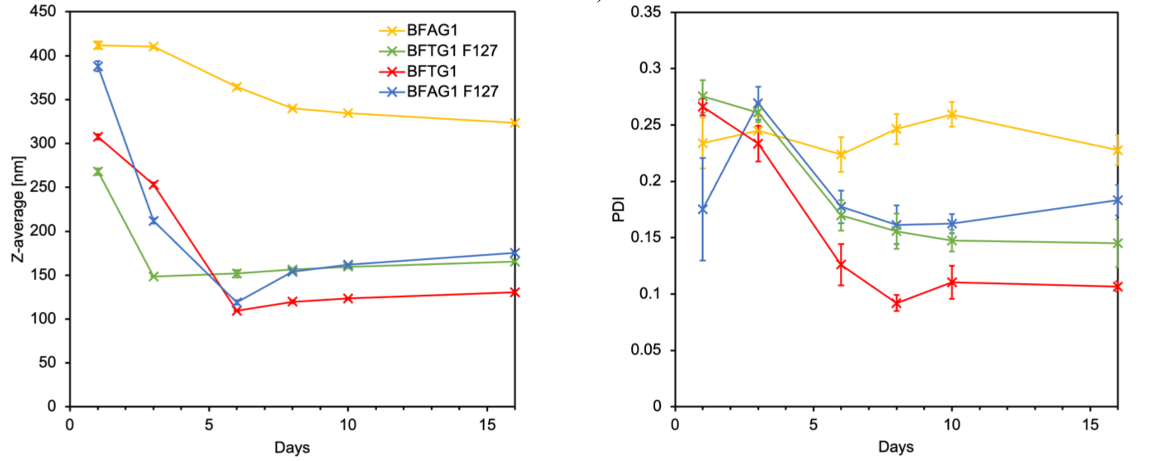

Figure S7: a) Z-average and b) PDI by DLS of aggregates formed from BFAG1 and BFTG1 with and without F127 stabilizer over 16 days, with a sonication step on day 6.

7 Additional Cryo-TEM Micrographs

a) b) c) d)

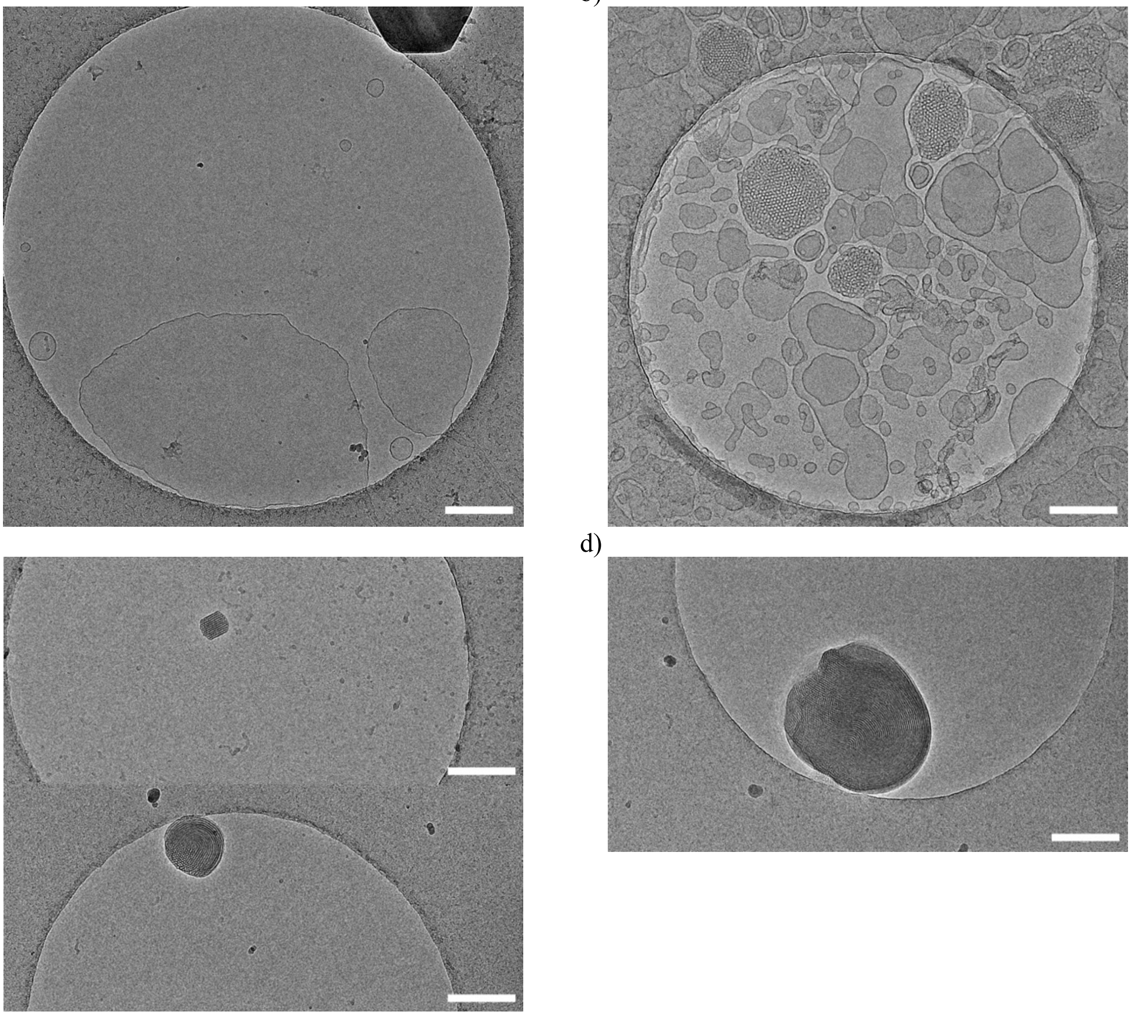

Figure S8: Additional micrographs of aggregates formed from a) BHAG1, b) BFTG1, c-d) BFAG1; in all images the concentration is 5 mg ml^{-1} and the scale bar is 200 nm. The formulation methods are outlined in Table S1.

8 Determination of d-Spacing

Equation S1 is Bragg's law, S2 is the relationship between scattering angle θ and momentum transfer q.

$$d = \frac{n\lambda}{2sin\theta} \tag{S1}$$

$$q = \left(\frac{4\pi}{\lambda}\right) sin(\theta) \tag{S2}$$

Where:

$\lambda = 0.15419nm$
$q = 1.140nm^{-1}$

Thus:

$d = 5.511nm$

9 Encapsulation of LEF/LEF G

9.1 Removal of Free LEF/LEF G by Dialysis

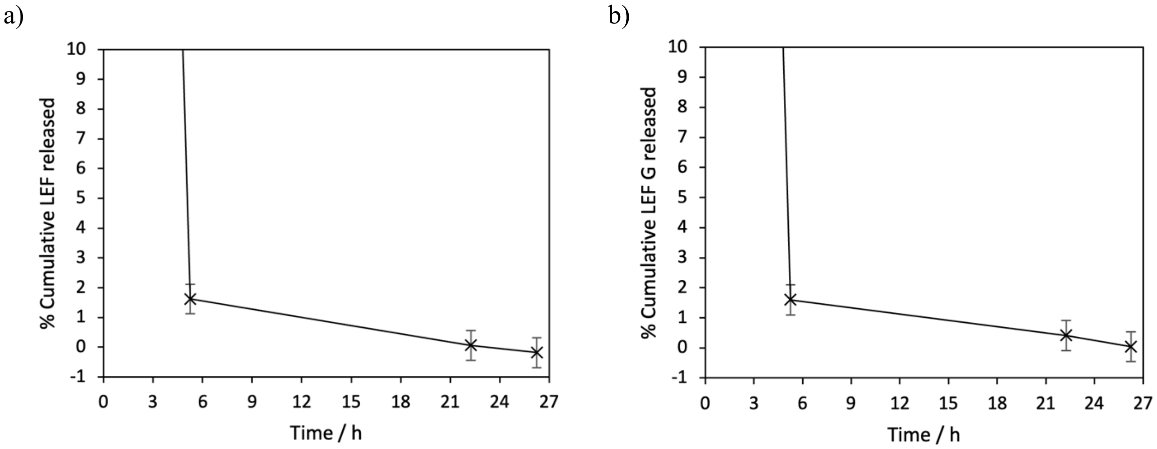

Figure S9: Control experiment for full removal of free drug by monitoring cumulative release of free a) LEF and b) LEF G over time dialyzed in water at room temperature.

9.2 HPLC Standard Curves

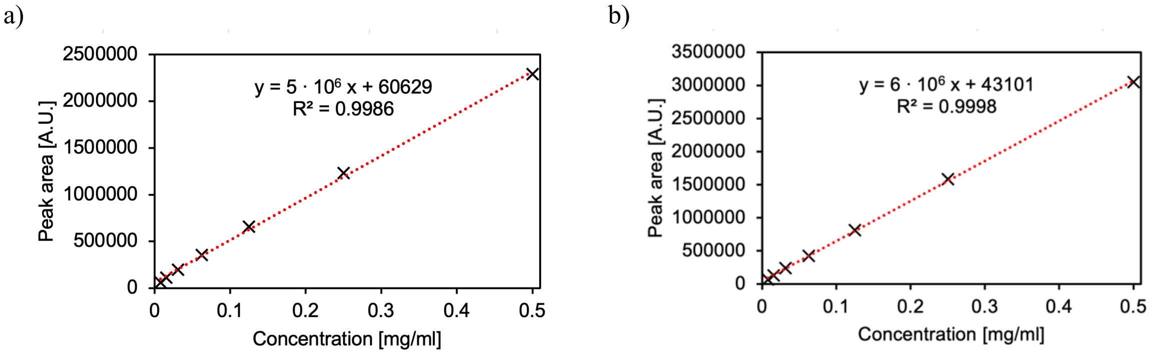

Figure S10: Calibration curves for a) LEF and b) LEF G using peak area at 260 nm.

9.3 EE% Determination

Equation S3 is the encapsulation efficiency (EE%).

$$EE\% = \left(\frac{m_e}{m_i}\right) \times 100 \quad \text{(S3)}$$

Where m_e is mass of either encapsulated LEF or LEF G left after the removal of free drug and m_i is the initial LEF or LEF G mass that was added before the encapsulation procedure.

9.4 *In-vitro* Release

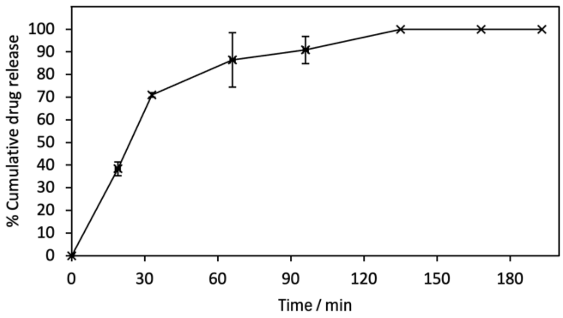

Figure S11: *In-vitro* release of LEF from BFAG1 hexosomes at 37 °C in PBS buffer on a shaking plate.

10 Thermotropic Behavior of Hexosomes

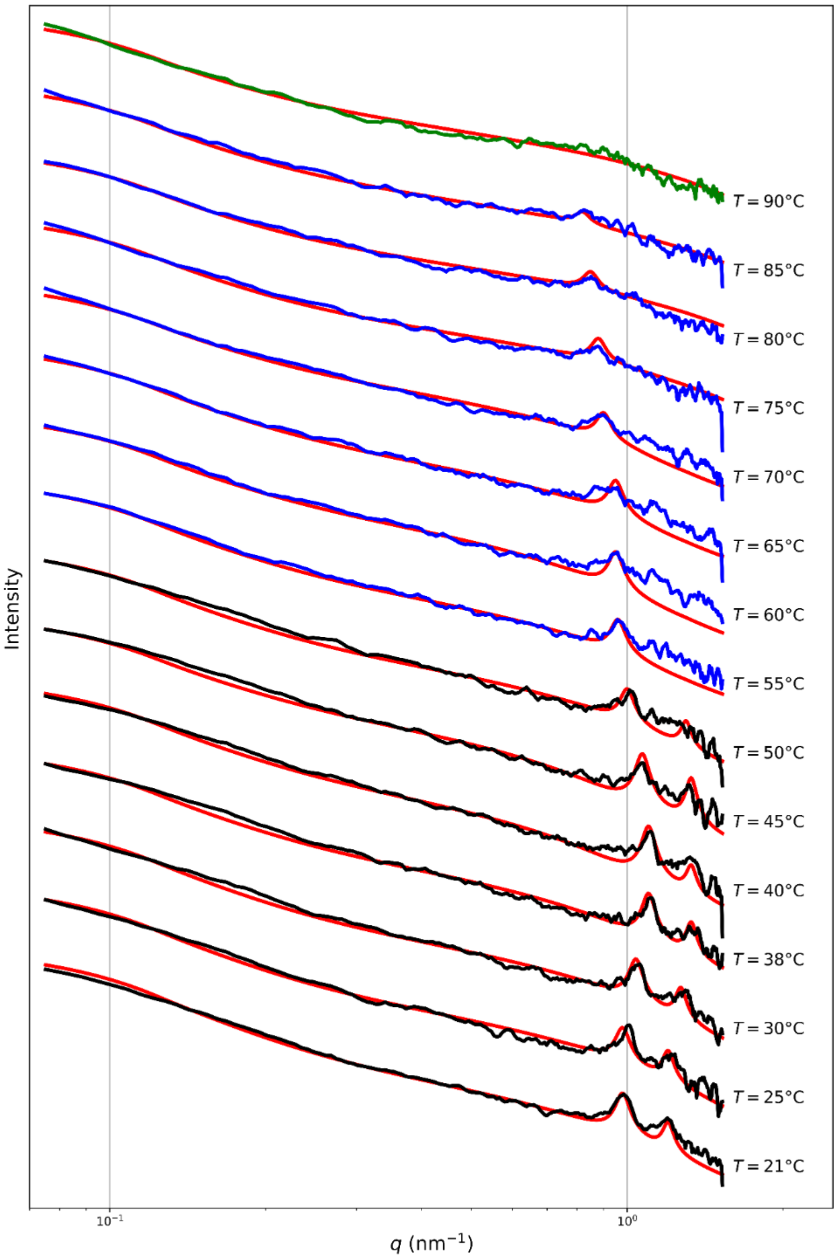

Figure S12: Scattering pattern of an unloaded sample as a function of temperature.

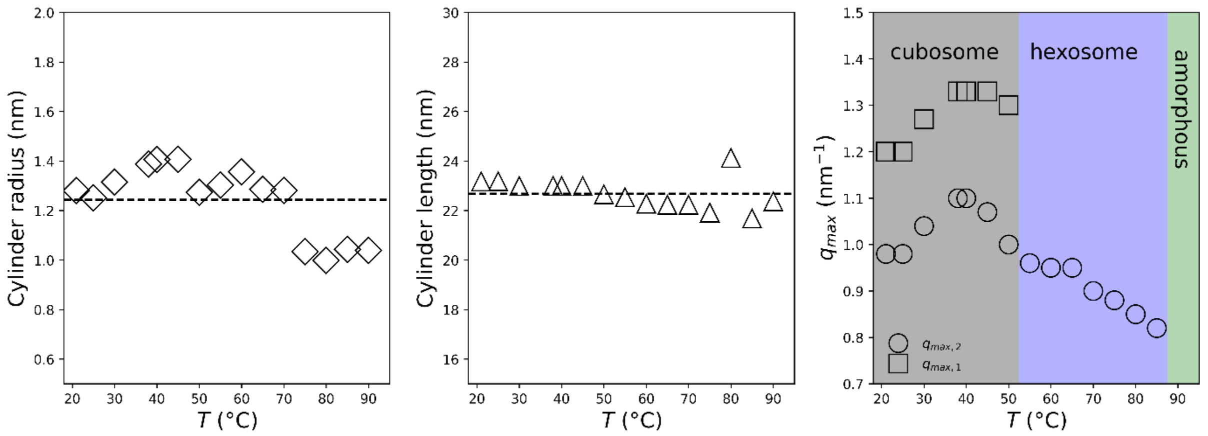

Figure S13: Structure parameters of unloaded samples as a function of temperature. The left figure displays the cylinder radii (diamonds), and the horizontal line is the mean value of R_c = 1.23 ± 0.13 nm. The middle figure shows the cylinder lengths (triangles) and the horizontal line is the mean value at L = 22.7 ± 0.7 nm. The right figure shows the lattice parameters of the cubosomes (temperature range of 21°C to 50°C, gray area), the lattice parameter of the hexosomes (55°C to 85°C, blue area), and the amorphous region (>85°C, green).

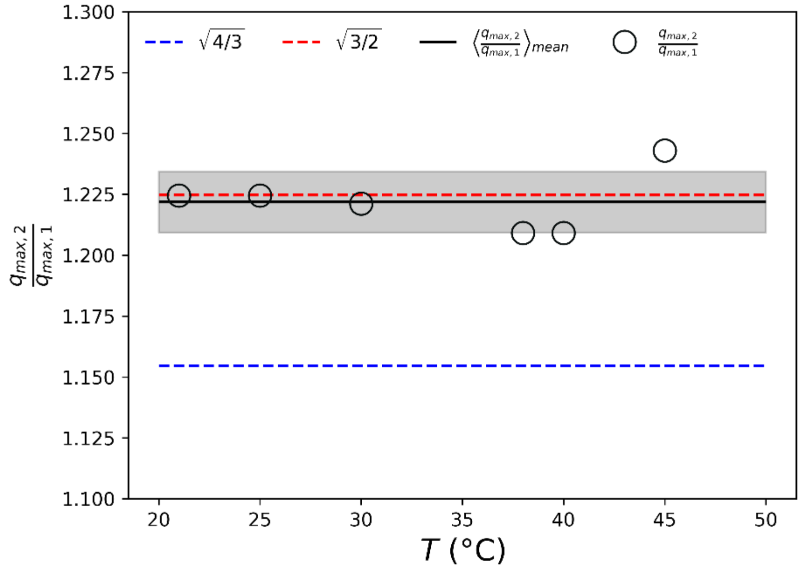

Figure S14: Ratio of peak positions $\frac{q_{max,2}}{q_{max,1}}$ (circles), its mean value and standard deviation is $\frac{q_{max,2}}{q_{max,1}} >= 1.22 \pm 0.01$ (black solid line and gray area).

The overall shape of the scattering pattern is visibly similar for all the data, from which we conclude that the particle shape is consistent throughout this temperature range. However, there are clear differences in the position and number of peaks. For temperatures up to 50 °C, we find two closely consecutive peaks above q =1.0 nm^{-1}. From 55 °C to 85 °C, there is only one peak below q = 1.0 nm^{-1}, which shifts to smaller q-values with increasing temperature. Finally, there is no longer a peak at the highest temperature (90 °C). We may assume that a liquid crystalline phase transition occurs between 50 °C and 55 °C, perhaps a transition from a cubosome to a hexosome structure. Another phase transition occurs between 85 °C and 90 °C, where the ordered internal structure disappears. Here the inner structure likely becomes amorphous. In the following, we quantitatively describe the temperature dependence of the structural changes and discuss the structure parameters as a function of temperature. The overall size of the particles is approximately constant at a diameter of about 52 nm. The cylinder radii are approximately constant at R_c= 1.23 ± 0.13 nm, and the cylinder lengths at L = 22.7 ± 0.7 nm.

Let us now consider the peak positions to estimate the structure of the hypothec cubosomes for which two different phases are possible, i.e. bicontinuous and micellar structures.[4] Similarly, the scattering of cubic phases of membrane lipids is discussed in detail elsewhere.[5] Glatter gives a summary of the peak positions.[6] Cubosomes with a bicontinuous phase structure are known for Pn3m, Im3m, and Ia3d space groups.[4] The sequences of the first two peaks are $\sqrt{2}$: $\sqrt{3}$ (1.00 : 1.22) for Pn3m, $\sqrt{2}$: $\sqrt{4}$ (1.00 : 1.41) for Im3m and $\sqrt{6}$: $\sqrt{8}$ (1.00 : 1.15) for Ia3d. Cubosomes with a micellar structure are known for Fd3m, Pm3n, and Fd3m space groups. The sequence of the first two peaks are $\sqrt{3}$: $\sqrt{8}$ (1.00 : 1.63) for Fd3m, $\sqrt{2}$: $\sqrt{4}$ (1.00 : 1.41) for Pm3n and $\sqrt{2}$: $\sqrt{4}$ (1.00 : 1.15) for Fm3m.

The ratio of $q_{max,2}$ to $q_{max,1}$ is shown in Figure S14. The mean of the ratios is $\frac{q_{max,2}}{q_{max,1}}$ ≥1.22 ± 0.01 (black solid line and grey error band in Figure S14). This value is equal to $\sqrt{3/2} = 1.22$ (red dashed line) and indicative of a Pn3m cubosome structure. Alternatively, the Im3m and Ia3d structures have a reflex ratio of $\sqrt{4/3}$=1.15 (blue dashed line in Figure S14). This is significantly lower than the experimental result, and these two alternative structures can be ruled out. The $q_{max,2}$ to $q_{max,1}$The other possible cubic structures differ even more from the measured ratio and can, therefore, also be ruled out. In conclusion, the cubosomes in the present study have a Pn3m structure. transition. The Pn3m structure is typically a double diamond structure and is known to undergo phase transitions to other mesophases, such as hexagonal or sponge phases, which can be monitored using SAXS.[7] Thermal or pressure stimuli have often induced phase transitions from bicontinuous inverted cubic phases of hydrated monoglyceride lipids to inverted hexagonal (HII) and other nonlamellar phases.[2–5]

11 MD Simulations

11.1 Coarse Graining

We employed charmm-gui (www.charmm-gui.org)[8] to obtain the topology files of the amphiphiles and drugs, based on CHARMM36 force field. The mapping and corresponding Martini 3 bead types assigned to each bead are illustrated in Figure S15.

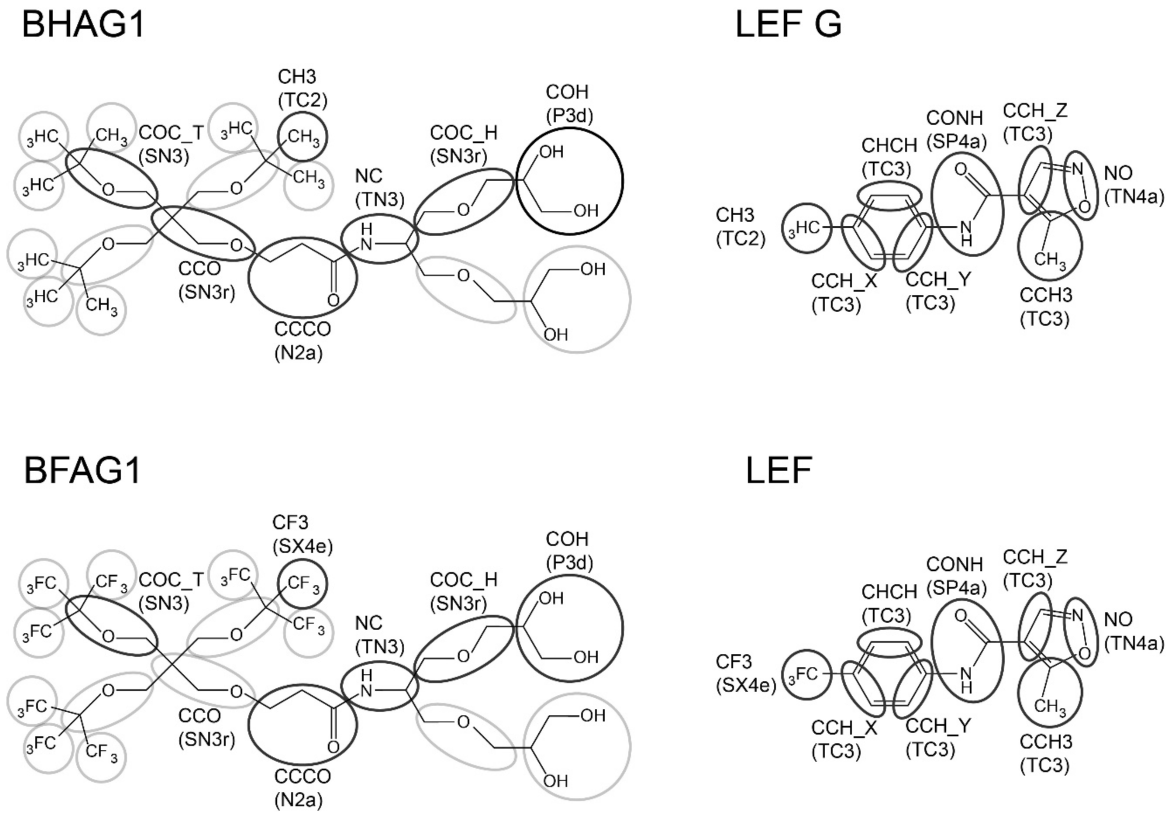

Figure S15: CG mapping of molecules based on Martini 3 standard bead types. The labels show the name of the beads with the corresponding Martini 3 bead type in parentheses.

Mapping from atomistic to the CG level was done based on the standard bead typing of the Martini 3.[9]
The self- and pairwise interactions of CF_3 group (SX4e) with other polar groups (P3d, SN3r, W, SP4a) were parametrized as explained in a previous study.[10] All interactions are listed in Table S3.

Table S3: Self and pairwise non-bonded parameters.

	P3d		SN3r		TN3		N2a		TC2		SN3		TN4a		TC3		SP4a		SX4e		W	
	σ	ε	σ	ε	σ	ε	σ	ε	σ	ε	σ	ε	σ	ε	σ	ε	σ	ε	σ	ε	σ	ε
**P3d**	0.470	3.69	0.430	2.92	0.395	2.31	0.470	3.39	0.399	1.18	0.430	2.92	0.395	2.70	0.399	1.18	0.430	3.77	0.473	1.28	0.465	4.14
**SN3r**			0.410	2.35	0.365	2.11	0.430	3.16	0.366	1.60	0.410	2.60	0.365	2.11	0.366	1.60	0.410	2.35	0.414	1.56	0.425	3.27
**TN3**					0.340	1.77	0.395	2.50	0.352	1.05	0.365	2.11	0.340	1.77	0.352	1.05	0.365	1.91	0.365	2.34	0.385	2.78
**N2a**							0.470	3.24	0.395	1.85	0.430	3.16	0.395	2.31	0.395	2.11	0.430	2.77	0.430	2.92	0.465	3.04
**TC2**									0.340	1.51	0.366	1.60	0.366	0.94	0.340	1.51	0.404	1.13	0.366	1.60	0.395	0.92
**SN3**											0.410	2.60	0.365	2.11	0.366	1.60	0.410	2.35	0.410	2.84	0.425	3.27
**TN4a**													0.340	1.77	0.352	1.05	0.365	1.91	0.365	2.11	0.385	2.51
**TC3**															0.340	1.51	0.404	1.13	0.365	1.91	0.393	1.12
**SP4a**																	0.410	3.31	0.453	1.15	0.425	4.03
**SX4e**																			0.410	2.20	0.514	1.11
**W**																					0.470	4.65

* σ (nm), ε (kJ/mol)

The trajectories from 100 ns of atomistic simulations at 300 K were analyzed to obtain the distribution of distances and angles between the centers of geometry of connected bead atoms. The distributions were converted to the probability (p) and the corresponding potential values were calculated using $U = -ln(p)kT$, in which p is the probability, k is the Boltzmann constant, and T is the temperature in Kelvin. We derived equilibrium values and force constants for bonds and angles through the fitting of harmonic potentials, utilizing Equations S4 and S5, respectively.

$$U_{bond} = \frac{1}{2} K_{bond} (l - l_0)^2 \quad \text{(S4)}$$

$$U_{angle} = \frac{1}{2} K_{angle} \left[cos(\theta) - cos(\theta_0) \right)^2 \quad \text{(S5)}$$

Table S4 and S5 listed the bond and angles constants.

Table S4: Bonds and constraints type parameters at CG level.

**Bond**	***l_0* (nm)**	***K_{bond}* (kJ/mol.nm^2)**
	**BHGA1 and BFGA1**	
COH - COC_H	0.33	6400
COC_H - NC	0.29	5000
NC - CCCO	0.30	21400
CCCO - CCO	0.34	11300
CCO - COC_T	0.30	29100
COC_T - CF3/CH3	0.33	50000
	**LEF and LEF G**	
CCH_Z - CONH	0.23	65000
CONH - CCH_Y	0.27	66000
CCH_X - CF3	0.26	100000
CCH_X - CH3	0.23	78000
CCH3 - NO	0.29	-
NO - CCH_Z	0.19	-
CCH3 - CCH_Z	0.36	-
CCH_Y - CHCH	0.30	-
CCH_Y - CCH_X	0.22	-
CHCH - CCH_X	0.30	-

*In the table, rows without bond constants indicate constraints.

Table S5: Angles type parameters at CG level.

Angle	θ_0 (°)	K_{angle} (kJ/mol.rad^2)
**BHAG1**		
COH-COC_H-NC	116	68
COC_H-NC-COC_H	97	103
COC_H-NC-CCCO	127	201
NC-CCCO-CCO	90	74
CCCO-CCO-COC_T	114	102
COC_T-CCO-COC_T	74	520
CCO-COC_T-CH3	150	133
CF3-COC_T-CH3	70	1000
**BFAG1**		
COC_H_NC_COC_H	97	103
COC_H_NC_CCCO	127	201
NC_CCCO_CCO	90	74
CCCO_CCO_COC_T	114	102
COC_T_CCO_COC_T	74	520
CCO_COC_T_CF3	150	133
CF3_COC_T_CF3	70	1000
CCO_COC_T_CF3	150	133
**LEF and LEF G**		
CCH3-NO-CCH_Z	90.97	2500
CCH3-CCH_Z-CONH	74.27	1150
NO-CCH_Z-CONH	129.19	1500
CCH_Z-CONH-CCH_Y	115.52	550
CONH-CCH_Y-CHCH	77.46	2600
CONH-CCH_Y-CCH_X	145.14	5300
CHCH-CCH_Y-CCH_X	68.23	6400
CHCH-CCH_X-CH3	83.33	1500
CCH_Y-CCH_X-CH3	150.86	5500
CHCH-CCH_X-CF3	81	6500
CCH_Y-CCH_X-CF3	145	6000

11.2 Validation

The validation of non-bonded and bonded interactions at the CG level was assessed by comparing bulk density and radius of gyration of molecules in water. Densities were calculated for 50 fully relaxed molecules at 298 K at both the atomistic and CG levels, and the radius of gyration was calculated in water at a concentration of 20 mM. These verification properties are listed in Table S6. The differences between the values obtained at the all-atom and CG levels indicate that the parametrization is acceptable.

Table S6: Comparison of density and radius of gyration values at all-atom and CG levels.

Amphiphilic or drug molecule	Property	AA	CG	Diff. (%)
**BHAG1**	Density(g/cm^3)	1.048	0.947	-9.6
	Radius of gyration in water (nm)	0.468	0.510	+9.0
**BFAG1**	Density(g/cm^3)	1.606	1.477	-8.0
	Radius of gyration in water (nm)	0.536	0.546	+1.9
**LEF G**	Density(g/cm^3)	1.163	1.092	-6.1
**LEF**	Density(g/cm^3)	1.375	1.400	+1.8

11.3 Hexosome Formation Process

a)

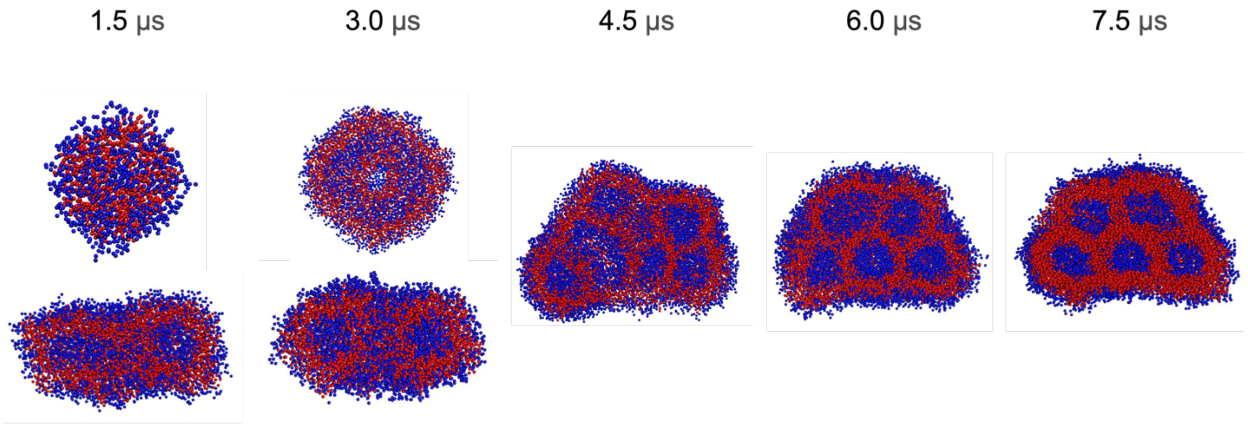

b)

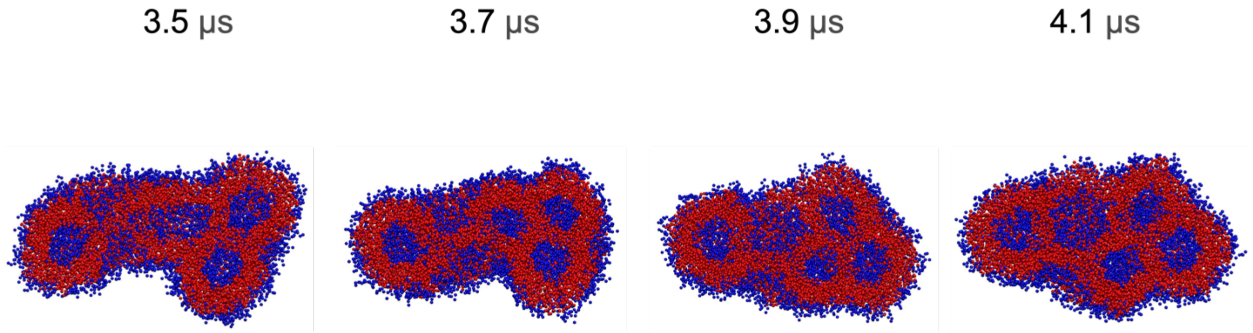

Figure S16: a) Step-by-step evolution of hexosome formation; b) in-between steps between 3 to 4.5 μs, showing how these tubular structure merge and eventually form the final hexosomes.

11.4 Encapsulation of Drugs

The experiments revealed that the encapsulation efficiency (EE%) of LEF was higher than that of LEF G. These results are consistent with the data obtained by HPLC (Figure 4). Figure S17 presents the final snapshots of the simulations for BHAG1 and BFAG1 with LEF and LEF G. In all cases, the solubility of LEF G is higher, as many LEF G molecules are observed floating in water, a phenomenon not seen in the LEF simulations.

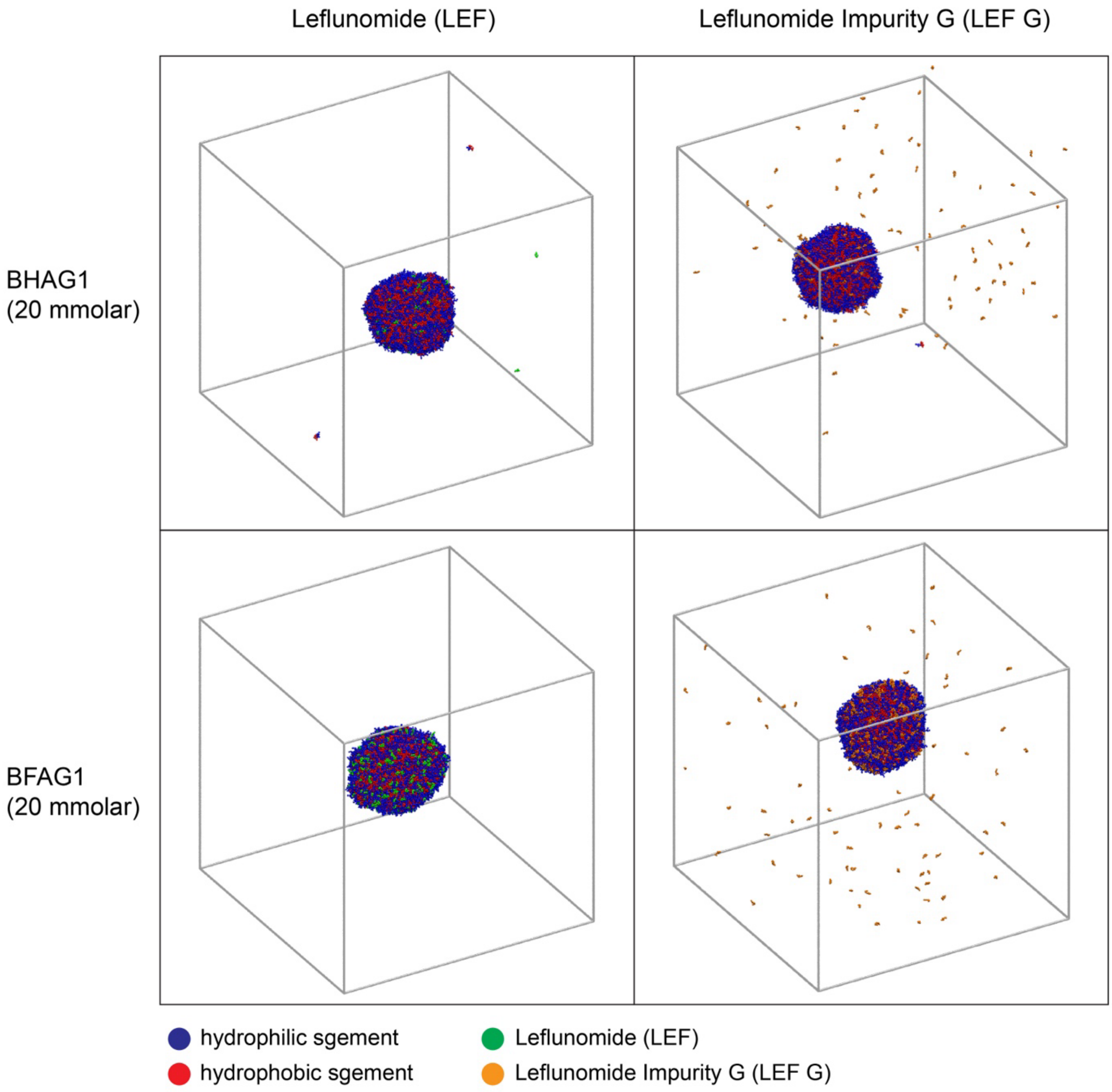

Figure S17: Visual representations of the simulated systems, illustrating the arrangement of drugs within the carrier structures at the end of the simulation.

11.5 RDF Analysis

To quantify the proximity of LEF and LEF G molecules to the hydrophilic segments of the BFAG1 carrier, the radial distribution function (RDF) was employed. As highlighted in Figure S18 (red ellipse), the significant difference in the first peak of the RDF indicates that LEF G has a stronger affinity for the hydrophilic part of the carrier, suggesting a higher potential for the drug to leak out of the carrier.

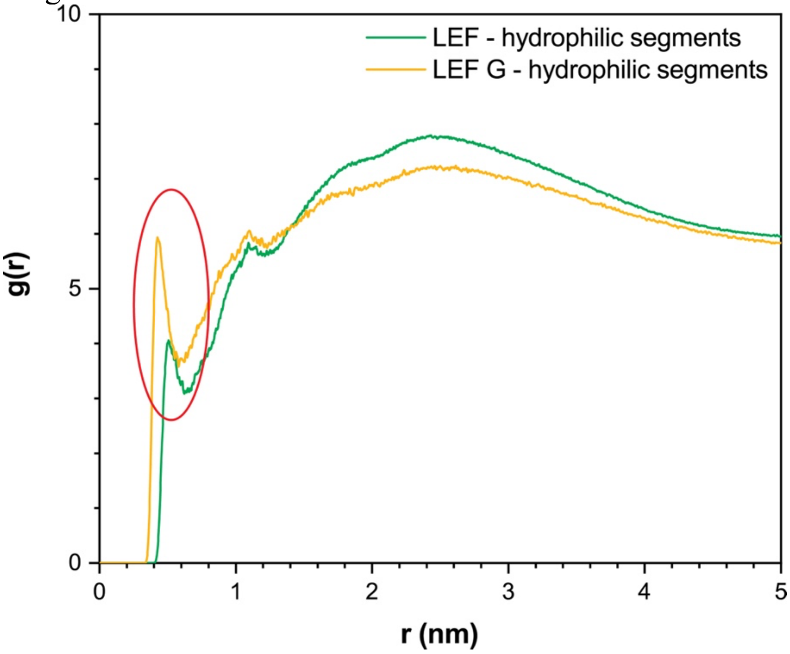

Figure S18: RDF Analysis of LEF and LEF G Drug with hydrophilic segment of BFAG1 Carriers.

12 Cytotoxicity

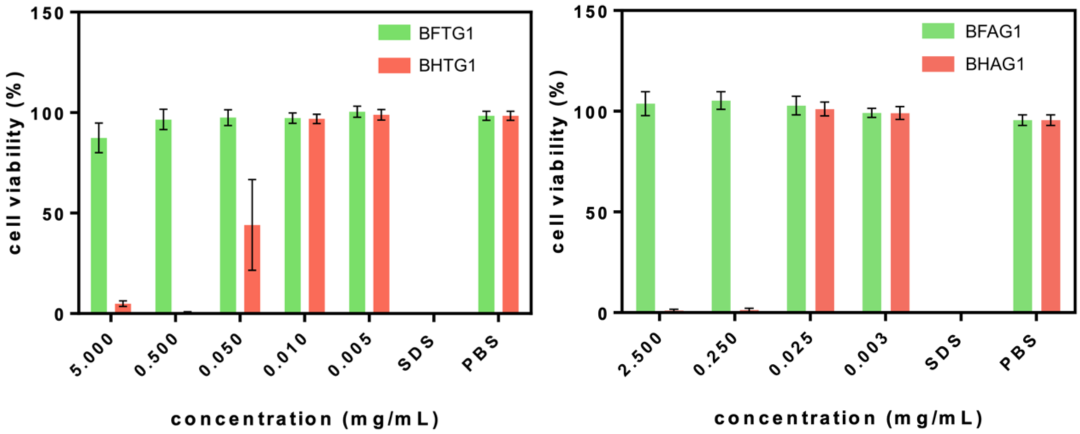

Figure S19: Concentration dependent cell viability of HeLa cells tested by the CCK-8 assay after 24 h of incubation.

The effect of tail fluorination on cellular toxicity was determined by comparing BFAG1 and BHAG1, and we found that fluorination made the amphiphiles much less cytotoxic. The same was found for the equivalent fluorinated G2 structures as was previously published.[1] Even considering the high molecular weight of BFAG1 compared the BHAG1 (1083.53 g mol^{-1} compared to 597.79 g mol^{-1}), the difference in cell viability is still notable. The fluorinated amphiphile was less cytotoxic by a factor of more than 5 compared to the alkylated amphiphile.

The cell viability of amphiphiles were investigated on Hela (ATCC number CCL-2) cells. The cells were cultured in Dulbecco's Modified Eagle's Medium (DMEM) supplemented with 10% FBS, 1% penicillin and streptomycin (all from Gibco BRL, Eggenstein, Germany). The cells were subcultured twice a week when they reached 90% confluency. For the cell viability assay, 90 µL of a cell suspension in DMEM containing 1×10^4 cells ml^{-1} were seeded in each inner well of a 96-well plate and incubated for 3 days at 37 °C and 5% CO_2. In the outer wells, were prepared with 90 µL DMEM without cells. After reaching confluency, 10 µL per concentration of the desired compound was added to the cells in duplicates and in addition to one outer well for the background correction. SDS (1%) and PBS served as controls. The cells together with the compounds were incubated for 24 h (96 h) at 37 °C and 5% CO_2. Afterwards the CCK-8 solution was added (10 µL per well), and the absorbance at a measurement wavelength of 450 nm and a reference wavelength of 650 nm was measured after 3 h incubation using a Tecan plate reader. Measurements were performed in duplicates and repeated three times. The cell viability was calculated by normalizing to the non-treated control after correction by the background using the Excel software. The graphs were plotted in GraphPad Prim 6.

13 Copies of NMR Spectra

13.1 BFAG1

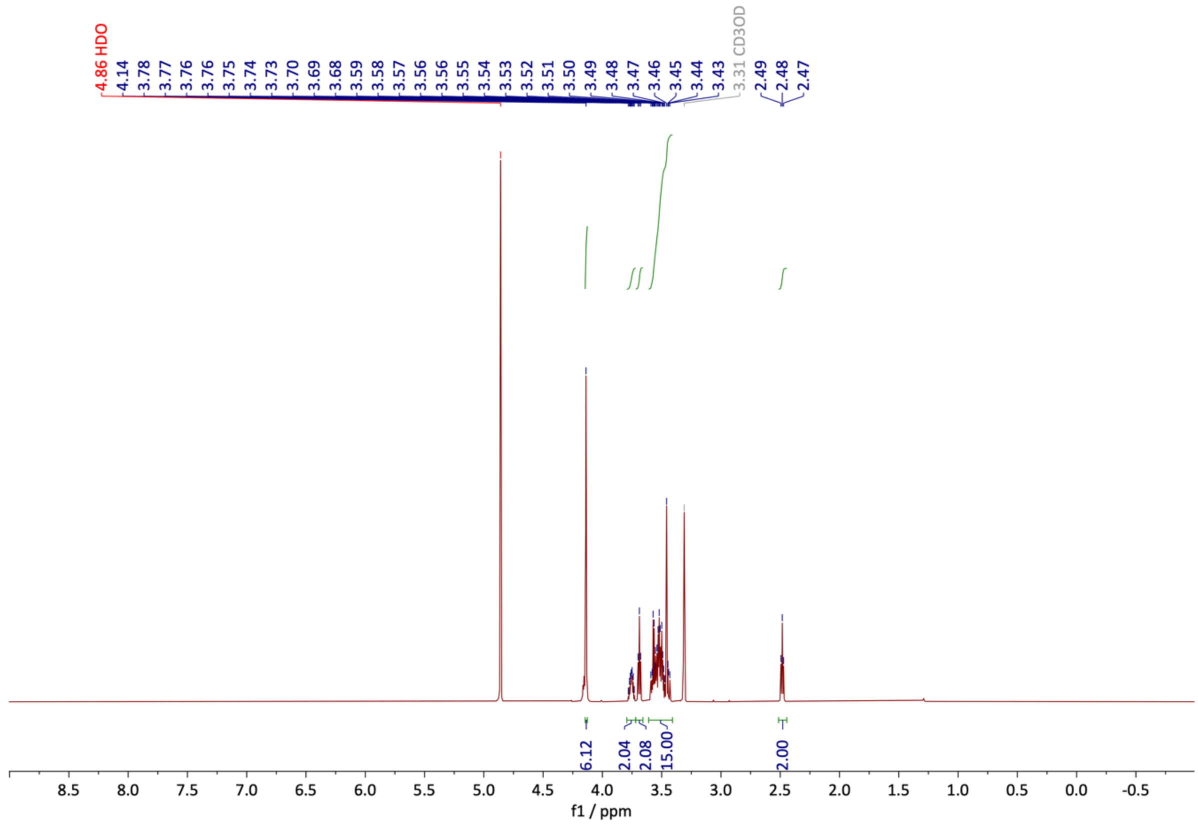

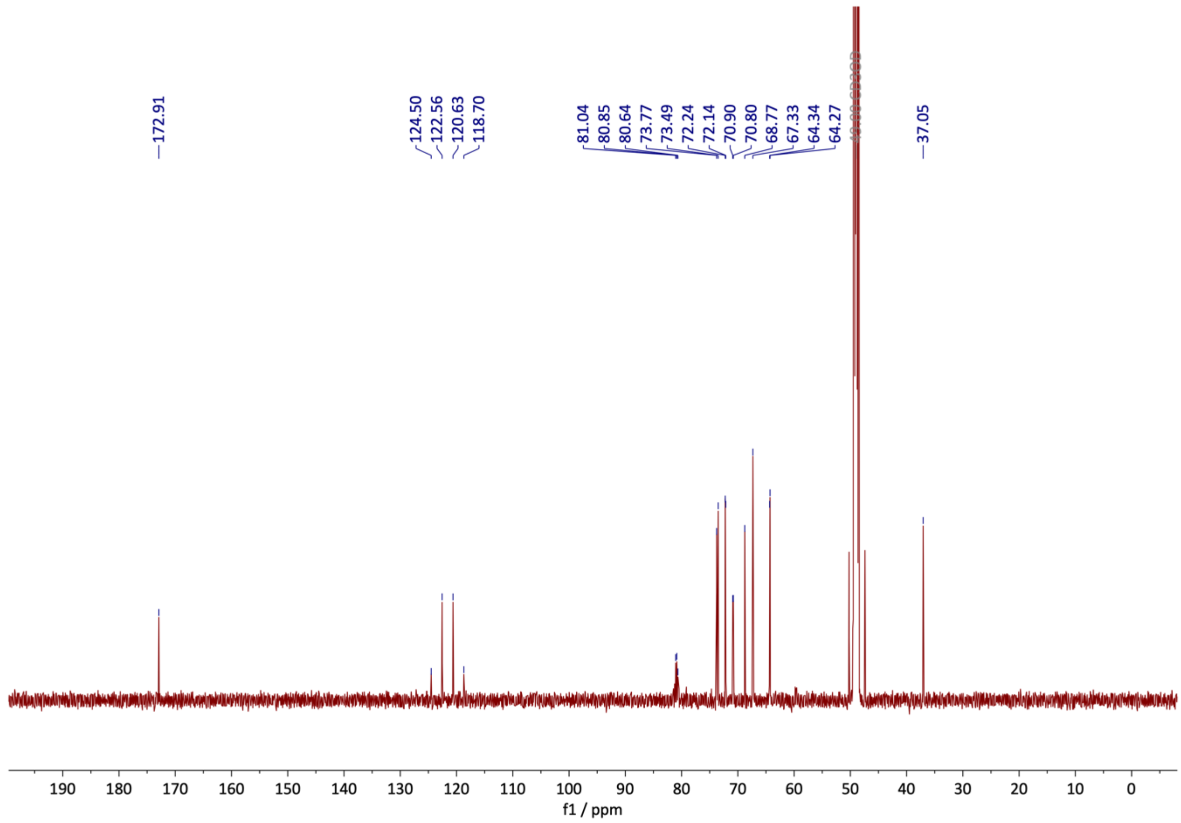

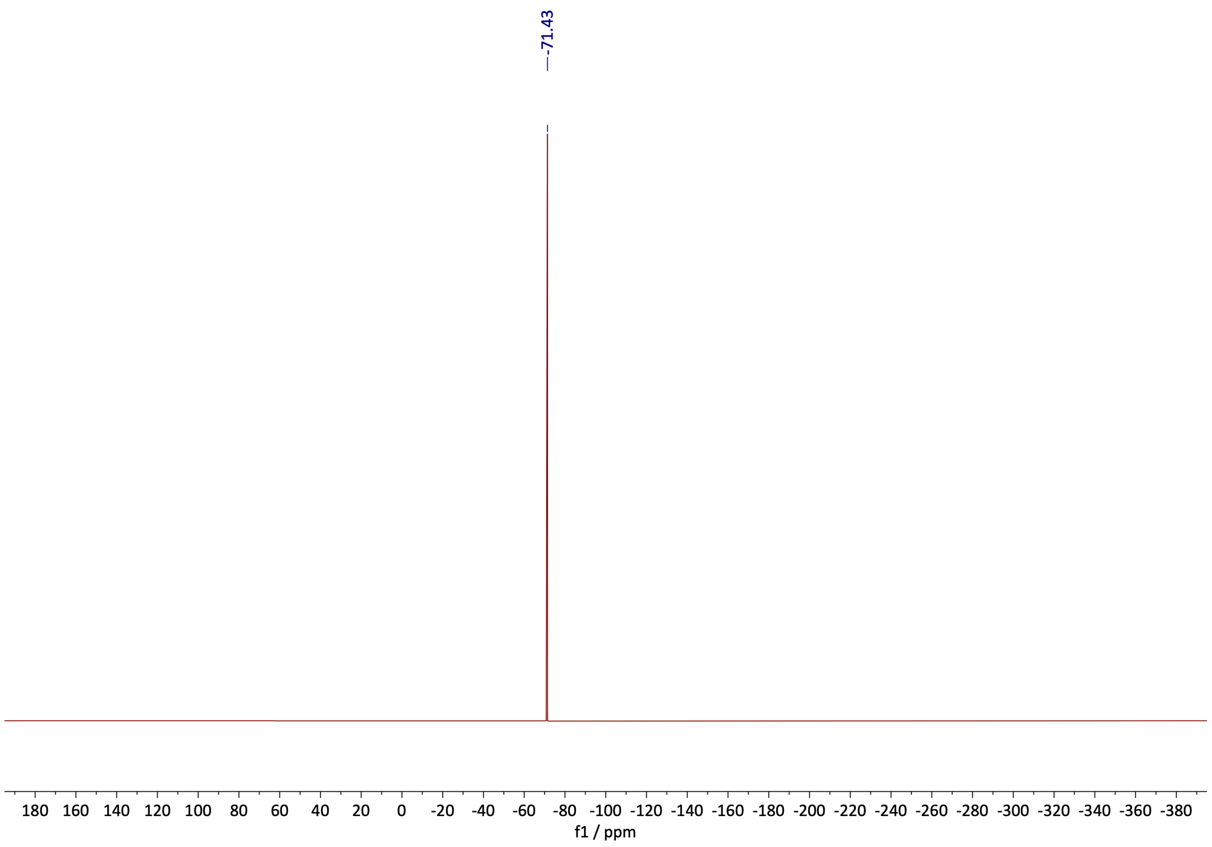
-71.43
180 160 140 120 100 80 60 40 20 0 -20 -40 -60 -80 -100 -120 -140 -160 -180 -200 -220 -240 -260 -280 -300 -320 -340 -360 -380
f1 / ppm

13.2 BFTG1

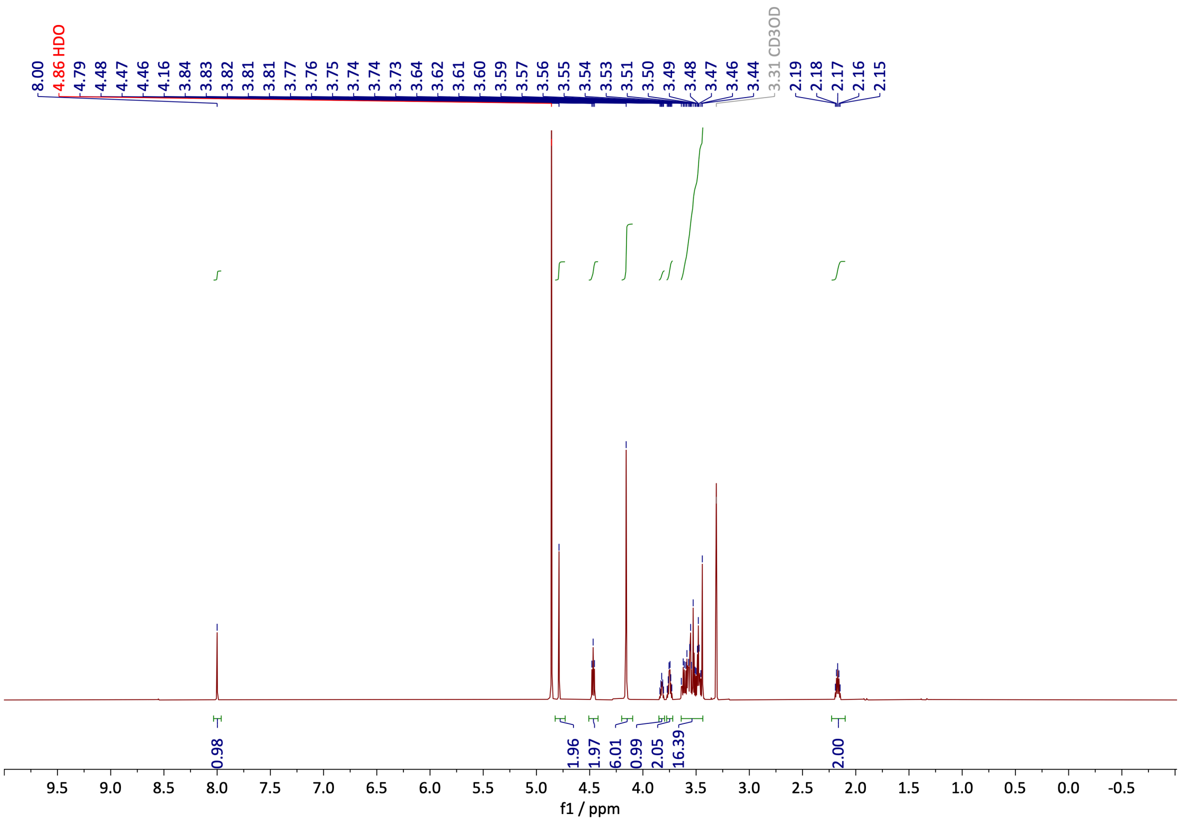

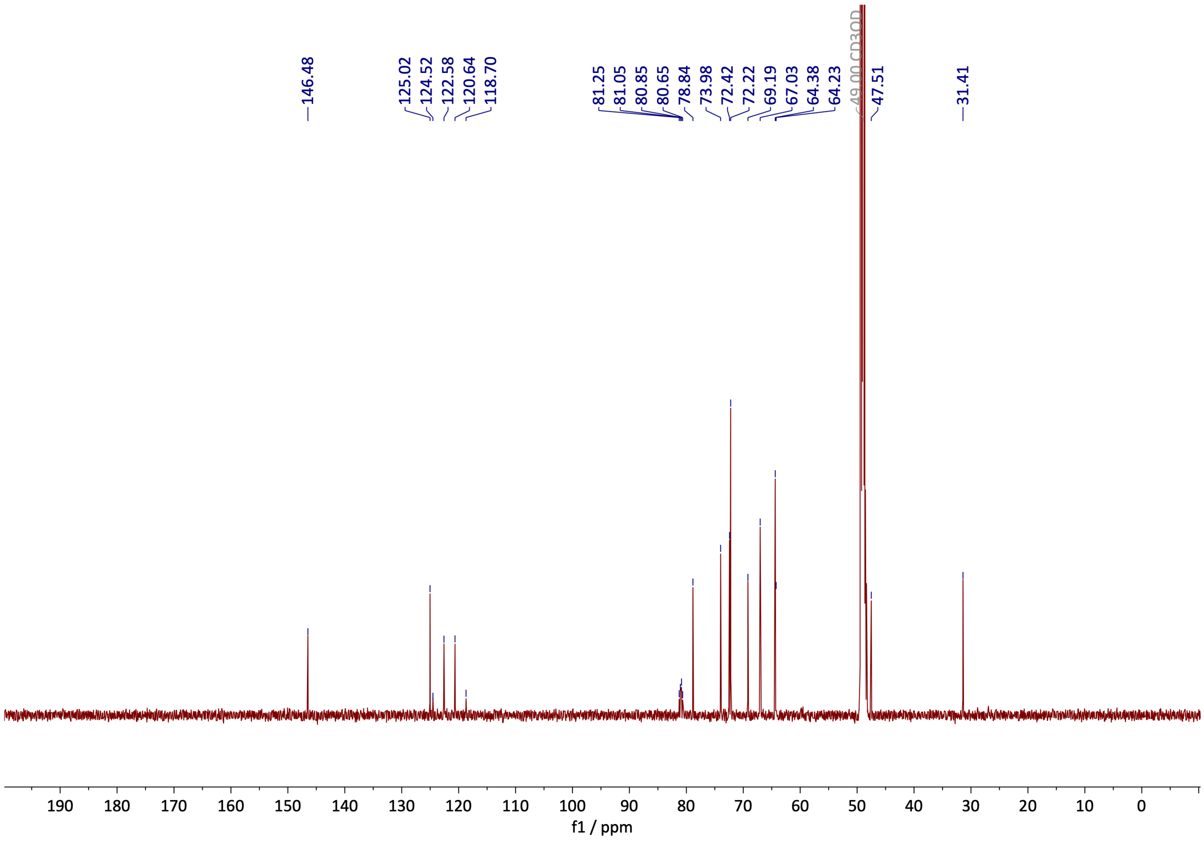

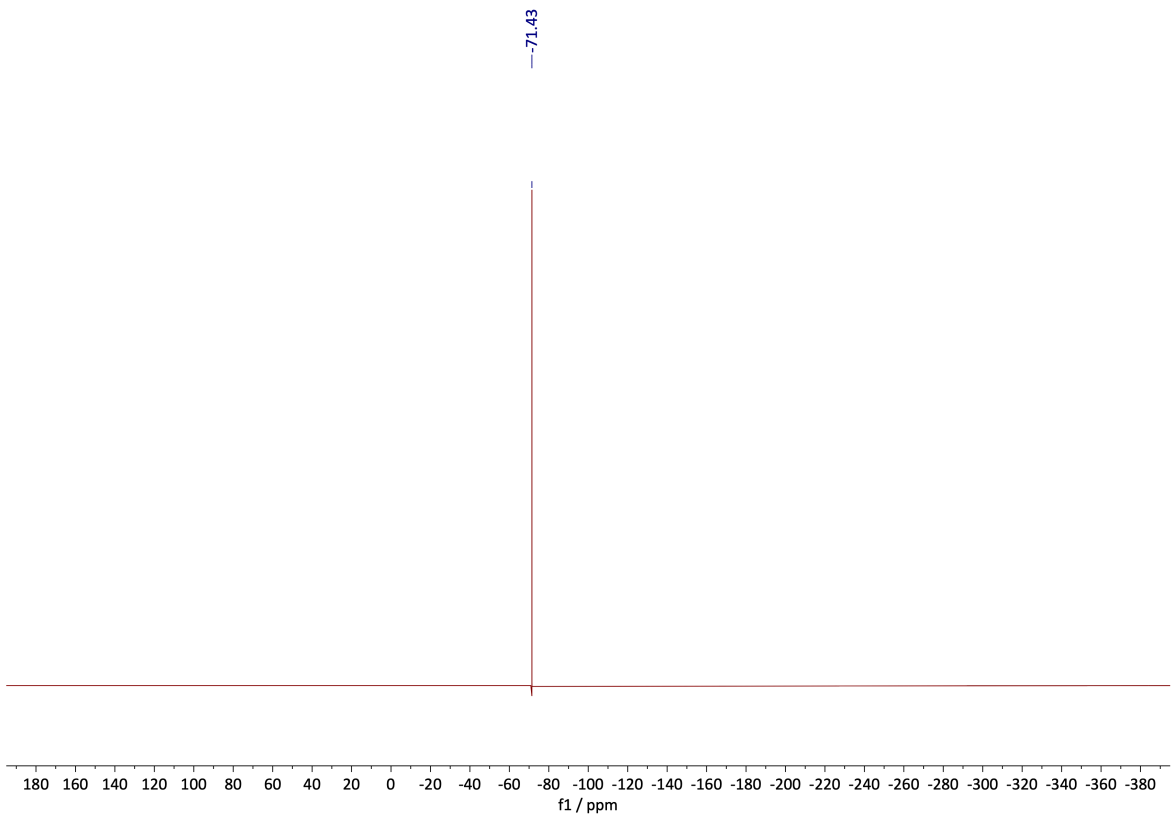

13.3 Compound 6

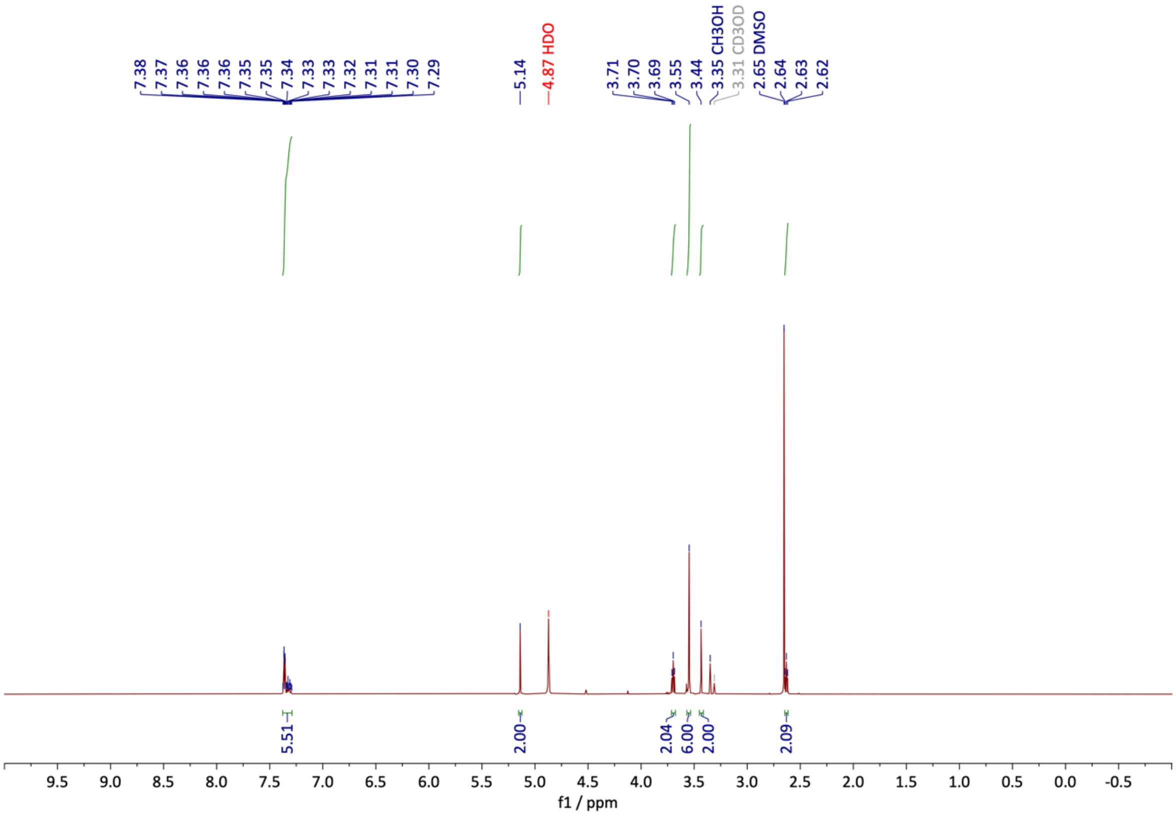

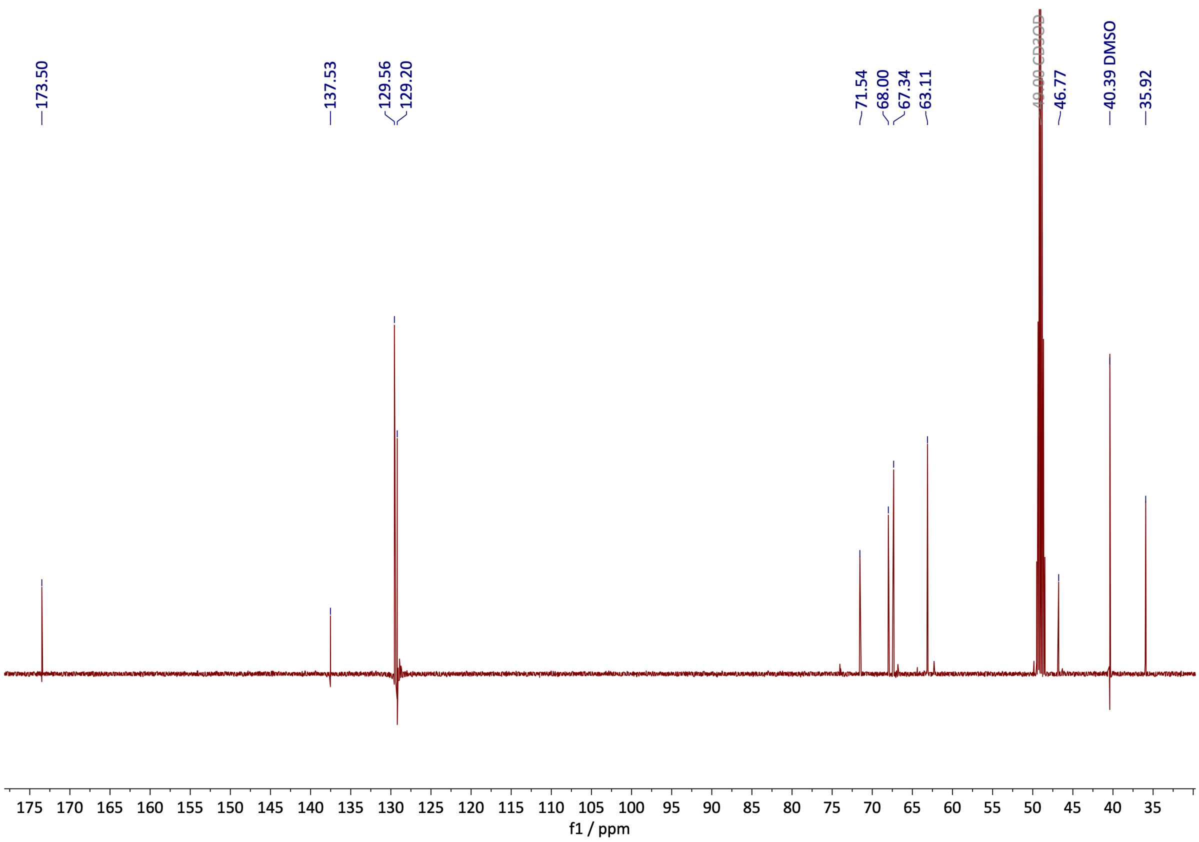

13.4 Compound 7

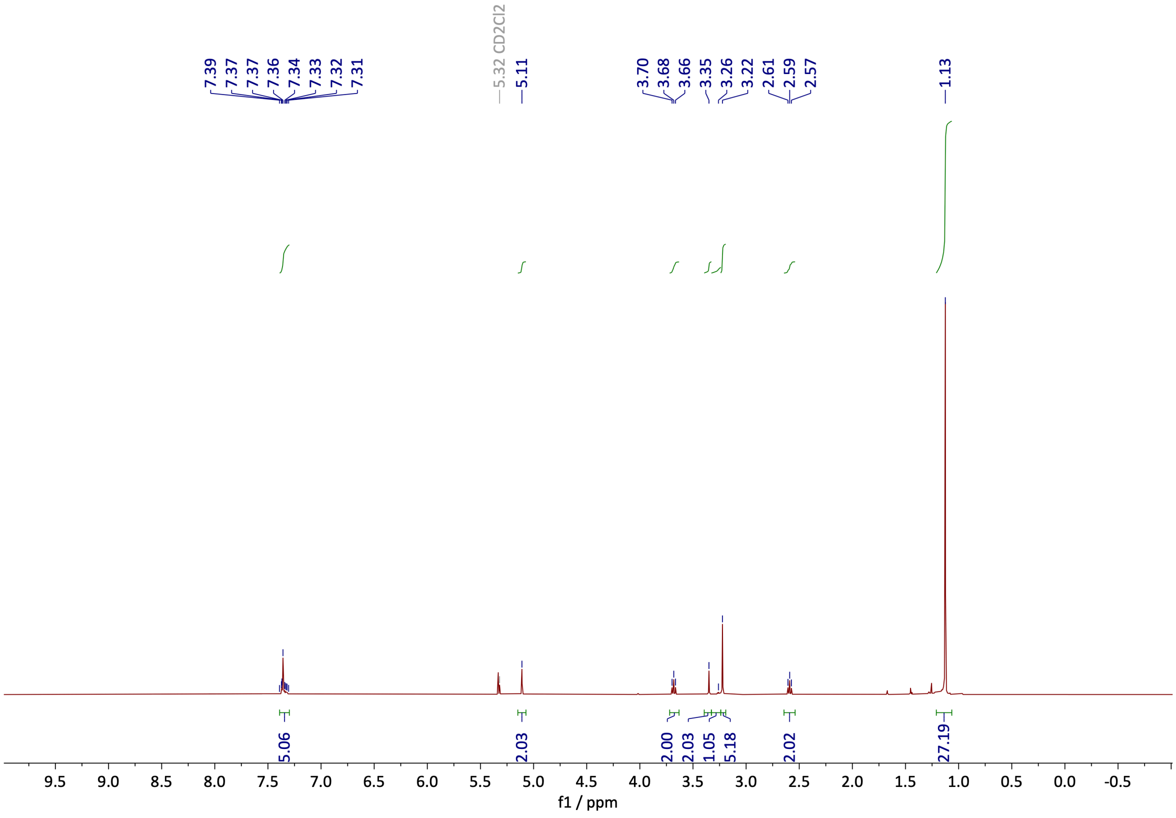

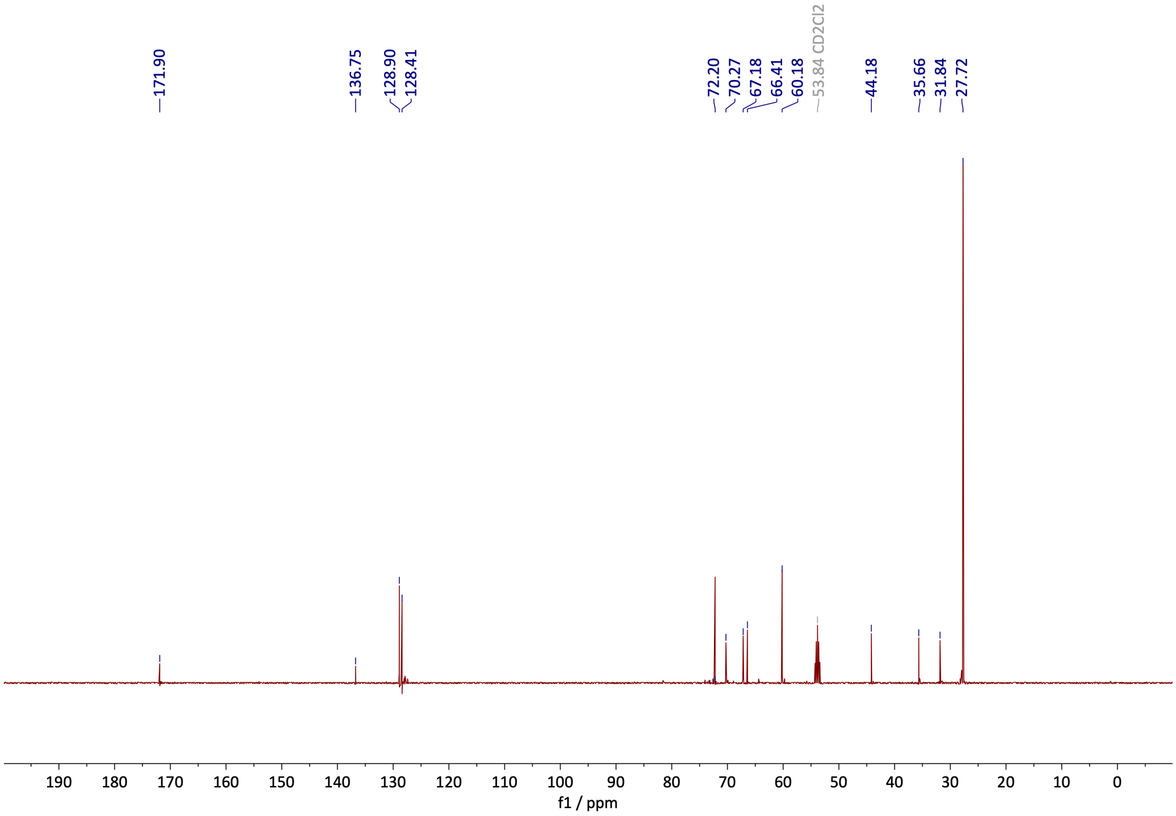

13.5 Compound 8

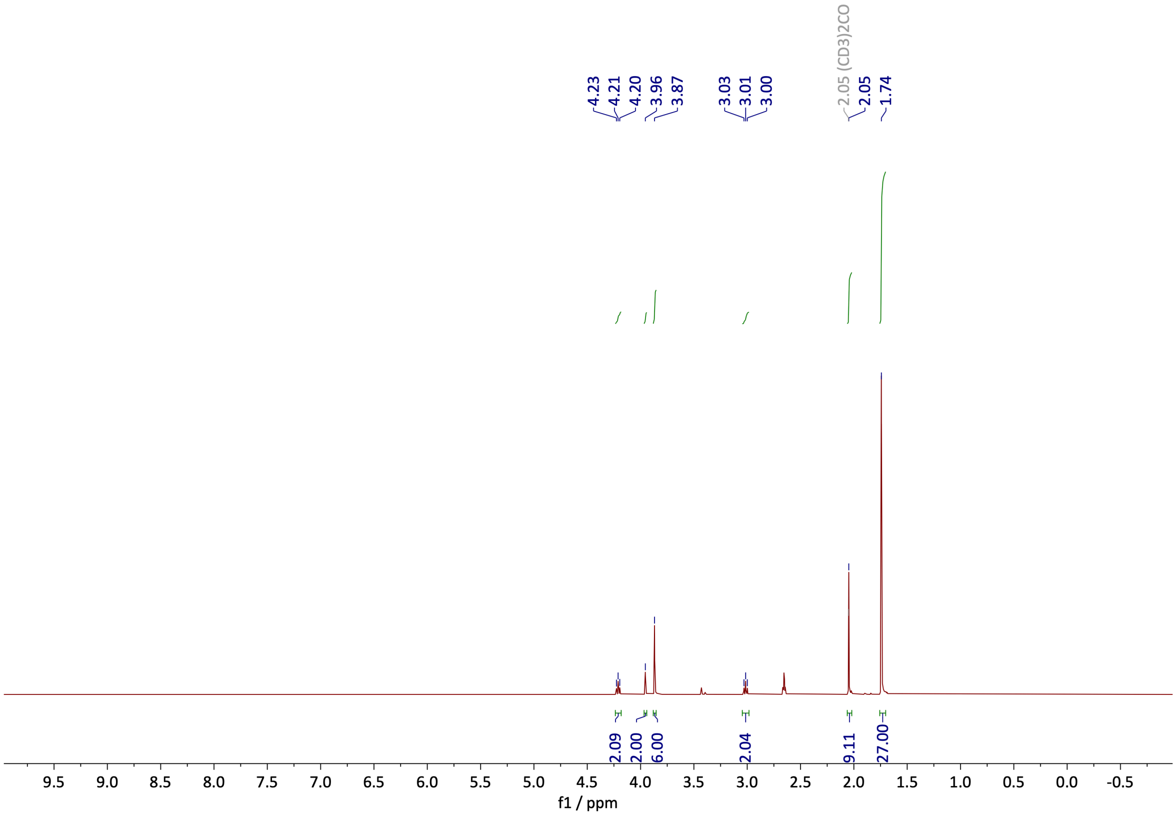

13.6 Compound 9

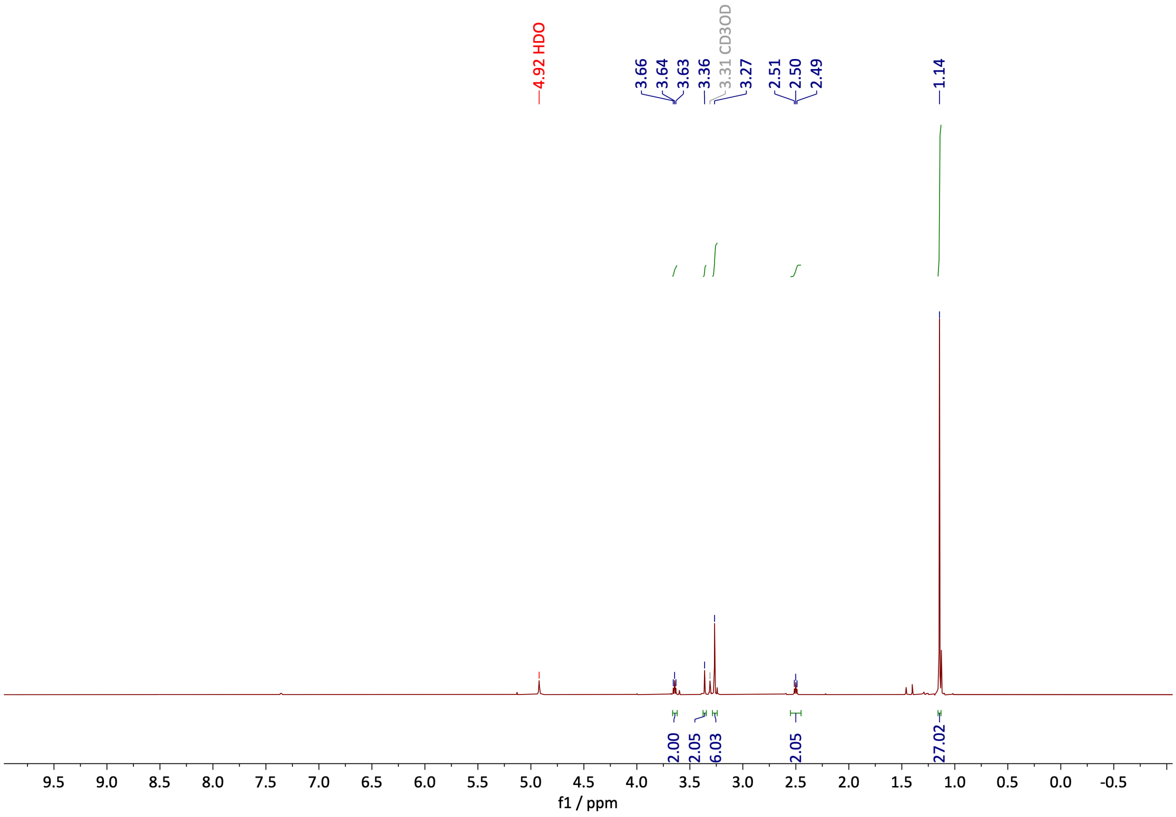

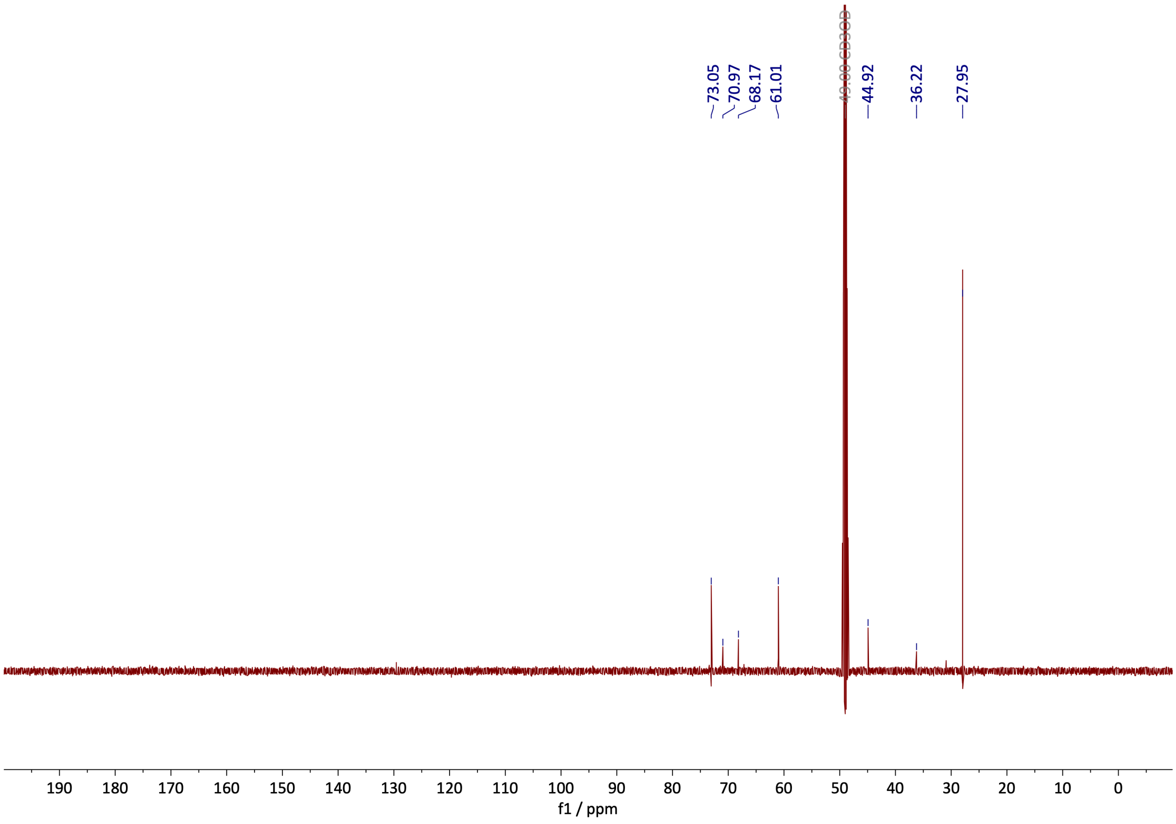

13.7 Compound 10

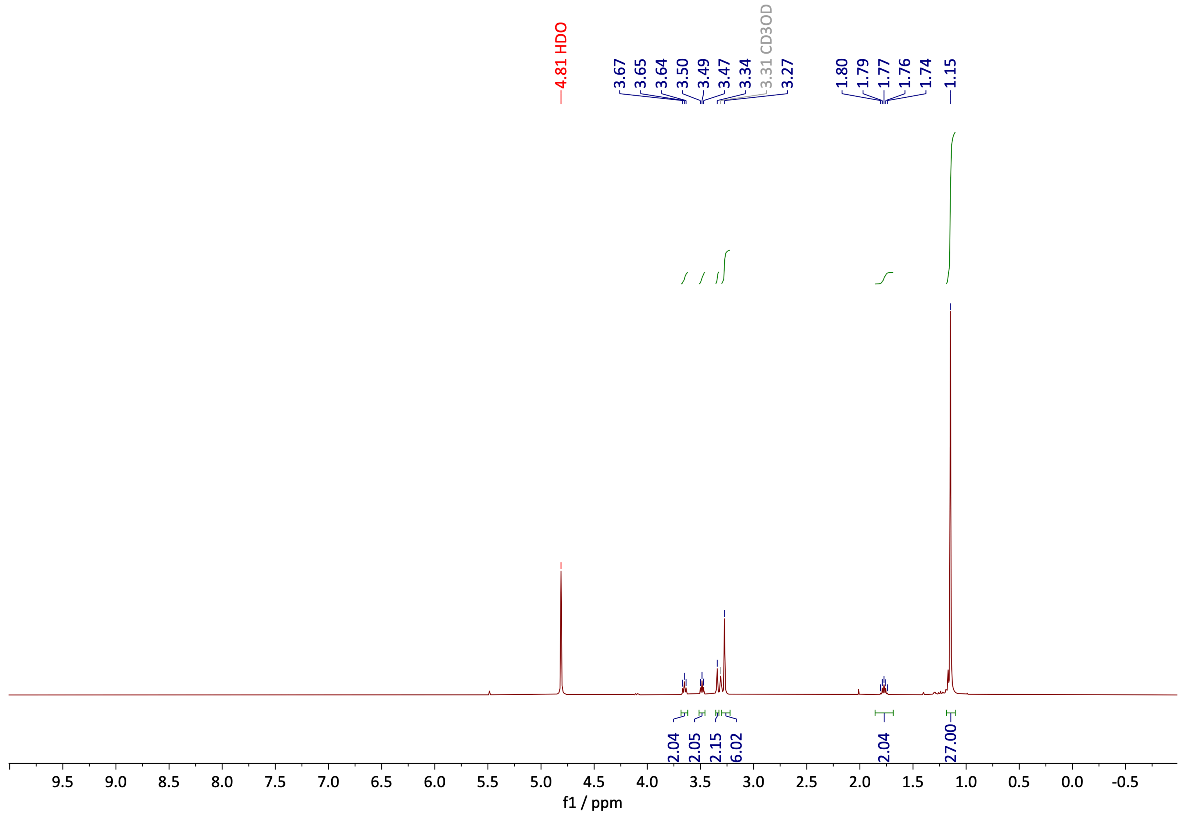

13.8 Compound 11

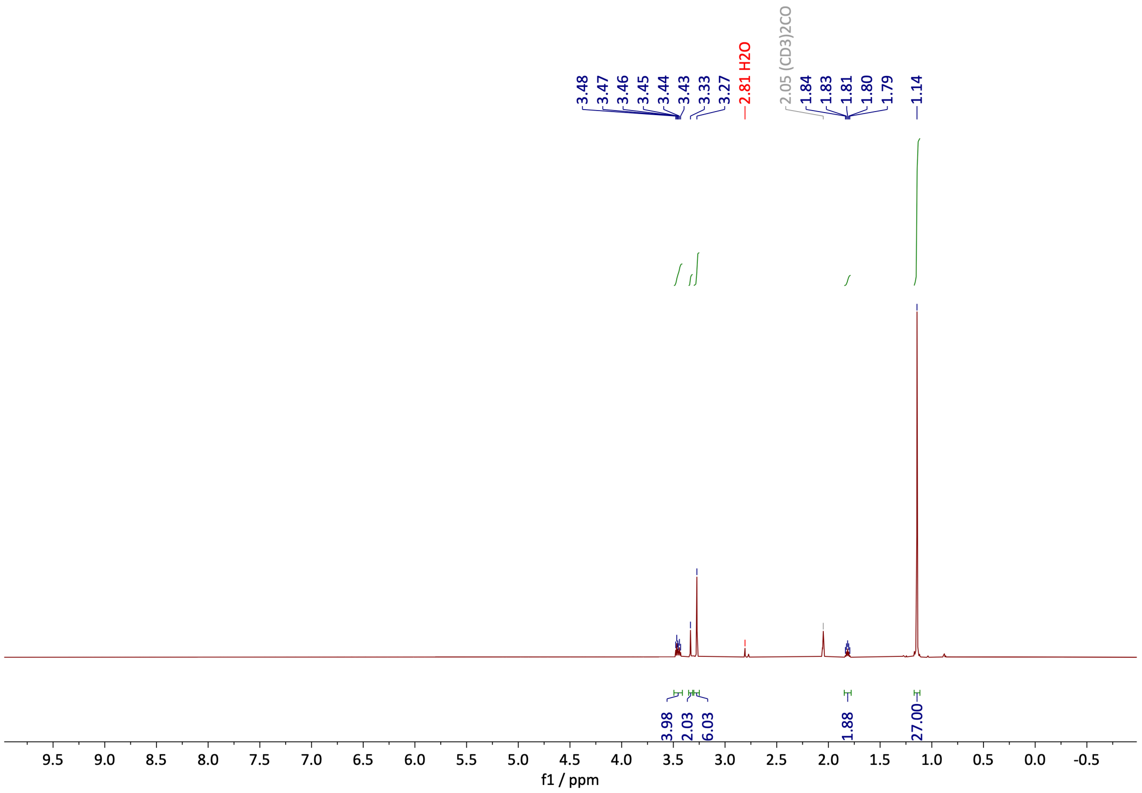

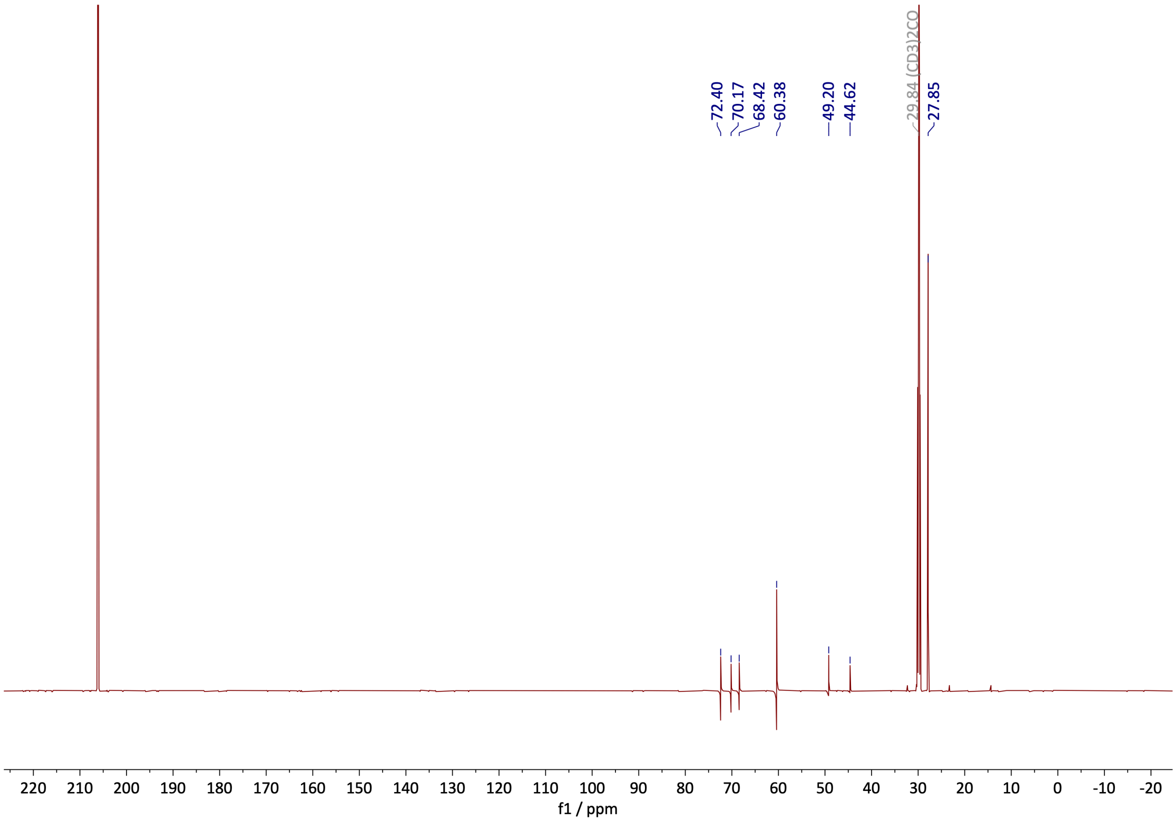
72.40
70.17
68.42
60.38
49.20
44.62
29.84 (CD3)2CO
27.85
f1 / ppm

13.9 BHAG1

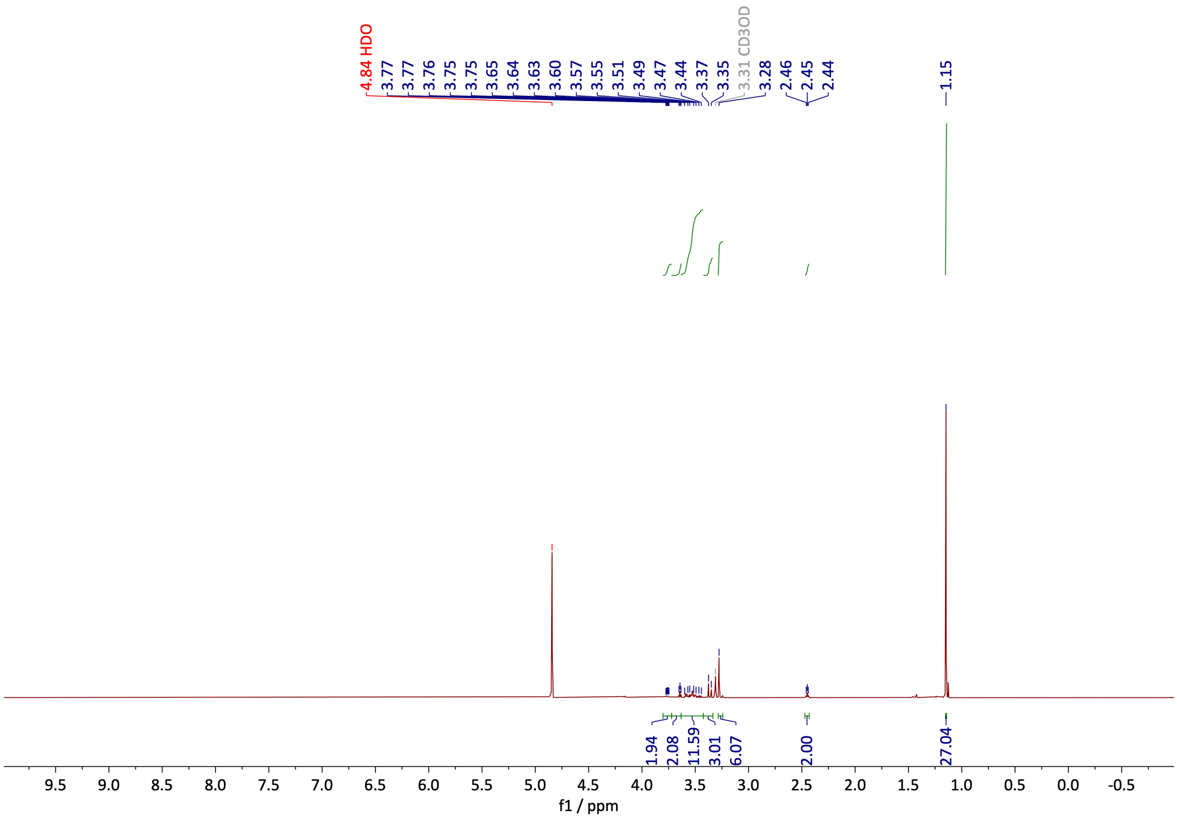
4.84 HDO
3.77
3.77
3.76
3.75
3.65
3.64
3.63
3.60
3.57
3.55
3.51
3.49
3.47
3.44
3.37
3.35
3.31 CD3OD
3.28
2.46
2.45
2.44
1.15
1.94
2.08
11.59
3.01
6.07
2.00
27.04
f1 / ppm

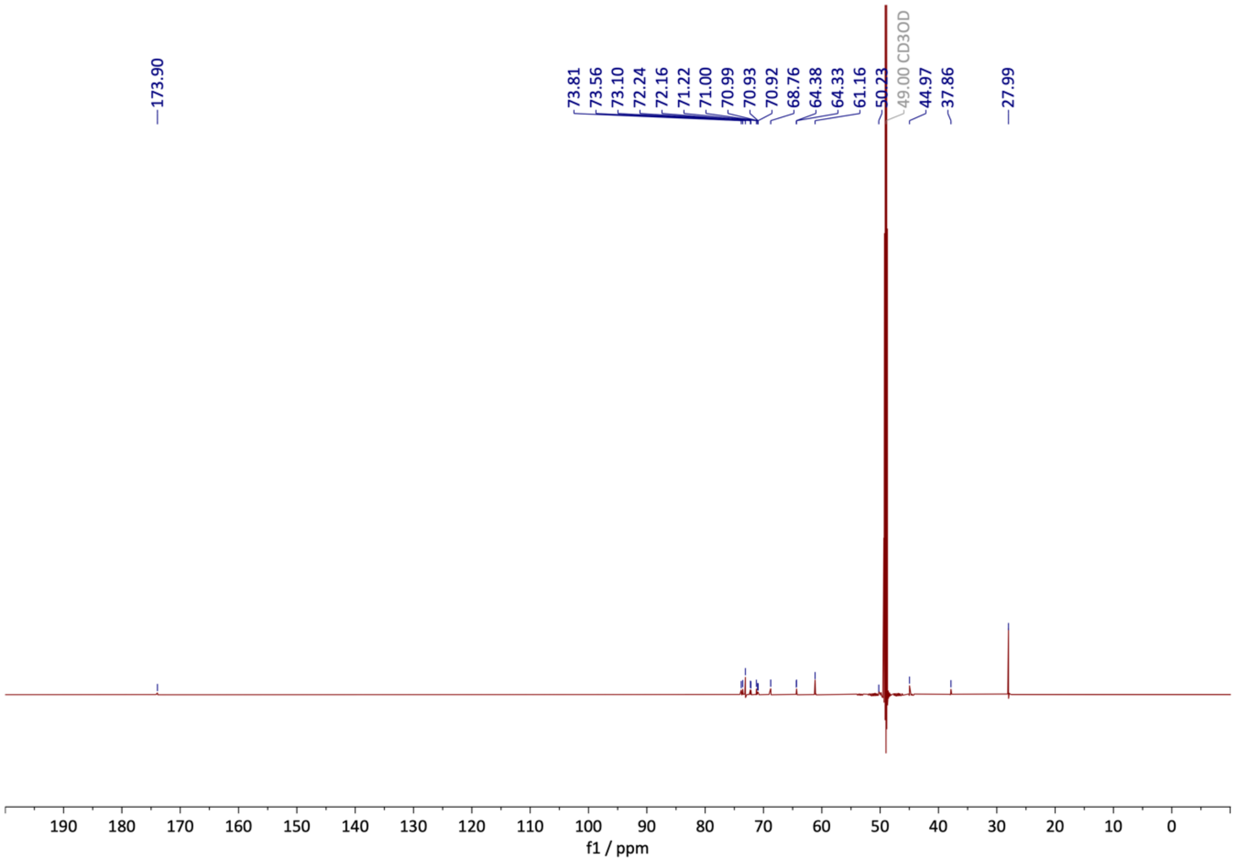
173.90
73.81
73.56
73.10
72.24
72.16
71.22
71.00
70.99
70.93
70.92
68.76
64.38
64.33
61.16
50.23
49.00 CD3OD
44.97
37.86
27.99
f1 / ppm

13.10 BHTG1

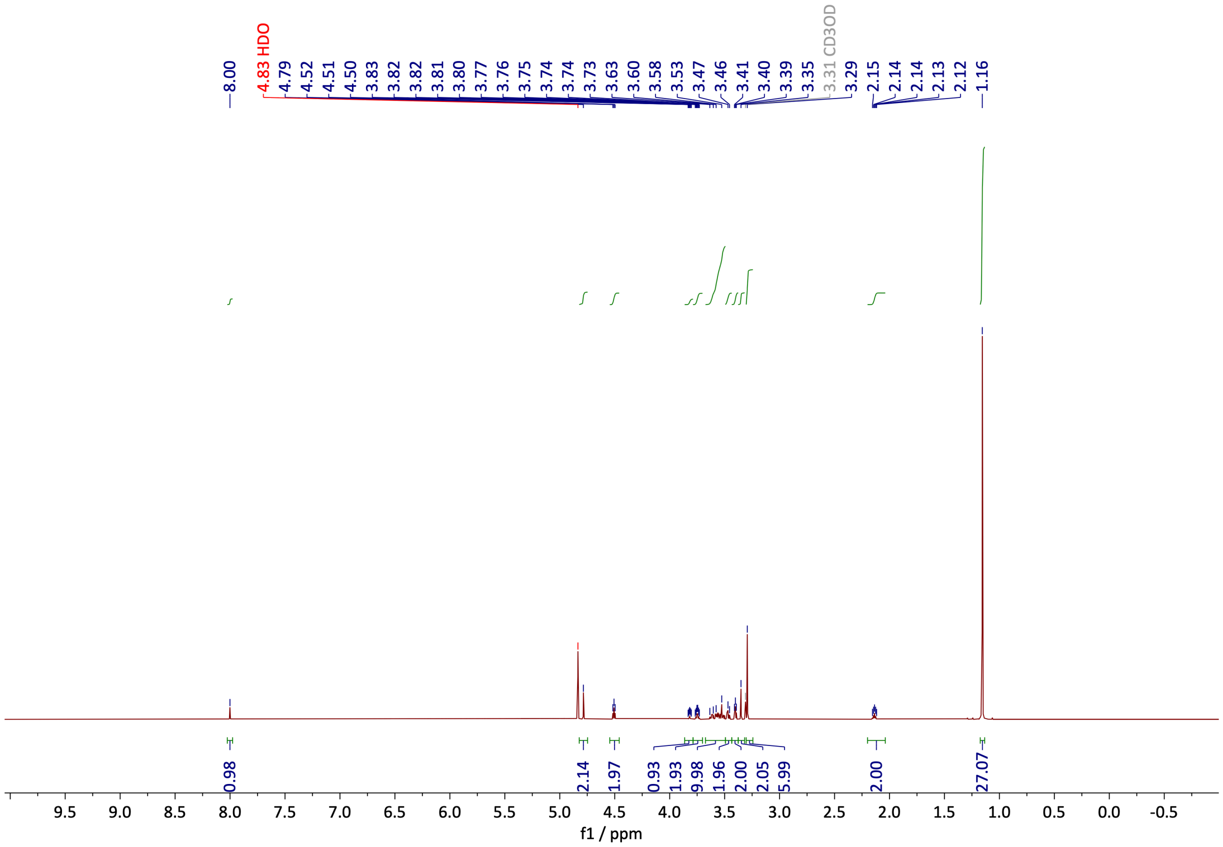
8.00
4.83 HDO
4.79
4.52
4.51
4.50
3.83
3.82
3.82
3.81
3.80
3.77
3.76
3.75
3.74
3.74
3.73
3.63
3.60
3.58
3.53
3.47
3.46
3.41
3.40
3.39
3.35
3.31 CD3OD
3.29
2.15
2.14
2.14
2.13
2.12
1.16
0.98
2.14
1.97
0.93
1.93
9.98
1.96
2.00
2.05
5.99
2.00
27.07
f1 / ppm

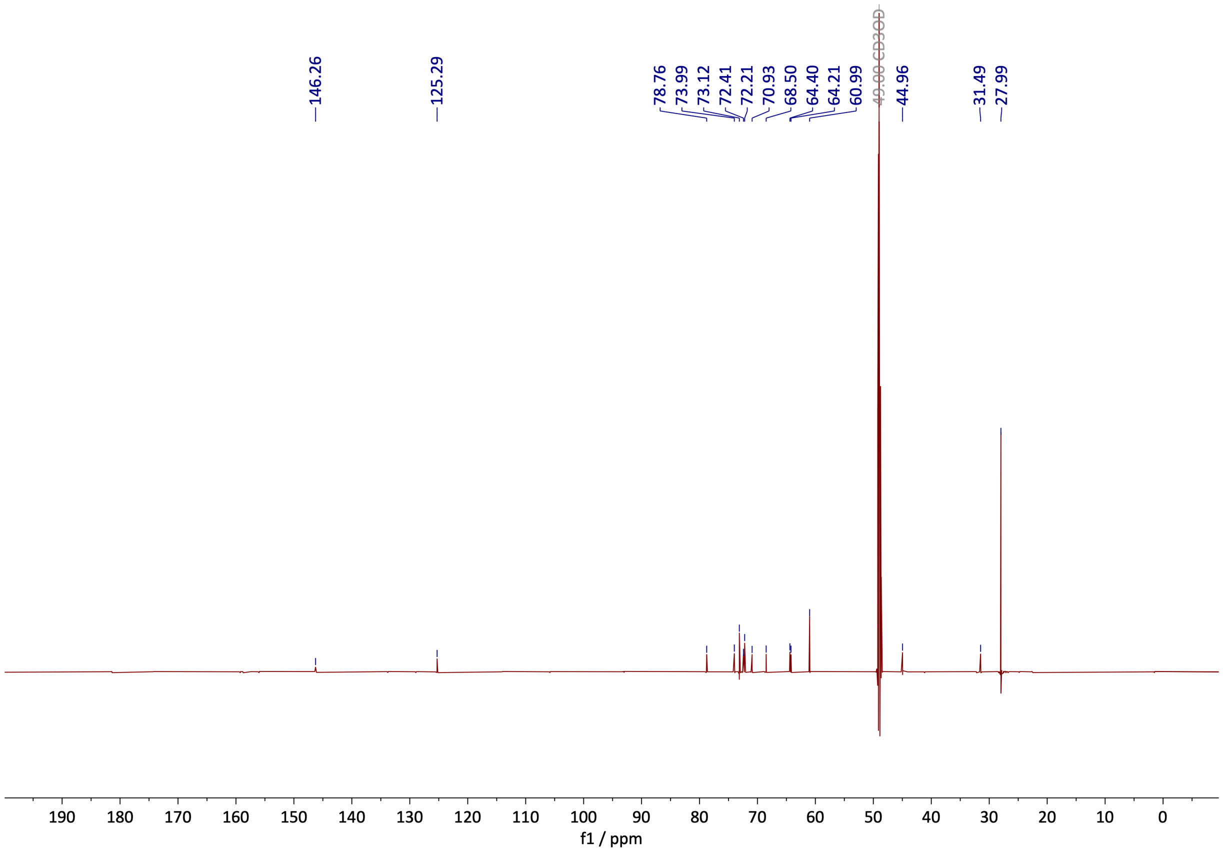

14 References

1) A. K. Singh, B. Schade, M. Rosati, R. Rashmi, V. Dichiarante, G. Cavallo, P. Metrangolo, R. Haag, *Macromol. Biosci*. **2022**, *22*, 11 2200108.
2) B. Hammouda, *Journal of Applied Crystallography* **2010**, *43*, 716-719. DOI 10.1107/s0021889810015773.
3) J. R. Magana, M. Homs, J. Esquena, I. Freilich, E. Kesselman, D. Danino, C. Rodríguez-Abreu, C. Solans, *Journal of Colloid and Interface Science* **2019**, *550*, 73-80. DOI 10.1016/j.jcis.2019.04.084.
4) L. Caselli, L. Conti, I. De Santis, D. Berti, *Advances in Colloid and Interface Science* **2024**, *327*. DOI 10.1016/j.cis.2024.103156.
5) G. Lindblom, L. Rilfors, *Biochimica Et Biophysica Acta* **1989**, *988* (2), 221-256. DOI 10.1016/0304-4157(89)90020-8.
6) O. Glatter, Scattering Methods and their Application in Colloid and Interface Science. Elsevier: Amsterdam, **2018**.
7) A. Angelova, B. Angelov, R. Mutafchieva, S. Lesieur, U. Vainio, V. M. Garamus, G. V. Jensen, J. S. Pedersen, *Physical Chemistry Chemical Physics* **2011**, *13* (8), 3073-3081. DOI 10.1039/c0cp01029d.
8) S. Jo, T. Kim, V. G. Iyer, W. Im, CHARMM-GUI: a web-based graphical user interface for CHARMM. *J. Comput. Chem.* **2008**, *29* (11), 1859-1865.
9) P. C. Souza, R. Alessandri, J. Barnoud, S. Thallmair, I. Faustino, F. Grünewald, I. Patmanidis, H. Abdizadeh, B. M. Bruininks, T. A. Wassenaar, Martini 3: a general purpose force field for coarse-grained molecular dynamics. *Nat. methods* **2021**, *18* (4), 382-388.
10) R. Rashmi, H. Hasheminejad, S. Herziger, A. Mirzaalipour, A. K. Singh, R. R. Netz, C. Böttcher, H. Makki, S. K. Sharma, R. Haag, Supramolecular Engineering of Alkylated, Fluorinated, and Mixed Amphiphiles. *Macromol. Rapid Commun.* **2022**, *43* (8), 2100914.
